# Identification of 2-(*N*-aryl-1,2,3-triazol-4-yl) quinoline derivatives as antitubercular agents endowed with InhA inhibitory activity

**DOI:** 10.3389/fchem.2024.1424017

**Published:** 2024-08-07

**Authors:** Ahmed Sabt, Maha-Hamadien Abdulla, Manal S. Ebaid, Jakub Pawełczyk, Hayam A. Abd El Salam, Ninh The Son, Nguyen Xuan Ha, Mansoor-Ali Vaali Mohammed, Thamer Traiki, Ahmed E. Elsawi, Bozena Dziadek, Jaroslaw Dziadek, Wagdy M. Eldehna

**Affiliations:** ^1^ Chemistry of Natural Compounds Department, Pharmaceutical and Drug Industries Research Institute, National Research Center, Dokki, Egypt; ^2^ Colorectal Research Chair, Department of Surgery, College of Medicine, King Saud University, Riyadh, Saudi Arabia; ^3^ Department of Chemistry, College of Science, Northern Border University, Arar, Saudi Arabia; ^4^ Laboratory of Genetics and Physiology of Mycobacterium, Institute of Medical Biology of the Polish Academy of Sciences, Lodz, Poland; ^5^ Department of Green Chemistry, National Research Center, Dokki, Egypt; ^6^ Institute of Chemistry, Vietnam Academy of Science and Technology (VAST), Hanoi, Vietnam; ^7^ Department of Chemistry, Graduate University of Science and Technology, Hanoi, Vietnam; ^8^ Institute of Natural Products Chemistry, Vietnam Academy of Science and Technology, Hanoi, Vietnam; ^9^ Department of Pharmaceutical Chemistry, Faculty of Pharmacy, Kafrelsheikh University, Kafrelsheikh, Egypt; ^10^ Department of Molecular Microbiology, Faculty of Biology and Environmental Protection, University of Lodz, Lodz, Poland; ^11^ Department of Pharmaceutical Chemistry, Faculty of Pharmacy, Pharos University in Alexandria, Alexandria, Egypt

**Keywords:** quinoline, triazole, biological evaluations, molecular docking, MD simulation

## Abstract

The spread of drug-resistant tuberculosis strains has become a significant economic burden globally. To tackle this challenge, there is a need to develop new drugs that target specific mycobacterial enzymes. Among these enzymes, InhA, which is crucial for the survival of *Mycobacterium tuberculosis*, is a key target for drug development. Herein, 24 compounds were synthesized by merging 4-carboxyquinoline with triazole motifs. These molecules were then tested for their effectiveness against different strains of tuberculosis, including *M. bovis BCG*, *M. tuberculosis*, and *M. abscessus*. Additionally, their ability to inhibit the InhA enzyme was also evaluated. Several molecules showed potential as inhibitors of *M. tuberculosis*. Compound **5n** displayed the highest efficacy with a MIC value of 12.5 μg/mL. Compounds **5g**, **5i**, and **5n** exhibited inhibitory effects on InhA. Notably, **5n** showed significant activity compared to the reference drug Isoniazid. Molecular docking analysis revealed interactions between these molecules and their target enzyme. Additionally, the molecular dynamic simulations confirmed the stability of the complexes formed by quinoline-triazole conjugate **5n** with the InhA. Finally, **5n** underwent *in silico* analysis to predict its ADME characteristics. These findings provide promising insights for developing novel small compounds that are safe and effective for the global fight against tuberculosis.

## 1 Introduction

Since its identification in 1882, *Mycobacterium tuberculosis* (MTB), commonly referred to as Koch’s *bacillus*, has continued to exert a significant impact on global health (Barberis et al., 2017). Tuberculosis (TB), caused by the bacterium MTB is consistently listed among the leading 10 causes of mortality worldwide (Ravimohan et al., 2018). At present, approximately 1.7 billion people, constituting 23% of the world’s population, grapple with MTB, resulting in over 10 million new TB cases annually (Daley, 2019). Current TB treatment is lengthy, arduous, and associated with numerous side effects; a course of antibiotics is typically prescribed for a duration of 6–9 months for drug-susceptible tuberculosis, while cases of multidrug-resistant tuberculosis (MDR-TB) or instances of emerging drug resistance may necessitate treatment durations of 9–20 months (Esmail et al., 2014). A strain of tuberculosis that exhibits resistance to the two primary drugs, rifampicin and isoniazid (INH), is classified as MDR-TB (Seung et al., 2015). Conversely, the extensively drug-resistant tuberculosis strain (XDR-TB) represents an MDR-TB form that is resistant not only towards additional fluoroquinolones but also to at least one of levofloxacin, moxifloxacin, bedaquiline, and linezolid (WHO Report, 2020).

TB agents cannot reach the target site due to the intricate structure and poor permeability of mycobacteria’s cell envelope (Jackson, 2014). Fatty acid synthase type I and type II (FAS-I and FAS-II) control the synthesis of the mycobacterial cell envelope (Marrakchi et al., 2000; Lu and Tonge, 2008). While FAS-I is exclusive to eukaryotic cells, the FAS-II enzyme emerges as a viable candidate for pharmaceutical development. Enoyl acyl carrier protein reductase (InhA), an enzyme of the FAS-II system, assumes a pivotal role in the saturation of double bonds in fatty acid chains linked to acyl carrier protein (ACP) (Rožman et al., 2017). Isoniazid, a primary treatment for tuberculosis, inhibits the enzyme InhA, thereby blocking mycolic acid production (Dessen et al., 1995). However, for INH to exhibit its pharmacological activity, it must undergo an activation process facilitated by the catalase-peroxidase enzyme KatG. Such a process entails the covalent INH binding to the NADH cofactor located in the InhA binding cavity (Munir et al., 2021). Over time, different strains of MTB have acquired resistance to INHdue to genetic mutations in the KatG gene (Muthaiah et al., 2017). Consequently, researchers have been motivated to explore new compounds that directly target InhA without relying on KatG activation (Almeida Da Silva and Palomino, 2011).

**FIGURE 1:**
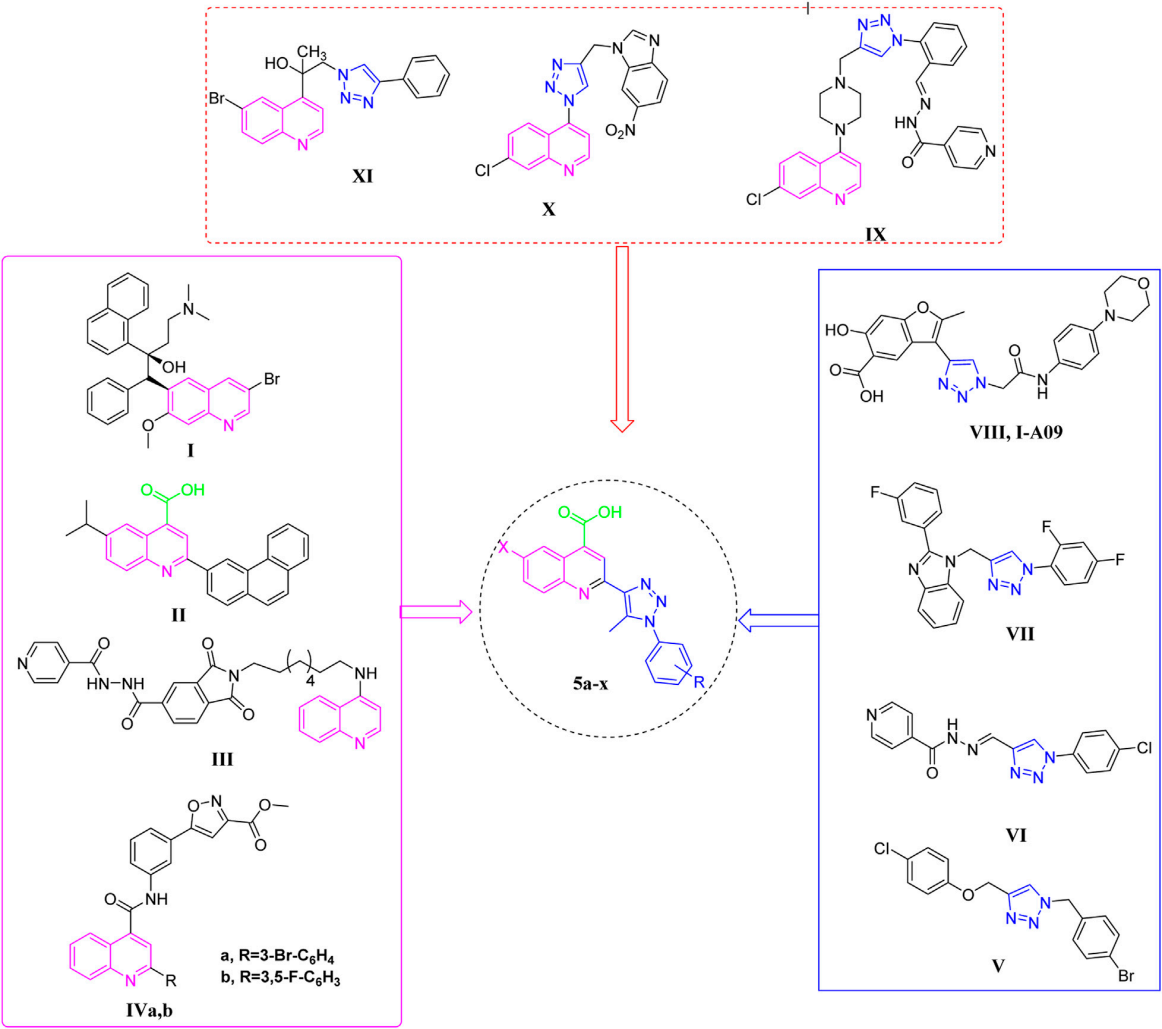
Reported antitubercular agents containing bioactive cores quinoline (**I-IV)**, triazoles (**V-VIII)**, quinoline-triazole hybrids (**IX-XI)**, and our newly designed compounds (5a-x).

Quinoline is a commonly occurring structural framework present in many natural anti-tuberculosis products and medications (Keri and Patil, 2014), besides its diverse biological effects (Mohamede et al., 2015; Abdelrahman et al., 2022; Elbadawi et al., 2022; Elkaeed et al., 2022; Sabt et al., 2023a; Sabt et al., 2023b; Khaleel et al., 2024). Bedaquiline **I** (TMC207, Sirturo) (Figure 1) which contains a diarylquinoline core, received approval from the US-FDA for the treatment of pulmonary MDR-TB, marking the end of a 40-year delay. As an inhibitor of ATP synthase, this compound exhibits remarkable potency against both replicating and non-replicating strains (Pym et al., 2016). 1n 2023, Quimque and colleagues (Quimque et al., 2023) created and synthesized new arylated quinoline carboxylic acids (QCAs) that effectively inhibited the pathogen MTB. Compound **II** (Figure 1) was found to have the highest potency, with a minimum inhibitory concentration (MIC) of around 16 μM. Furthermore, anti-mycobacterial action was also demonstrated for 4-aminoquinoline-isoindolindione-isoniazidhybrid **III** (Figure 1), with a MIC of 5.1 µM (Zhou et al., 2010). Yaddanapudi and colleagues (Kumar Sahoo et al., 2022) exploited the quinoline motif and combined it with isoxazole alkyl ester to synthesize potent hybrid compounds **IVa-b** (Figure 1) with high activity towards MTB, exhibiting a MIC value of 1 μg/mL.

In recent times, triazole tethered small molecules have emerged as a significant category of organic compounds due to their diverse array of biological applications, e.g., anti-tubercular (Dhameliya et al., 2023), antibacterial (Li and Zhang, 2022), anti-viral (Senthil et al., 2015), and anticancer (Said et al., 2020; Azab et al., 2022; Elsawi et al., 2023; Elsawi et al., 2024). Notably, derivatives of 1,2,3-triazole have demonstrated promising anti-tubercular activity, prompting the development of numerous synthetic methodologies for their production (Zhang et al., 2017; El-Shoukrofy et al., 2023). Recent research has focused on the synthesis of a spectrum of small molecules containing conjugated 1,2,3-triazoles, which have exhibited various bioactivities. For instance, Shingate and colleagues have reported the inhibitory activities of 1,4-disubstituted 1,2,3-triazole-based molecules (Compound **V**, Figure 1) against MTB (Shaikh et al., 2015). Additionally, the clubbed 1,2,3-triazoles with INH, such as compound **VI** (Figure 1), have been identified as inhibitors of the MTB H37Rv strain MIC = 0.62 ug/mL (Boechat et al., 2011). Furthermore, the hybridization of fluorine-containing benzimidazole series and triazoles resulted in compound **VII** (Figure 1) that have also been reported to be a potent inhibitor of tuberculosis with MIC = 6.25 ug/mL (Gill et al., 2008). Notably, in clinical trials, **I-A09**, a compound containing triazole (**VIII**, Figure 1) is being studied as an anti-TB drug (Dadlani et al., 2022). In addition, certain quinoline-appended triazoles **IX-XI** (Figure 1) showed potent antitubercular actions (Alcaraz et al., 2022; Nyoni et al., 2023; Shinde et al., 2023).

Based on the facts mentioned above, along with the biological importance of quinoline and 1,2,3-triazole cores, coupled with the ongoing exploration for novel anti-infective compounds as potential anti-tubercular agents, the present investigation aims to explore the potential inhibitory impacts for 4-carboxy quinolino-triazole hybrids on the InhA target as anti-tuberculous agents. The current work employs a molecular hybridization strategy to design these molecules with the potential inhibitory antitubercular effects. The research involves the synthesis of a range of derivatives of 4-carboxy quinoline core, each featuring distinct substituents within the triazole moiety, such as halogens, methoxy, and nitro. Subsequently, the synthesized compounds undergo assessment for their effectiveness against various strains such as *Mycobacterium bovis BCG*, *M. abscesses*, and *M. tuberculosis*. The most potent molecules are subjected to further scrutiny for the inhibitory efficacy towards the MTB InhA. Furthermore, molecular docking investigations, as well as molecular dynamics (MD) studies, are performed to scrutinize the interactions between these biologically active analogues and the InhA enzyme. Lastly, the pharmacokinetics parameters for select analogues undergo exploration utilizing web-based ADMET predictors.

## 2 Results and discussion

### 2.1 Organic chemistry work

**SCHEME 1:**
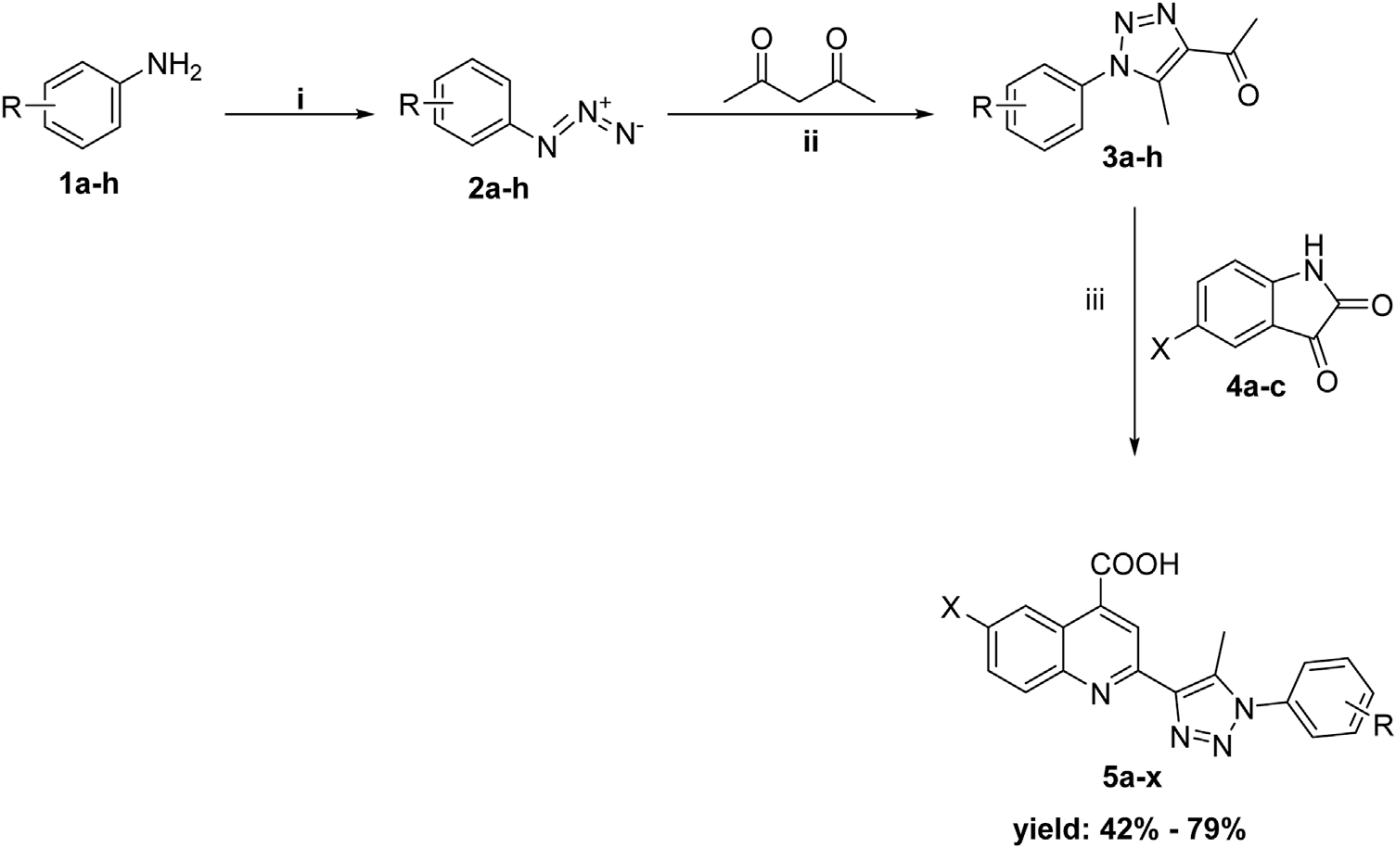
Synthesis of the target conjugates (**5a-x**); Reagents and conditions: (**i**) a) NaNO_2_/HCl/0–5°C,1h, then r.t 1h b) NaN_3_/stirring/rt/3h; (**ii**) K_2_CO_3_/EtOH/reflux; (**iii**) a) aq KOH/EtOH/reflux for 12 h, b) H_3_O^+^.

The synthetic procedures used to prepare the quinolino-triazole hybridized molecules **5** are described in Scheme 1. As previously reported, the key intermediates acetyl triazoles **3a-h** were prepared *via* a 1,3-dipolar cycloaddition reaction (Bekheit et al., 2021), that was performed through the diazotization of aniline derivatives **1a-h** before reacting with sodium azide to yield the corresponding azido derivatives **2a-h**. The reaction of azido compounds **2a-h** with acetylacetone was done in the presence of anhydrous potassium carbonate in refluxing ethanol, producing acetyl triazole derivatives **3a-h**. The desired products, quinoline triazole conjugates **5a-x**, were synthesized using Pfitzinger conditions (Elghamry and Al-Faiyz, 2016) by refluxing acetyl triazole derivatives **3a-h** with the isatin derivatives **4a-c** and adding an aqueous solution of KOH, then acidifying with dilute hydrochloric acid producing the 4-carboxyquinoline-triazole derivatives **5a-x**. The newly synthesized compounds underwent microanalyses and spectrum analysis, including 1H-NMR and 13C-NMR. The collected data aligned with the predetermined structures of the produced molecules.

### 2.2 Biological activities

#### 2.2.1 Antimycobacterial assessment

All carboxy quinoline triazole compounds underwent assessment for potential anti-tubercular efficacy towards tubercle bacilli, encompassing both *M. bovis* BCG and MTB, as well as nontuberculous opportunistic pathogen represented by fast-growing mycobacteria, notably *M. abscessus*. This type of mycobacteria is characterized by a heightened resistance to most anti-tuberculous drugs (Johansen et al., 2020). The primary screening was performed for all compounds at the same concentration of 125 μg/mL. None of the compounds displayed an anti-tubercular effect at the tested concentration against *M. abscessus*. Conversely, all 24 tested compounds suppressed the development of *M. bovis BCG* and MTB. Next, using MABA (microplate alamar blue assay), the MICs against MTB were assessed for all the reported molecules (Table 1). The majority of the compounds investigated demonstrated favorable to moderate anti-tubercular activity against MTB.

**TABLE 1:** The anti-tubercular effectiveness (MICs; µg/mL) of the reported compounds (**5a-x**) tested toward *Mycobacterium bovis* BCG, *M. tuberculosis,* and *M. abscessus* strains.

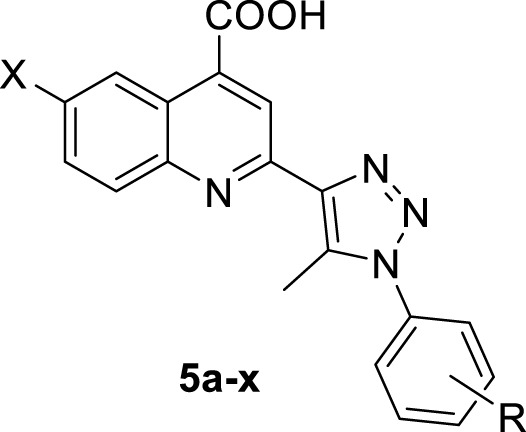						
			MIC [µg/mL]			IC_*50*_ -L929/MIC_*Mtb*_
Compounds	X	R	*M. bovis* BCG	*M. tuberculosis*	*M. abscessus*	
**5a**	H	H	<125	125	>125	ND
**5b**		4-OCH_3_	<125	62.5	>125	ND
**5c**		3-Cl	<125	62.5	>125	ND
**5d**		3-Br	<125	62.5	>125	ND
**5e**		4-F	<125	125	>125	ND
**5f**		4-Cl	<125	62.5	>125	ND
**5g**		4-Br	<125	15	>125	20
**5h**		4-NO_2_	<125	125	>125	ND
**5i**	Cl	H	<125	15	>125	20
**5j**		4-OCH_3_	<125	15	>125	10
**5k**		3-Cl	<125	15	>125	13
**5L**		3-Br	<125	15	>125	13
**5m**		4-F	<125	62.5	>125	ND
**5n**		4-Cl	<125	12.5	>125	16
**5o**		4-Br	<125	15	>125	10
**5p**		4-NO_2_	<125	62.5	>125	ND
**5q**	Br	H	<125	15	>125	ND
**5r**		4-OCH_3_	<125	15	>125	5
**5s**		3-Cl	<125	15	>125	13
**5t**		3-Br	<125	15	>125	13
**5u**		4-F	<125	62.5	>125	ND
**5v**		4-Cl	<125	15	>125	13
**5w**		4-Br	<125	15	>125	10
**5x**		4-NO_2_	<125	125	>125	ND
**INH**			0.05	0.1	0.1	

ND, not determine.

MIC, IC_50_ – cytotoxicity index for L929 cell line.

The derivative **5n** exhibited the most potent anti-tubercular effect toward MTB, with an MIC value of 12.5 μg/mL. Conversely, twelve of the compounds investigated **(5g**, **5i-l**, **5o**, **5q-t**, **5v**, and **7w)** demonstrated effective activity, with an MIC value equal 15 μg/mL. Additionally, seven compounds (**5b-d**, **5f**, **5m**, **5p**, and **5u**) displayed moderate anti-tubercular efficacy towards *Mycobacterium* (MIC = 62.5 μg/mL). The remaining derivatives (**5a**, **5e**, **5h**, and **5x**) exhibited lower activity, with the lowest MIC recorded at 125 μg/mL (Table 1). Moreover, compounds demonstrating the highest potency with MIC values ≤ 15 μg/mL underwent a more comprehensive assessment of their cytotoxicity effects. This evaluation adhered precisely to international standards (ISO 10993-5:2009(E)), employing L929 cells and the MTT protocol (Bekier et al., 2021). The determination of the half maximal inhibitory concentration (IC_50_) values was carried out for 12 out of the 13 compounds tested, considering the occurrence of derivative precipitation in the growth medium for L929 cells (Table 1). With the exception of **5r**, the tested compounds showed low cytotoxicity at concentrations up to 10xMIC. For two compounds (**5g** and **5i**), the discriminatory index (IC_50_/MIC) was identified at the level of 20.

Within the synthesized quinoline compounds with phenyltriazole substituents, compound **5g** containing a 4-bromo substituent showed the strongest anti-tubercular effects against *M. tuberculosis* with a MIC value of 15 μg/mL. Moreover, compounds **4b**, **5c**, **5d**, and **5f** with 4-methoxyphenyl, 3-chlorophenyl, 3-bromophenyl, and 4-chlorophenyl substituents, respectively exhibited decreased activity with MIC values of 62.5 μg/mL, whereas, compounds **5a**, **5e**, and **5h** with unsubstituted phenyltriazole, 4-fluorophenyl, and 4-nitrophenyl showed even lower activity with MIC values of 125 μg/mL.

In the case of 6-chloroquinoline compounds with phenyltriazole substituents, compound **5n** with a 4-chlorophenyl substituent demonstrated the highest activity with a MIC of 12.5 μg/mL. Substituting this chlorophenyl with unsubstituted, 4-methoxy, 3-chloro, 3-bromo, and 4-bromo in compounds **5i**, **5j**, **5k**, **5L**, and **5o**, respectively, led to a slight decrease in activity with a MIC of 15 μg/mL. However, compounds **5m** and **5p** with 4-fluorophenyl and 4-nitrophenyl substituents showed a significant decrease in activity with a MIC of 62.5 μg/mL.

For 6-bromoquinoline compounds with phenyltriazole substituents, compounds **5q**, **5r**, **5s**, **5t**, **5v**, and **5w** containing unsubstituted phenyl, 4-methoxyphenyl, 3-chlorophenyl, 3-bromophenyl, 4-chlorophenyl, and 4-bromophenyl, respectively exhibited the most potent anti-tubercular effects with MIC values of 15 μg/mL. On the other hand, counterparts **5u** and **5x** with 4-fluorophenyl and 4-nitrophenyl showed reduced activity with MIC values of 62.5 and 125 μg/mL. Overall, compounds with substituted quinoline containing chloro and bromo groups displayed significant activity.

#### 2.2.2 Inhibitory effect toward MTB InhA

To assess the effectiveness of 4-carboxyquinoline-triazole hybrids **5g**, **5i**, and **5n**, which displayed notable cytotoxic effects and potent activity against MTB, further scrutiny was conducted to gauge their capacity to impede the InhA enzyme. In this analysis, INH served as a positive control. The outcomes, as displayed in Table 2, reveal that the examined derivatives effectively suppressed the InhA enzyme within the micromolar concentration ranges, recording IC_50_ values ranging from 0.72 ± 0.03 to 11.83 ± 0.49 µM. Remarkably, compound **5n**, exhibiting the best activity toward MTB with a MIC value of 12.5 μg/mL, also demonstrated substantial inhibition of the InhA enzyme (IC_50_ = 0.72 ± 0.03 µM), on par with that of INH (IC_50_ = 0.24 ± 0.01 µM). These results validated the inhibitory activity of compound **5n** against the InhA enzyme. The plausible binding mode for these analogues (**5g**, **5i**, and **5n**) in their interaction with InhA was explored through molecular docking, a discussion of which follows in the subsequent section.

**TABLE 2:** The IC_50_ values (µM) for the most effective counterparts (**5g**, **5i**, and **5n**) against InhA.

Compound	IC_50_ (µM)
**5g**	4.13 ± 0.17
**5i**	11.83 ± 0.49
**5n**	0.72 ± 0.03
**INH**	0.24 ± 0.01

### 2.3 *In silico* insights

#### 2.3.1 Molecular docking analysis

In order to enhance comprehension of the interaction mechanisms occurring at the binding site of a specific protein, molecular docking simulations were performed on compounds exhibiting strong *in vitro* effects towards the InhA enzyme of MTB. The docking results disclosed that 4-carboxyquinoline-triazole hybrids **5g**, **5i**, and **5n** exhibited binding affinities (ΔG_dock_) of −8.182, −9.337, and −9.493 kcal/mol, respectively. Notably, compound **5n** demonstrated the highest binding affinity within the active site of InhA, consistent with *in vitro* experimental findings.

**FIGURE 2:**
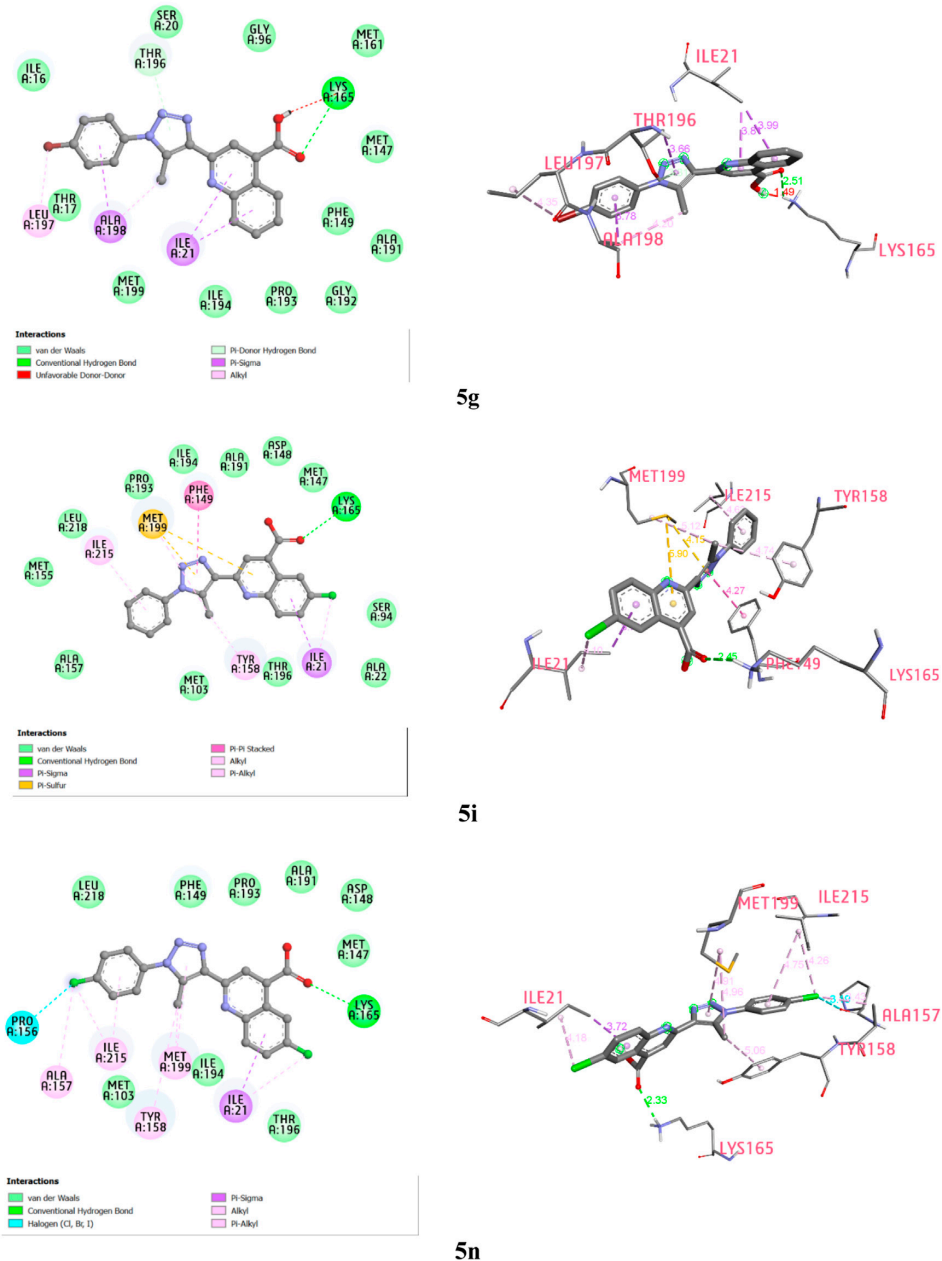
2D and 3D interactions of three compound **5g**, **5i**, and **5n** with InhA residues.

The 2D and 3D interactions of the three compounds (**5g**, **5i**, and **5n**) are detailed in Figure 2. These compounds formed hydrogen bonds between the oxygen atom of the carboxylic functionality in the molecule and the N-H of the amino acid residue Lys165. The hydrogen bond distances for these compounds with this residue were observed to be 2.51 Å, 2.45 Å, and 2.33 Å, respectively. Specifically, compound **5n** exhibited the shortest hydrogen bond distance, explaining its strongest affinity among the studied compounds due to the crucial role of hydrogen bonding in the protein-ligand complex. Furthermore, halogen bonding was identified in the InhA complex with this compound at residue Pro156. Various interactions, including pi-pi stacked, pi-sigma, alkyl, pi-alkyl, pi-sulfur, pi-donor hydrogen bonding, and van der Waals were observed in the binding site of InhA, contributing to the stability of the complex. Generally, the fused benzene ring of the quinoline scaffold in the studied compounds, and pi-sigma interactions with residue Ile21 were established.

In more detail, compound **5g** established alkyl interactions with residues Leu197 and Ala198, along with pi-sigma interactions with Ala198 and Ile21. Compound **5i** formed three pi-sulfur interactions with Met199 and pi-pi stacked interactions with Phe149. Pi-alkyl and alkyl interactions were also noticed in the InhA-**5i** complex with amino acid residues Ile215, Tyr158, and Ile21. This was also evident in the complexes InhA-**5n** and InhA-reference. Furthermore, compound **5n** exhibited additional pi-alkyl and alkyl interactions with other amino acid residues such as Met199 and Ala157.

#### 2.3.2 Molecular dynamics simulation

The best-docked pose with the most negative binding affinity of the top-lead compound **5n** obtained from the AutoDock Vina program was utilized as the input structure for a 100 ns MD simulation. The output result of the best docking was used to establish this procedure in a high-throughput fashion and scrutinize the dynamic binding modes of the ligand at the protein’s active site in explicit water conditions.

**FIGURE 3:**
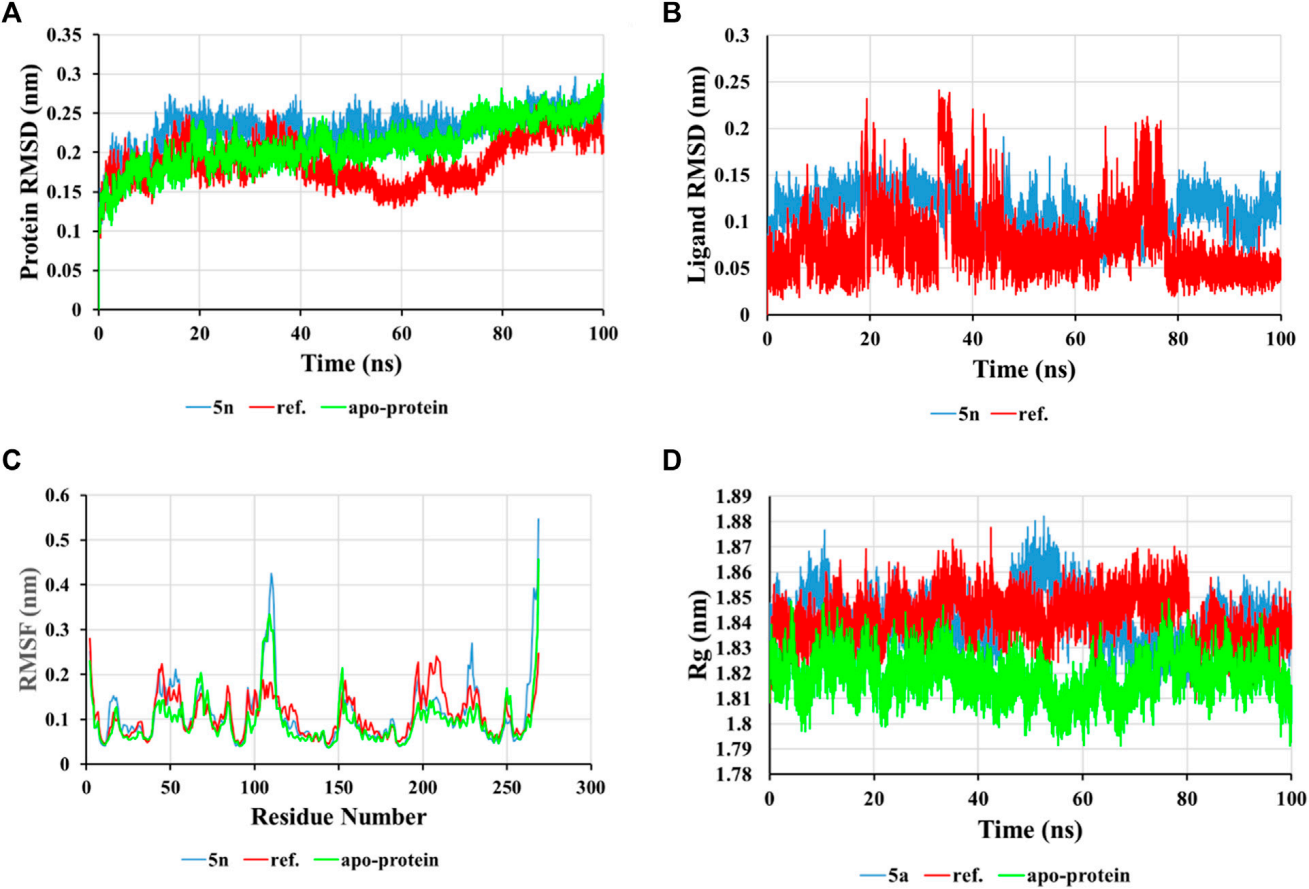
RMSD of protein backbone **(A)** and ligand **(B)** in solvated InhA-**5n** complex, RMSF values of the complex **(C)**, and Radius of gyration (Rg) of the complex **(D)** during 100 ns simulation time.

The examination of alterations in the conformation of the ligand-protein complex was evaluated by calculating the root-mean-square deviation (RMSD) from the original structure in order to assess the structural stability. The average RMSD value for the compound **5n** was determined to be approximately 0.229 nm, Figure 3A. From the protein RMSD chart, it can be observed that the structure undergoes slight changes, with the RMSD value increasing slightly over the first 20 ns and then stabilizing to form a well-defined complex throughout the simulation with an RMSD value around 2.3 Å. The accepted range for RMSD values is typically less than 3.0 Å, with lower RMSD values signifying enhanced stability within the system (Kufareva and Abagyan, 2012). In contrast, the ligand **5n** in the complex shows minor changes with an RMSD value less than 2.0 Å. Its average RMSD value is calculated as 0.11 nm, demonstrating the durability of the ligand within the active site of the InhA protein, which is illustrated in Figure 3B.

The root-mean-square fluctuation (RMSF) for the ligand-protein complex was graphed using the 100 ns MD trajectory to assess the mean fluctuation and flexibility of each specific amino acid (Figure 3C). The RMSF chart indicates the varying degrees of fluctuation observed in amino acid residues within the protein during the interaction state with the ligand over certain time intervals. This chart illustrates that the fluctuations in residues during interaction with compound 5n are greater compared to the residues of the apo-protein, particularly concentrated in the regions from 100 to 120 and 227 to 230. This observation implies that the compound 5n has a comparable stabilizing effect on this protein section.

The compactness of the InhA-**5n** complex was analyzed using a radius of gyration (Rg) chart. From the displayed results, a small change in Rg was observed at the 40–60 ns time point, followed by sustained stability for the ligand-protein complex during the simulation, with an average value of 1.841 nm, similar to the reference compound and apo-protein (Rg values of 1.841 and 1.819 nm, respectively), indicating the tightness of the structure (Figure 3D). In summary, the results indicate that the InhA-**5n** system remains stable throughout the 100 ns MD simulation under virtual physiological conditions.

#### 2.3.3 ADMET

The compound **5n**, with its molecular weight of 399.23 g/mol, 5 hydrogen bond acceptors, 1 hydrogen bond donor, molar refractivity of 104.04, and LogP of 3.71, is being investigated for its potential to inhibit InhA, and its drug-likeness and pharmacokinetics are currently under research. According to Supplementary Table S1, all these parameter values meet Lipinski’s proposed criteria (Lipinski et al., 1997). Additionally, the Absorption, Distribution, Metabolism, Excretion, and Toxicity (ADMET) profile of the synthesized compound **5n** has been preliminarily assessed to make pharmacokinetic predictions for potential drug candidates in clinical studies.

**FIGURE 4:**
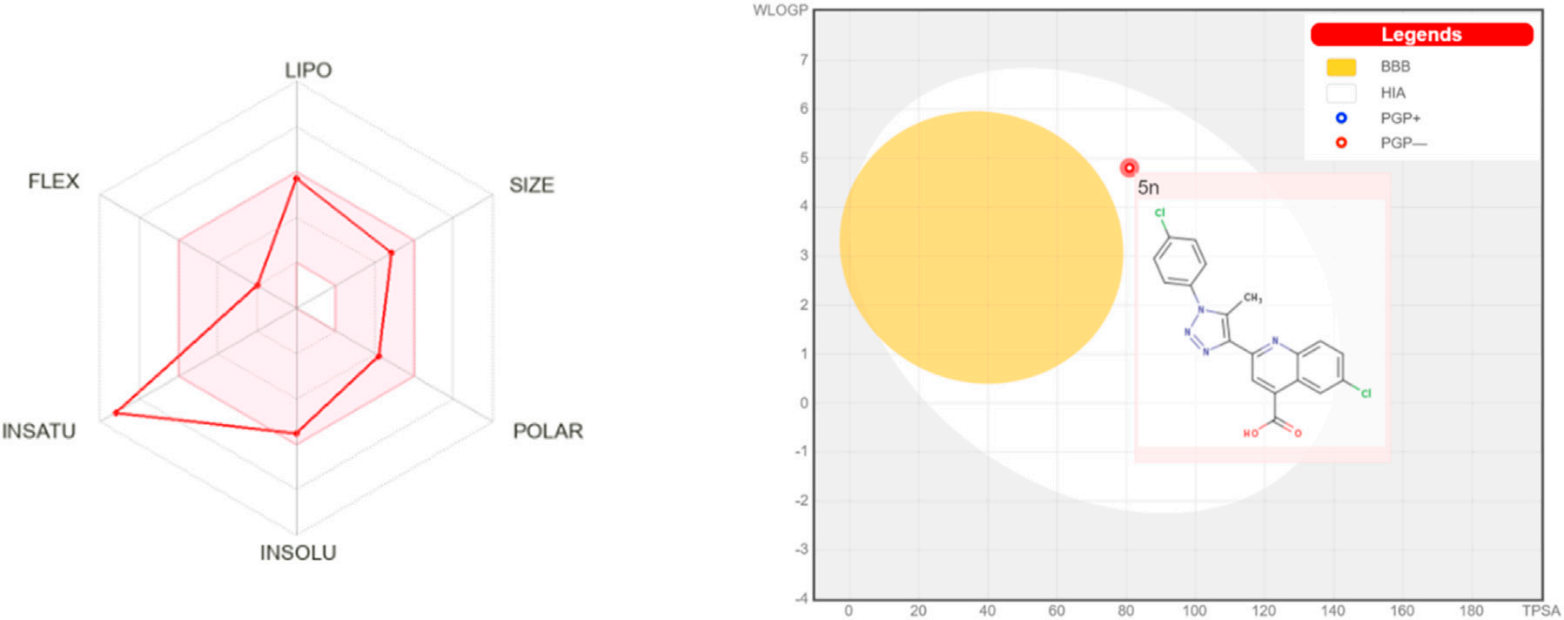
Swiss ADME bioavailability radar and BOILED-egg plot of compound **5n**.

As shown in Supplementary Table S2, ADMET profiling results indicate that compound **5n** has a high human intestinal absorption value of 94.021%. It exhibits high permeability through Caco-2 with a predicted value of 1.389 (>0.90). The skin permeation ability is high with a logKp value of −2.735 (<−2.5). CNS permeability study demonstrates good penetration (logPS value of −1.934 > −2), while the compound cannot cross the blood-brain barrier (logBB = −1.043 < −1) (Supplementary Table S2, Figure 4). Cytochrome P450 enzymes in the liver are generally not inhibited, except for cytochrome P2C9, a key enzyme in drug metabolism, which may be inhibited by this compound. The excretion is evaluated based on the total clearance, an important parameter in determining the dosing interval. Data indicates that the compound **5n** has a total clearance value of 0.192 log mL/min/kg. Toxicity parameters assessed in the ADMET profile of the new InhA inhibitor **5n** show inactivity with carcinogenicity, immunotoxicity, mutagenicity, cytotoxicity, no AMES toxicity, and no inhibition of hERG (I and II). However, it exhibits undesired hepatotoxicity. The toxicity level of 4, as indicated by the ProTox II web server, suggests the relative toxicity of compound **5n** (Banerjee et al., 2018a). Furthermore, the estimated Oral Rat Acute Toxicity (LD_50_) and Oral Rat Chronic Toxicity values for the synthesized compound **5n** are 2.822 mol/kg and 0.528 log mg/kg_bw/day, respectively.

## 3 Conclusion

In conclusion, the synthesized carboxyquinoline-triazole compounds **5a-x** in this investigation demonstrated promising potential as anti-tubercular agents, particularly against MTB. Despite not exhibiting significant activity against the highly resistant *M. abscessus*, all 24 compounds effectively suppressed the growth of *M. bovis BCG* and MTB. Among them, derivative **5n** emerged as the most potent, with a MIC value of 12.5 μg/mL against MTB, comparable to the standard drug INH. Moreover, the cytotoxicity assessment revealed low toxicity for most compounds, with compound **5n** demonstrating a favorable IC_50_-L929/MIC_*MTB*_ ratio of 16. Further investigation into the inhibition of the MTB InhA enzyme confirmed the efficacy of derivatives **5g**, **5i**, and **5n**, with compound **5n** displaying substantial inhibition with an IC_50_ value closely mirrored that of INH. Molecular docking simulations elucidated the binding affinities and interactions of these compounds within the active site of InhA, suggesting a comparable binding mode to reported InhA inhibitors and emphasizing the importance of hydrogen and halogen bonding, as well as various other interactions contributing to stability. Additionally, an MD analysis supported the stability of the InhA-**5n** complex over 100 ns, corroborating the binding mode observed in docking studies. ADMET profiling indicated favorable drug-like properties for compound **5n** that comply with Lipinski’s rule of five, including high intestinal absorption, permeability, and minimal toxicity, albeit with some potential for hepatotoxicity. Overall, compound **5n** stands out as a promising lead compound warranting further exploration as a potential anti-tubercular agent with favorable pharmacokinetic properties and low toxicity profile, offering a potential avenue for the development of novel compounds to combat tuberculosis and mitigate the challenge of resistance.

## 4 Experimental section

### 4.1 Chemistry

Melting points have been determined using the Electrothermal IA-9000 apparatus and have been reported without correction. The ^1^H NMR and ^13^C NMR spectra were recorded using Bruker Avance 500 MHz spectrometer (500 MHz ^1^H and 126 MHz ^13^C NMR). Deuterated dimethylsulfoxide (DMSO-*d*_6_) was used as a solvent in all samples. The progression of the reactions was observed through TLC using silica gel on aluminium sheets 60 F254 from Merck, with CHCl_3_/MeOH (9.5: 0.5 v/v) as the eluent, and iodine-potassium for visualization. It is important to mention that compounds **3a-h** had been synthesized previously (Dong et al., 2010; Singh et al., 2013; Bekheit et al., 2021).

#### 4.1.1 General procedures for preparing targeted 4-carboxyquinoline-triazole derivatives *(5a-x)*

Stirring a solution of isatin derivatives **4a-c** (1 mmol) and potassium hydroxide (2.5 mmol) in 5 mL of water was conducted at room temperature for 15–30 min. Following this, the reaction mixture received an addition of acetyl triazoles derivatives **3a-h** (1 mmol) and 10 mL of ethyl alcohol. Refluxing the reaction took place for a duration of 12 h, and subsequently, the mixture was acidified to achieve a pH of 2–3 using diluted HCl, resulting in the formation of a precipitate. This precipitate was then subjected to filtration, and underwent recrystallization after a water-washing process, ultimately yielding the pure target products 4-carboxyquinoline-triazole derivatives **5a-x**.

2-(5-Methyl-1-phenyl-1H-1,2,3-triazol-4-yl)benzo [b]pyridine-4-carboxylic acid (**5a**).

Orange powder; mp 278°C–280°C; yield (63%); ^1^H NMR (500 MHz, DMSO-d_6_) δ = 2.60 (s, 3H, triazole CH_3_), 6.86-6.88 (m, 1H, H-Arm), 7.01-7.04 (m, 1H, H-Arm), 7.45-7.47 (m, 1H, H-Arm), 7.53-7.56 (m, 1H, H-Arm), 7.59-7.65 (m, 6H, H3 of quinoline and H-Arm), 11.00 (brs, 1H, C(O)OH); ^13^C NMR (126 MHz, DMSO-d_6_) δ 10.23 (triazole CH_3_), 112.73, 118.35, 123.29, 125.20, 125.95 (2C), 130.27 (2C), 130.67, 135.54, 138.21, 138.90 (Arm), 143.38 (=C-N, triazole), 151.27 (C9 of quinoline), 159.88 (C2 of quinoline), 184.91 (C(O)OH); Analysis for C_19_H_14_N_4_O_2_, M.wt. (330.35 g/mol), Calcd.: % C, 69.08; H, 4.27; N, 16.96; Actual: % C, 68.96; H, 4.29; N, 17.03 (Pokhodylo et al., 2009).

2-(1-(4-Methoxyphenyl)-5-methyl-1H-1,2,3-triazol-4-yl)benzo[b]pyridine-4-carboxylic acid (**5b**).

Yellow powder; mp 222°C–224°C; yield (65%); ^1^H NMR (500 MHz, DMSO-d_6_) δ = 2.62 (s, 3H, triazole CH_3_), 3.85 (s, 3H, OCH_3_), 7.16 (d, 2H, J = 8.8 Hz, H-Arm), 7.50-7.56 (m, 5H, H-Arm), 7.58 (dd, 1H, J = 8.8 and 2.5 Hz, H-Arm), 8.73 (s, 1H, H3 of quinoline), 11.06 (s, 1H, C(O)OH); ^13^C NMR (126 MHz, DMSO-d_6_) = δ 9.67 (triazole CH_3_), 55.65 (OCH_3_), 112.25, 114.79 (2C), 117.80, 122.76, 124.67, 125.55, 126.92 (2C), 127.76, 134.60, 130.23, 137.74, 138.38 (Arm), 142.68 (=C-N, triazole), 150.79 (C9 of quinoline), 159.40 (C2 of quinoline), 167.38 (C(O)OH). Analysis for C_20_H_16_N_4_O_3_, M.wt. (360.37 g/mol), Calcd: % C, 66.66; H, 4.48; N, 15.55; Actual: % C, 66.78; H, 4.46; N, 15.49.

2-(1-(3-Chlorophenyl)-5-methyl-1H-1,2,3-triazol-4-yl)benzo [b]pyridine-4-carboxylic acid (**5c**).

Orange powder; mp 276°C–277°C; yield (51%); ^1^H NMR (500 MHz, DMSO-d_6_) δ = 2.59 (s, 3H, triazole CH_3_), 6.86 (d, 1H, *J* = 7.5 Hz, H-Arm), 7.00-7.03 (m, 1H, H-Arm), 7.44 (d, 1H, *J* = 7.5 Hz, H-Arm), 7.52-7.55 (m, 1H, H-Arm), 7.59 (d, 1H, *J* = 7.5 Hz, H-Arm), 7.63-7.69 (m, 3H, H-Arm), 7.97 (s, 1H, H3 of quinoline), 11.01 (brs, 1H, C(O)OH); ^13^C NMR (126 MHz, DMSO-d_6_) δ = 10.13 (triazole CH_3_), 112.74, 118.31, 123.27, 124.81, 125.17, 125.92, 130.71, 131.90, 134.48, 136.67, 138.51, 138.88 (Arm), 143.35 (=C-N, triazole), 151.27 (C9 of quinoline), 159.85 (C2 of quinoline), 185.06 (C(O)OH); Analysis for C_19_H_13_ClN_4_O_2_, M.wt. (364.79 g/mol), Calcd.: % C, 62.56; H, 3.59; N, 15.36; Actual: % C, 62.54; H, 3.60; N, 15.32.

2-(1-(3-Bromophenyl)-5-methyl-1H-1,2,3-triazol-4-yl)benzo [b]pyridine-4-carboxylic acid (**5d**).

Buff powder; mp 248°C–250°C; yield (78%); ^1^H NMR (500 MHz, DMSO-d_6_) δ = 2.60 (s, 3H, triazole CH_3_), 6.87 (d, 1H, *J* = 8.0 Hz, H-Arm), 7.01–7.04 (m, 1H, H-Arm), 7.45 (d, 1H, *J* = 7.5 Hz, H-Arm), 7.53–7.60 (m, 2H, H-Arm), 7.64-7.65 (m, 1H, H-Arm), 7.81 (d, 2H, *J* = 8.0 Hz, H-Arm), 7.90 (s, 1H, H3 of quinoline); 11.01 (brs, 1H, C(O)OH); ^13^C NMR (126 MHz, DMSO-D6) δ = 10.90 (triazole CH_3_), 114.37, 115.30, 119.62, 121.52, 124.62, 125.02, 127.34, 127.47, 128.89, 131.09, 131.93, 132.84, 135.31, 137.78(Arm), 141.91(=C-N, triazole), 152.50 (C9 of quinoline), 160.67(C2 of quinoline), 183.87(C(O)OH).Analysis for C_19_H_13_BrN_4_O_2_, M.wt. (409.24 g/mol), Calcd.: % C, 55.76; H, 3.20; N, 13.69; Actual: % C, 55.95; H, 3.19; N, 13.63.

2-(1-(4-Fluorophenyl)-5-methyl-1H-1,2,3-triazol-4-yl)benzo [b]pyridine-4-carboxylic acid (**5e**).

Buff powder; mp 290°C–291°C; yield (62%); ^1^H NMR (500 MHz, DMSO-d_6_) δ = 2.81 (s, 3H, triazole CH_3_), 7.49–7.52 (m, 2H, H-Arm), 7.67–7.70 (m, 1H, H-Arm), 7.75–7.84 (m, 3H, H-Arm), 8.09 (d, 1H, *J* = 8.0 Hz, H-Arm), 8.70-8.71 (m, 2H, H3 and H8 of quinoline), 13.98 (brs, 1H, C(O)OH); ^13^C NMR (126 MHz, DMSO-d_6_) δ = 11.01 (triazole CH_3_), 117.12, 117.33, 120.22, 123.90, 126.11, 128.37, 128.50, 128.56, 129.99, 130.80, 135.31, 137.41, 142.41 (=C-N, triazole), 148.75 (C9 of quinoline), 151.88 (C-F), 157.21 (C2 of quinoline), 167.88 (C(O)OH); Analysis for C_19_H_13_FN_4_O_2_, M.wt. (348.34 g/mol), Calcd.: % C, 65.51; H, 3.76; N, 16.08; Actual: % C, 65.56; H, 3.78; N, 16.04.

2-(1-(4-Chlorophenyl)-5-methyl-1H-1,2,3-triazol-4-yl)benzo [b]pyridine-4-carboxylic acid (**5f**).

Yellow powder; mp 246°C–248 °C; yield (63%); ^1^H NMR (500 MHz, DMSO-d_6_) δ = 2.60 (s, 3H, triazole CH_3_), 6.86 (d, 1H, *J* = 8.0 Hz, H-Arm), 7.01–7.10 (m, 1H, H-Arm), 7.45 (d, 1H, *J* = 7.5 Hz, H-Arm), 7.53–7.56 (m, 1H, H-Arm), 7.81–7.85 (m, 3H, H-Arm), 8.01 (d, 1H, *J* = 8.0 Hz, H-Arm), 8.7 (s, 1H, H3 of quinoline), 11.00 (brs, 1H, C(O)OH); ^13^C NMR (126 MHz, DMSO-d_6_) δ = 9.65 (triazole CH_3_), 112.19, 122.74, 124.65, 125.55, 127.21, 127.78, 129.77, 130.22, 133.79, 134.81, 137.89, 138.35 (Arm), 142.85 (=C-N, triazole), 150.70 (C9 of quinoline), 159.46 (C2 of quinoline), 167.33 (C(O)OH). Analysis for C_19_H_13_ClN_4_O_2_, M.wt. (364.79 g/mol), Calcd.: % C, 62.56; H, 3.59; N, 15.36; Actual: % C, 62.58; H, 3.58; N, 15.40 (Pokhodylo et al., 2009).

2-(1-(4-Bromophenyl)-5-methyl-1H-1,2,3-triazol-4-yl)benzo [b]pyridine-4-carboxylic acid (**5g**).

Yellow powder; mp 228°C–229°C; yield (58%); ^1^H NMR (500 MHz, DMSO-d_6_) δ = 2.61 (s, 3H, triazole CH_3_), 7.58–7.60 (m, 3H, H-Arm), 7.82–7.85 (m, 4H, H-Arm), 8.10 (d, 1H, *J* = 8.0 Hz, H-Arm), 8.71 (s, 1H, H3 of quinoline), 11.00 (brs, 1H, C(O)OH); ^13^C NMR (126 MHz, DMSO-d_6_) δ = 10.17 (triazole CH_3_), 123.34, 123.88, 125.18, 127.97, 130.92, 133.44, 134.78, 135.18, 136.53, 138.41, 138.66, 138.94 (Arm), 143.49 (=C-N, triazole), 154.63 (C9 of quinoline), 158.83 (C2 of quinoline), 194.22 (C(O)OH); Analysis for C_19_H_13_BrN_4_O_2_, M.wt. (409.24 g/mol), Calcd.: % C, 55.76; H, 3.20; N, 13.69; Actual: % C, 55.60; H, 3.21; N, 13.75.

2-(5-Methyl-1-(4-nitrophenyl)-1H-1,2,3-triazol-4-yl)benzo [b]pyridine-4-carboxylic acid (**5h**).

Yellow powder; mp 265°C–267°C; yield (67%); ^1^H NMR (500 MHz, DMSO-d_6_) δ = 2.62 (s, 3H, triazole CH_3_), 7.49–7.74 (m, 1H, H-Arm), 7.84–8.00 (m, 3H, H-Arm), 8.10–8.16 (m, 1H, H-Arm), 8.29 (d, 1H, *J* = 7.5 Hz, H-Arm), 8.44–8.52 (m, 2H, H-Arm), 8.72 (s, 1H, H3 of quinoline), 11.03 (brs, 1H, C(O)OH); ^13^C NMR (126 MHz, DMSO-d_6_) δ = 10.12 (triazole CH_3_), 119.87, 122.75, 123.37, 125.64, 127.05, 130.32, 130.39, 132.40, 137.59, 138.65, 138.89, 140.35 (Arm), 143.71 (=C-N, triazole), 148.53 (CH-NO_2_-CH), 157.95 (C2 of quinoline), 194.21 (C(O)OH); Analysis for C_19_H_13_N_5_O_4_, M.wt. (375.34 g/mol), Calcd.: % C, 60.80; H, 3.49; N, 18.66; Actual: % C, 60.81; H, 3.48; N, 18.69.

6-Chloro-2-(5-methyl-1-phenyl-1H-1,2,3-triazol-4-yl)benzo [b]pyridine-4-carboxylic acid (**5i**).

Yellow powder; mp > 300°C; yield (79%); ^1^H NMR (500 MHz, DMSO-d_6_) δ = 2.80 (s, 3H, triazole CH_3_), 7.61–7.69 (m, 5H, H-Arm), 7.82 (d, 1H, *J* = 9.0 Hz, H-Arm), 8.09 (d, 1H, *J* = 8.5 Hz, H-Arm), 8.79 (s, 1H, H3 of quinoline), 8.82 (brs, 1H, H5 of quinoline), 14.17 (brs, 1H, C(O)OH); ^13^C NMR (126 MHz, DMSO-d_6_) δ = 10.97 (triazole CH_3_), 121.56, 124.71, 125.05, 126.00, 130.27, 130.49, 131.19, 131.98, 132.93, 135.27, 135.99, 136.09, 142.16 (=C-N, triazole), 147.29 (C9 of quinoline), 152.40 (C2 of quinoline), 167.34 (C(O)OH); Analysis for C_19_H_13_ClN_4_O_2_, M.wt. (364.78 g/mol), Calcd.: % C, 62.56; H, 3.59; N, 15.36; Actual: % C, 62.67; H, 3.60; N, 15.30.

6-Chloro-2-(1-(4-methoxyphenyl)-5-methyl-1H-1,2,3-triazol-4-yl)benzo [b]pyridine-4-carboxylic acid (**5j**).

White powder; mp 287°C–289°C; yield (54%); ^1^H NMR (500 MHz, DMSO-d_6_) δ = 2.73 (s, 3H, triazole CH_3_), 3.83 (s, 3H, OCH_3_), 7.15 (d, 2H, *J* = 8.5 Hz, H-Arm), 7.56 (d, 2H, *J* = 8.5 Hz, H-Arm), 7.77 (d, 1H, *J* = 9.0 Hz, H-Arm), 8.01 (dd, 1H, *J* = 3.5 and 8.5 Hz, H7 of quinoline), 7.74 (d, 1H, *J* = 3.5 Hz, H5 of quinoline); 8.78 (s, 1H, H3 of quinoline); 11.11 (brs, 1H, C(O)OH); ^13^C NMR (126 MHz, DMSO-d_6_) δ = 10.90 (triazole CH_3_), 56.17 (OCH_3_), 114.37, 115.30, 119.62, 121.52, 124.62, 127.47, 131.09, 131.91, 132.84, 135.31, 137.78, 141.91 (=C-N, triazole), 149.77 (C9 of quinoline), 159.63 (C2 of quinoline), 160.67, 167.32 (C(O)OH); Analysis for C_20_H_15_ClN_4_O_3_, M.wt. (394.81 g/mol), Calcd.: % C, 60.84; H, 3.83; N, 14.19; Actual: % C, 60.90; H, 3.85; N, 14.15.

6-Chloro-2-(1-(3-chlorophenyl)-5-methyl-1H-1,2,3-triazol-4-yl)benzo [b]pyridine-4-carboxylic acid (**5k**).

Yellow powder; mp > 300°C; yield (62%); ^1^H NMR (500 MHz, DMSO-d_6_) δ = 2.79 (s, 3H, triazole CH_3_), 7.66-7.69 (m, 3H, H-Arm), 7.78 (d, 1H, *J* = 9.0 Hz, H-Arm), 7.84 (s, 1H, H-Arm), 8.03 (d, 1H, *J* = 9.0 Hz, H-Arm), 8.73 (s, 1H, H3 of quinoline); 8.78 (brs, 1H, H5 of quinoline); 14.10 (brs, 1H, C(O)OH); ^13^C NMR (126 MHz, DMSO-d_6_) δ = 10.97 (triazole CH_3_), 121.56, 124.71, 125.05, 126.00, 130.27, 130.49, 131.19, 131.98, 132.93, 135.27, 135.99, 136.09, 142.16 (=C-N, triazole), 147.29 (C9 of quinoline), 152.40 (C2 of quinoline), 167.34 (C(O)OH); Analysis for C_19_H_12_Cl_2_N_4_O_2_, M.wt. (399.23 g/mol), Calcd.: % C, 57.16; H, 3.03; N, 14.03; Actual: % C, 57.29; H, 3.03; N, 13.99.

2-(1-(3-Bromophenyl)-5-methyl-1H-1,2,3-triazol-4-yl)-6-chlorobenzo [b]pyridine-4-carboxylic acid (**5L**).

Buff powder; mp 248°C–250°C; yield (71%); ^1^H NMR (500 MHz, DMSO-d_6_) δ = 2.80 (s, 3H, triazole CH_3_), 7.55-7.63 (m, 1H, H-Arm), 7.71 (d, 1H, *J* = 9.0 Hz, H-Arm), 7.80–7.84 (m, 2H, H-Arm); 7.97–7.98 (m, 1H, H-Arm); 8.06 (d, 1H, *J* = 9.0 Hz, H-Arm), 8.76 (s, 1H, H3 of quinoline), 8.78 (d, 1H, *J* = 2.0 Hz, H-Arm), 14.10 (brs, 1H, C(O)OH); ^13^C NMR (126 MHz, DMSO-d_6_) δ = 10.86 (triazole CH_3_), 121.46, 122.64, 124.59, 124.90, 125.07, 125.15, 128.61, 131.04, 131.85, 132.11, 132.93, 133.63, 135.36, 137.20, 142.06 (=C-N, triazole), 147.07 (C9 of quinoline), 151.99 (C2 of quinoline), 167.23 (C(O)OH); Analysis for C_19_H_12_BrClN_4_O_2_, M.wt. (443.68 g/mol), Calcd.: % C, 51.43; H, 2.73; N, 12.63; Actual: % C, 51.25; H, 2.74; N, 12.67.

6-Chloro-2-(1-(4-fluorophenyl)-5-methyl-1H-1,2,3-triazol-4-yl)benzo [b]pyridine-4-carboxylic acid (**5m**).

Yellow powder; mp 298°C–299°C; yield (64%); ^1^H NMR (500 MHz, DMSO-d_6_) δ = 2.77 (s, 3H, triazole CH_3_), 7.48–7.51 (m, 2H, H-Arm), 7.74–7.76 (m, 2H, H-Arm), 7.79 (dd, 1H, *J* = 2.0 and 9.0 Hz, H-Arm), 8.04 (d, 1H, *J* = 9.0 Hz, H-Arm), 8.74 (s, 1H, H3 of quinoline), 8.79 (d, 1H, *J* = 2.0 Hz, H-Arm), 14.13 (brs, 1H, C(O)OH); ^13^C NMR (126 MHz, DMSO-d_6_) δ = 10.85 (triazole CH_3_), 117.10, 117.29, 121.48, 124.63, 124.96, 128.38, 128.45, 131.01, 131.84, 132.87, 135.39, 135.66, 142.01 (=C-N, triazole), 147.14 (C9 of quinoline), 152.19 (C2 of quinoline), 162.02 (C-F), 167.26 (C(O)OH); Analysis for C_19_H_12_ClFN_4_O_2_, M.wt. (382.77 g/mol), Calcd.: % C, 59.62; H, 3.16; N, 14.64; Actual: % C, 59.54; H, 3.17; N, 14.67.

6-Chloro-2-(1-(4-chlorophenyl)-5-methyl-1H-1,2,3-triazol-4-yl)benzo [b]pyridine-4-carboxylic acid (**5n**).

Pale yellow powder; mp > 300°C; yield (48%); ^1^H NMR (500 MHz, DMSO-d_6_) δ = 2.78 (s, 3H, triazole CH_3_), 7.72-7.73 (m, 4H, H-Arm), 7.79–7.82 (m, 1H, H-Arm), 8.04 (dd, 1H, *J* = 3.5 and 9.0 Hz, H-Arm), 8.74 (d, 1H, *J* = 3.5 Hz, H-Arm), 8.79 (brs, 1H, H3 of quinoline), 14.13 (brs, 1H, C(O)OH); ^13^C NMR (126 MHz, DMSO-d_6_) δ = 10.92 (triazole CH_3_), 121.51, 124.71, 125.04, 127.78, 130.31, 131.16, 131.96, 132.94, 134.90, 135.14, 135.43, 135.98, 142.22 (=C-N, triazole), 147.24 (C9 of quinoline), 152.24 (C2 of quinoline), 167.31 (C(O)OH); Analysis for C_19_H_12_Cl_2_N_4_O_2_, M.wt. (399.23 g/mol), Calcd.: % C, 57.16; H, 3.03; N, 14.03; Actual: % C, 57.13; H, 3.04; N, 14.01.

2-(1-(4-Bromophenyl)-5-methyl-1H-1,2,3-triazol-4-yl)-6-chlorobenzo [b]pyridine-4-carboxylic acid (**5o**).

White powder; mp > 300°C; yield (66%); ^1^H NMR (500 MHz, DMSO-d_6_) δ = 2.77 (s, 3H, triazole CH_3_), 7.64 (d, 2H, *J* = 8.5 Hz, H-Arm), 7.77 (dd, 1H, *J* = 2.0 and 9.0 Hz, H-Arm), 7.84 (d, 2H, *J* = 8.0 Hz, H-Arm), 8.02 (d, 1H, *J* = 8.5 Hz, H-Arm), 8.73 (brs, 1H, H3 of quinoline), 8.78 (d, 1H, *J* = 1.5 Hz, H-Arm), 14.10 (brs, 1H, C(O)OH), ^13^C NMR (126 MHz, DMSO-d_6_) δ = 10.91 (triazole CH_3_), 121.51, 123.66, 124.67, 125.00, 127.94, 131.08, 131.90, 132.93, 133.24, 135.30, 135.32, 135.78, 142.21 (=C-N, triazole), 147.18 (C9 of quinoline), 152.17 (C2 of quinoline), 167.26 (C(O)OH). Analysis for C_19_H_12_BrClN_4_O_2_, M.wt. (443.68 g/mol), Calcd.: % C, 51.43; H, 2.73; N, 12.63; Actual: % C, 51.45; H, 2.73; N, 12.60.

6-Chloro-2-(5-methyl-1-(4-nitrophenyl)-1H-1,2,3-triazol-4-yl)benzo [b]pyridine-4-carboxylic acid (**5p**).

Orange powder; mp 235°C–237°C; yield (47%); ^1^H NMR (500 MHz, DMSO-d_6_) δ = 2.90 (s, 3H, triazole CH_3_), 6.88-6.90 (m, 3H, H-Arm), 7.52 (s, 1H, H-Arm), 7.57-7.59 (m, 1H, H-Arm), 7.86-8.16 (m, 2H, H-Arm), 8.44-8.50 (m, 1H, H-Arm), 11.10 (brs, 1H, C(O)OH); Analysis for C_19_H_12_ClN_5_O_4_, M.wt. (409.78 g/mol), Calcd.: % C, 55.69; H, 2.95; N, 17.09; Actual: % C, 55.79; H, 2.94; N, 17.04.

6-Bromo-2-(5-methyl-1-phenyl-1H-1,2,3-triazol-4-yl)benzo [b]pyridine-4-carboxylic acid (**5q**).

Yellow powder; mp > 300°C; yield (74%); ^1^H NMR (500 MHz, DMSO-d_6_) δ = 2.77 (s, 3H, triazole CH_3_), 7.60-7.68 (m, 5H, H-Arm), 7.89 (dd, 1H, *J* = 2.0 and 9.0 Hz, H-Arm), 7.95 (d, 1H, *J* = 9.0 Hz, H-Arm), 8.74 (s, 1H, H3 of quinoline), 8.95 (s, 1H, H5 of quinoline), 14.11 (brs, 1H, C(O)OH); ^13^C NMR (126 MHz, DMSO-d_6_) δ = 11.39 (triazole CH_3_), 121.49, 121.67, 125.12, 125.97, 128.26, 130.28, 130.46, 131.98, 133.64, 135.22, 135.67, 136.08, 142.13 (=C-N, triazole), 147.36 (C9 of quinoline), 152.40 (C2 of quinoline), 167.29 (C(O)OH); Analysis for C_19_H_13_BrN_4_O_2_, M.wt. (409.24 g/mol), Calcd.: % C, 55.76; H, 3.20; N, 13.69; Actual: % C, 55.69; H, 3.21; N, 13.66.

6-Bromo-2-(1-(4-methoxyphenyl)-5-methyl-1H-1,2,3-triazol-4-yl)benzo [b]pyridine-4-carboxylic acid (**5r**).

Yellow powder; mp 294°C–295°C; yield (53%); ^1^H NMR (500 MHz, DMSO-d_6_) δ = 2.74 (s, 3H, triazole CH_3_), 3.84 (s, 3H, OCH_3_), 7.15 (d, 2H, *J* = 9.5 Hz, H-Arm), 7.57 (d, 2H, *J* = 9.0 Hz, H-Arm), 7.90 (dd, 1H, *J* = 2.5 and 9.5 Hz, H-Arm), 7.97 (d, 1H, *J* = 9.5 Hz, H-Arm); 8.75 (s, 1H, H3 of quinoline), 8.96 (d, 1H, *J* = 2.5 Hz, H5 of quinoline), 14.13 (s, 1H, C(O)OH); ^13^C NMR (126 MHz, DMSO-d_6_) *δ* = 10.90 (triazole CH_3_), 56.17 (OCH_3_), 115.30, 121.48, 125.10, 127.46, 128.23, 128.90, 131.95, 133.63, 135.07, 135.31, 135.67, 141.89 (=C-N, triazole), 147.38 (C9 of quinoline), 152.49 (C2 of quinoline), 160.67, 167.29 (C(O)OH); Analysis for C_20_H_15_BrN_4_O_3_, M.wt. (439.26 g/mol), Calcd.: % C, 54.69; H, 3.44; N, 12.75; Actual: % C, 54.76; H, 3.43; N, 12.70.

6-Bromo-2-(1-(3-chlorophenyl)-5-methyl-1H-1,2,3-triazol-4-yl)benzo [b]pyridine-4-carboxylic acid (**5s**).

Yellow powder; mp > 300°C; yield (67%); ^1^H NMR (500 MHz, DMSO-d_6_) δ = 2.79 (s, 3H, triazole CH_3_), 7.63-7.71 (m, 3H, H-Arm), 7.84-7.85 (m, 1H, H-Arm), 7.89 (dd, 1H, *J* = 2.0 and 9.0 Hz, H-Arm), 7.96 (d, 1H, *J* = 9.0 Hz, H-Arm), 8.73 (s, 1H, H3 of quinoline); 8.95 (s, 1H, H5 of quinoline), 14.14 (brs, 1H, C(O)OH); ^13^C NMR (126 MHz, DMSO-d_6_) δ = 10.90 (triazole CH_3_), 121.45, 124.79, 125.92, 128.22, 130.51, 131.89, 131.97, 133.67, 134.48, 135.48, 135.71, 137.20, 142.18 (=C-N, triazole), 147.34 (C9 of quinoline), 152.21 (C2 of quinoline), 167.25 (C(O)OH); Analysis for C_19_H_12_BrClN_4_O_2_, M.wt. (443.69 g/mol), Calcd.: % C, 51.43; H, 2.73; N, 12.63; Actual: % C, 51.57; H, 2.74; N, 12.58.

6-Bromo-2-(1-(3-bromophenyl)-5-methyl-1H-1,2,3-triazol-4-yl)benzo [b]pyridine-4-carboxylic acid (**5t**).

Yellow powder; mp 299°C–300°C; yield (71%); ^1^H NMR (500 MHz, DMSO-d_6_) δ = 2.80 (s, 3H, triazole CH_3_), 7.59-7.64 (m, 1H, H-Arm); 7.72-7.74 (m, 1H, H-Arm), 7.83-7.85 (m, 1H, H-Arm), 7.92-7.94 (m, 1H, H-Arm), 7.97-8.02 (m, 2H, H3 of quinoline and H-Arm), 8.75 (d, 1H, *J* = 5.0 Hz, H-Arm), 8.97 (d, 1H, *J* = 3.0 Hz, H5 of quinoline), 14.19 (brs, 1H, C(O)OH); ^13^C NMR (126 MHz, DMSO-d_6_) δ = 10.91 (triazole CH_3_), 121.46, 121.70, 122.65, 125.15, 128.22, 128.69, 131.95, 132.10, 133.41, 133.64, 135.47, 135.64, 137.29, 142.15 (=C-N, triazole), 147.32 (C9 of quinoline), 152.20 (C2 of quinoline), 167.25 (C(O)OH); Analysis for C_19_H_12_Br_2_N_4_O_2_, M.wt. (488.14 g/mol), Calcd.: % C, 46.75; H, 2.48; N, 11.48; Actual: % C, 46.86; H, 2.47; N, 11.45.

6-Bromo-2-(1-(4-fluorophenyl)-5-methyl-1H-1,2,3-triazol-4-yl)benzo [b]pyridine-4-carboxylic acid (**5u**).

Yellow powder; mp > 300°C; yield (42%); ^1^H NMR (500 MHz, DMSO-d_6_) δ = 2.77 (s, 3H, triazole CH_3_), 7.45-7.52 (m, 2H, H-Arm), 7.66-7.70 (m, 1H, H-Arm), 7.74-7.76 (m, 1H, H-Arm), 7.90 (dd, 1H, *J* = 2.0 and 9.0 Hz, H-Arm), 7.97 (d, 1H, *J* = 9.0 Hz, H-Arm), 8.74 (s, 1H, H3 of quinoline), 8.96 (d, 1H, *J* = 2.5 Hz, H5 of quinoline), 14.13 (s, 1H, C(O)OH); ^13^C NMR (126 MHz, DMSO-d_6_) δ = 10.68 (triazole CH_3_), 114.79, 115.03, 117.04, 117.23, 119.62, 121.21, 121.62, 127.33, 128.19, 131.68, 132.19, 135.21, 140.70, 141.87 (=C-N, triazole), 146.98 (C9 of quinoline), 151.82 (C2 of quinoline), 167.01 (C(O)OH); Analysis for C_19_H_12_BrFN_4_O_2_, M.wt. (427.22 g/mol), Calcd.: % C, 53.42; H, 2.83; N, 13.11; Actual: % C, 53.43; H, 2.84; N, 13.08.

6-Bromo-2-(1-(4-chlorophenyl)-5-methyl-1H-1,2,3-triazol-4-yl)benzo [b]pyridine-4-carboxylic acid (**5v**).

Yellow powder; mp > 300°C; yield (57%); ^1^H NMR (500 MHz, DMSO-d_6_) δ = 2.78 (s, 3H, triazole CH_3_), 7.65-7.73 (m, 4H, H-Arm); 7.89-7.92 (m, 1H, H-Arm), 7.97 (dd, 1H, *J* = 3.5 and 9.0 Hz, H-Arm), 8.73 (d, 1H, *J* = 4.0 Hz, H-Arm), 8.96 (d, 1H, *J* = 1.5 Hz, H5 of quinoline), 14.15 (brs, 1H, C(O)OH); ^13^C NMR (126 MHz, DMSO-d_6_) δ = 10.92 (triazole CH_3_), 121.45, 121.67, 125.12, 127.72, 128.22, 130.29, 131.94, 133.63, 134.88, 135.12, 135.38, 135.67, 142.20 (=C-N, triazole), 147.32 (C9 of quinoline), 152.24 (C2 of quinoline), 167.26 (C(O)OH); Analysis for C_19_H_12_BrClN_4_O_2_, M.wt. (443.69 g/mol), Calcd.: % C, 51.43; H, 2.73; N, 12.63; Actual: % C, 51.61; H, 2.72; N, 12.59.

6-Bromo-2-(1-(4-bromophenyl)-5-methyl-1H-1,2,3-triazol-4-yl)benzo [b]pyridine-4-carboxylic acid (**5w**).

Yellow powder; mp > 300°C, yield (60%); ^1^H NMR (500 MHz, DMSO-d_6_) δ = 2.77 (s, 3H, triazole CH_3_), 7.64 (d, 2H, *J* = 8.5 Hz, H-Arm), 7.84 (d, 2H, *J* = 8.5 Hz, H-Arm), 7.88 (dd, 1H, *J* = 2.0 and 9.0 Hz, H-Arm), 7.95 (d, 1H, *J* = 9.0 Hz, H-Arm), 8.72 (s, 1H, H3 of quinoline), 8.94 (d, 1H, *J* = 2.0 Hz, H5 of quinoline), 14.12 (s, 1H, C(O)OH); ^13^C NMR (126 MHz, DMSO-d_6_) δ = 10.93 (triazole CH_3_), 121.46, 121.68, 123.65, 125.12, 127.93, 128.22, 131.95, 133.24, 133.64, 135.29, 135.35, 135.67, 142.22 (=C-N, triazole), 147.33 (C9 of quinoline), 152.23 (C2 of quinoline), 167.25 (C(O)OH); Analysis for C_19_H_12_Br_2_N_4_O_2_, M.wt. (488.14 g/mol), Calcd.: % C, 46.75; H, 2.48; N, 11.48; Actual: % C, 46.56; H, 2.49; N, 11.51.

6-Bromo-2-(5-methyl-1-(4-nitrophenyl)-1H-1,2,3-triazol-4-yl)benzo [b]pyridine-4-carboxylic acid (**5x**).

Red powder; mp 236°C–238°C; yield (71%); ^1^H NMR (500 MHz, DMSO-d_6_) δ = 2.86 (s, 3H, triazole CH_3_), 6.82 (d, 1H, *J* = 8.0 Hz, H-Arm), 7.58 (s, 1H, H-Arm), 7.67 (d, 1H, *J* = 8.5 Hz, H-Arm), 7.94-8.02 (m, 3H, H-Arm), 8.45 (d, 1H, *J* = 8.5 Hz, H-Arm), 8.72 (s, 1H, H3 of quinoline), 11.13 (s, 1H, C(O)OH); ^13^C NMR (126 MHz, DMSO-d_6_) δ = 11.10 (triazole CH_3_), 114.83, 120.03, 121.84, 125.63, 126.90, 127.02, 127.39, 128.27, 132.05, 133.78, 135.68, 135.87, 140.58, 150.14 (C-NO_2_), 159.44 (C2 of quinoline), 167.24 (C(O)OH); Analysis for C_19_H_12_BrN_5_O_4_, M.wt. (454.24 g/mol), Calcd.: % C, 50.24; H, 2.66; N, 15.42; Actual: % C, 50.27; H, 2.65; N, 15.47.

### 4.2 Biological evaluations

The experimental procedures for the antimycobacterial (Franzblau et al., 1998) and MTB InhA inhibition (He et al., 2007) biological experiments were conducted using the reported protocols (Supplementary Material).

### 4.3 *In silico* studies

In silico studies involved molecular docking and molecular dynamics (MD) simulations using AutoDock Vina v1.2.3 (Eberhardt et al., 2021) and GROMACS v2023 (Van Der Spoel et al., 2005), respectively. The methods employed for the preparation of the tested compounds and InhA protein (PDB ID: 4TZK (He et al., 2006)) in both simulations and all the subsequent steps are elaborated in detail in the Supplementary Material section. Additionally, drug-likeness analyses and ADMET predictions were made using SwissADME (Daina et al., 2017) and pkCSM (Pires et al., 2015), and toxicity assessments via ProTox II (Banerjee et al., 2018b), following established protocols for thorough evaluation (Supplementary Material).

## Data Availability

The original contributions presented in the study are included in the article/supplementary materials; further inquiries can be directed to the corresponding authors.
