# Supplementary material for: Identification of 2-(N-aryl-1,2,3-triazol-4-yl) quinoline derivatives as antitubercular agents endowed with InhA inhibitory activity
Source: Front Chem. 2024 Aug 7;12:1424017. doi: 10.3389/fchem.2024.1424017 (PMC11337105; doi:10.3389/fchem.2024.1424017)
Supplement: Supplementary file 1 [file DataSheet1.PDF]

Supporting Materials

Ahmed Sabt[1], Maha-Hamadien Abdulla[2,*], Manal S. Ebaid [1,3], Jakub Pawełczyk[4], Hayam A. Abd El Salam[5], Ninh The Son[6,7], Nguyen Xuan Ha[8], Mansoor-Ali Vaali Mohammed[2], Thamer Traiki[2], Ahmed E. Elsawi [9], Bozena Dziadek[10], Jaroslaw Dziadek[4,*], Wagdy M. Eldehna[9,11*]

[1] Chemistry of Natural Compounds Department, Pharmaceutical and Drug Industries Research Institute, National Research Center, Dokki, Cairo 12622, Egypt
[2] Colorectal Research Chair, Department of Surgery, College of Medicine, King Saud University, Riyadh, Saudi Arabia
[3] Department of Chemistry, College of Science, Northern Border University, Arar, Saudi Arabia
[4]Laboratory of Genetics and Physiology of Mycobacterium, Institute of Medical Biology of the Polish Academy of Sciences, Lodz, Poland
[5] Department of Green Chemistry, National Research Center, Dokki, Cairo 12622, Egypt
[6] Institute of Chemistry, Vietnam Academy of Science and Technology (VAST), 18 Hoang Quoc Viet, Caugiay, Hanoi 10000, Vietnam
[7] Department of Chemistry, Graduate University of Science and Technology, VAST, 18 Hoang Quoc Viet, Cau Giay, Hanoi 10000, Vietnam
[8] Institute of Natural Products Chemistry, VAST, 18 Hoang Quoc Viet, Caugiay, Hanoi 10000, Vietnam
[9] Department of Pharmaceutical Chemistry, Faculty of Pharmacy, Kafrelsheikh University, Kafrelsheikh, P.O. Box 33516, Egypt
[10] Department of Molecular Microbiology, Faculty of Biology and Environmental Protection, University of Lodz, Lodz, Poland
[11] Department of Pharmaceutical Chemistry, Faculty of Pharmacy, Pharos University, Alexandria 21521, Egypt

Table S1. Physicochemical Properties of the compound **5n**

No.	Physicochemical Properties	Predicted value
1	Formula	$C_{19}H_{12}Cl_2N_4O_2$
2	Molecular weight	399.23 g/mol
3	Num. heavy atoms	27
4	Num. arom. heavy atoms	21
5	Num. rotatable bonds	3
6	Num. H-bond acceptors	5
7	Num. H-bond donors	1
8	Molar Refractivity	104.04
9	TPSA	80.90 Å^2
10	$LogP_{o/w}$	3.71

Table S2. ADMET properties of the compound **5n**

Property	Model name	Predicted value	Unit
**Absorption**	Water solubility	-2.894	Numeric (log mol/L)
	Caco2 permeability	1.389	Numeric
	Intestinal absorption (human)	94.021	Numeric (% Absorbed)
	Skin Permeability	-2.735	Numeric (log Kp)
	P-glycoprotein substrate	No	Categorical (Yes/No)
	P-glycoprotein I inhibitor	No	Categorical (Yes/No)
	P-glycoprotein II inhibitor	Yes	Categorical (Yes/No)
**Distribution**	VDss (human)	-0.949	Numeric (log L/kg)
	Fraction unbound (human)	0.212	Numeric (Fu)
	BBB permeability	-1.043	Numeric (log BB)
	CNS permeability	-1.934	Numeric (log PS)
	CYP2D6 substrate	No	Categorical (Yes/No)
	CYP3A4 substrate	No	Categorical (Yes/No)
	CYP1A2 inhibitor	No	Categorical (Yes/No)
	CYP2C19 inhibitor	No	Categorical (Yes/No)
	CYP2C9 inhibitor	Yes	Categorical (Yes/No)
	CYP2D6 inhibitor	No	Categorical (Yes/No)
	CYP3A4 inhibitor	No	Categorical (Yes/No)
**Excretion**	Total Clearance	0.192	Numeric (log ml/min/kg)
	Renal OCT2 substrate	No	Categorical (Yes/No)
**Toxicity**	AMES toxicity	No	Categorical (Yes/No)
	Max. tolerated dose (human)	0.793	Numeric
	hERG I inhibitor	No	Categorical (Yes/No)
	hERG II inhibitor	No	Categorical (Yes/No)
	Oral Rat Acute Toxicity (LD_{50})	2.822	Numeric (mol/kg)
	Oral Rat Chronic Toxicity (LOAEL)	0.528	Numeric (log mg/kg_bw/day)
	Hepatotoxicity	Yes	Categorical (Yes/No)
	Skin Sensitisation	No	
	T.Pyriformis toxicity	0.285	Numeric (log ug/L)
	Minnow toxicity	-1.248	Numeric (log mM)
	Toxicity class	4	
	Carcinogenicity	inactive	Categorical (active/inactive)
	Immunotoxicity	inactive	Categorical (active/inactive)
	Mutagenicity	inactive	Categorical (active/inactive)
	Cytotoxicity	inactive	Categorical (active/inactive)

3. Biological evaluations

3.1. Antitubercular activity

The MIC for *M. tuberculosis* $H_{37}Rv$, *M. bovis* BCG, and *M. abscessus* was determined in liquid 7H9/OADC (Middlebrook, Difco, Baltimore, MD, United States) media supplemented with various concentrations of the tested chemical agents. The carboxy quinoline triazole were dissolved in dimethyl sulfoxide (DMSO) and added directly to the growth medium. The final concentration of DMSO in the medium never exceeded 0.1% (vol/vol), and DMSO did not affect the growth of the bacilli. To define the MIC value, the MABA test was applied as described by Franzblau *et al* [1]. The susceptibility of the tested strains was assessed based on the change in color from blue to pink based on visual inspection. Wells containing only bacteria, medium, or compound were used as controls in this experiment, and the MABA test was repeated independently three times.

3.2. Evaluation of InhA inhibition

Screening of InhA Inhibitors was carried out using the endpoint assay, IC_{50} values were determined in 96-well plates by serial dilutions with DMSO of each inhibitor. The final concentration of DMSO in the final assay was 1%. The IC_{50} values were determined using 5 concentrations, under saturating substrate conditions. The concentrations of other components in the assay were as follows: NADH 250 μM (stock 1 mM, 50 μL, Km 7.6 μM); InhA 20 nM (stock 80 nM, 50 μL). OCoA 500 μM (stock 2 mM, 50 μL, Km 467 μM) was prepared freshly by dissolving the flaky solid substrate immediately before the assay; the remaining material was kept at -80° C and was used up within two days. The reaction was monitored over 10 min at room temperature. IC_{50} values were calculated from plots of enzyme activity versus the log of the inhibitor concentration using the GRAFIT-IC50-4 parameter fit software (Grafit 4.021, Erithacus) [2].

3.3. Molecular docking

In this section, AutoDock Vina v1.2.3 is employed for the entire molecular docking simulation [3]. The three-dimensional crystal structure of the InhA protein complexed with (*3S*)-1-cyclohexyl-*N*-(3,5-dichlorophenyl)-5-oxopyrrolidine-3-carboxamide (as reference inhibitor) is prepared by accessing the RCSB PDB website with entry 4TZK

(https://www.rcsb.org/structure/4TZK), then downloaded in *.pdb file format [4]. The structure coordinate file, after retrieval, is input into the PyMOL software to eliminate water and other unnecessary molecules for the docking simulation. Subsequently, hydrogen atoms are added, the partial charges are computed using the Kollman method, and the file is saved in *.pdbqt format using AutoDockTools software. The synthetic molecules (**5g**, **5i**, and **5n**) and the co-crystallized ligand are drawn using Marvin JS software and geometrically optimized with MMFF94s force field using the OpenBabel program [5, 6]. To validate the docking protocol and obtain the binding affinity of the co-crystallized ligand, re-docking is performed. According to the validation method documented, the success scoring function is defined as the function in which the RMSD of the pose of the re-docked co-crystallize ligand is less than 2 Å compared to the experimental pose. The RMSD value is calculated using the DockRMSD web server. The grid box is prepared using AutoDockTools v1.5.6 software with dimensions set at 25Åx25Åx25Å. The grid center is positioned based on the center of the co-crystallized ligand. Other parameters are set to default values according to the AutoDock Vina program, except for the exhaustiveness value, which is set to 400. The docking results of the three synthetic compounds and the co-crystallized ligand are visualized in 2D and 3D interaction plots using Discovery Studio Visualizer software.

3.4. Molecular dynamics

Molecular dynamics (MD) simulations were conducted over a period of 100 ns on the most potential compound **5n** and the reference molecule in complex with *Mycobacterium tuberculosis* InhA using the GROMACS v2023 platform [7]. The protein topology was established using the 'gmx pdb2gmx' command in the GROMACS program with the CHARMM27 force field. Meanwhile, the ligand topology and parameter files were obtained from the SwissParam web server [8, 9]. Subsequently, the ligand and protein structure files were combined into a complex system and solvated with the TIP3P water model in a triclinic box. Due to the system's unbalanced charge, counterions were added to the simulation box to neutralize the system. Energy minimization was performed using the steepest descent algorithm until the system reached a force of less than 1000 kJ/mol. The NVT and NPT ensembles were used to equilibrate complex systems (4TZK-5n and 4TZK-1VV) every 2 ns. The V-rescale and C-rescale algorithms were applied to maintain the temperature and pressure at 300K and 1 bar, respectively. Additionally, MD

simulation parameters included a timestep of 2 fs, long-range electrostatic, and van der Waals interactions with a cut-off value of 1.2 nm. The LINCS algorithm was used to constrain bond lengths. After removing all restraints, MD production runs continued from the NPT ensemble for a set simulation time of 100 ns. RMSD, RMSF, and Rg graphs from the MD simulation trajectories were plotted using Excel software.

3.5. *In silico* drug-likeness and ADMET predictions

Drug-likeness analyses were conducted based on Lipinski's rule, and the parameters (molecular weight: MW, MLogP, number of hydrogen acceptors: HA, and number of hydrogen donors: HD) were calculated using the SwissADME web server [10]. To predict pharmacokinetic parameters related to the processes of absorption, distribution, metabolism, and excretion (ADME), the chemical SMILES representation of compounds was submitted to the pkCSM web server [11]. Subsequently, toxicity parameters, including toxicity level, LD_{50} values, and other data such as hepatoxicity, carcinogenicity, immunotoxicity, mutagenicity, and cytotoxicity, were assessed using the ProTox II web server [12].

References

1. Franzblau SG, Witzig RS, McLaughlin JC, Torres P, Madico G, Hernandez A, DegnanMT, Cook MB, Quenzer VK, Ferguson RM, Gilman RH. Rapid, low-technology MIC determination with clinical Mycobacterium tuberculosis isolates by using the microplate Alamar Blue assay. J Clin Microbiol. 1998 Feb;36(2):362-6.
2. He X, Alian A, Ortiz de Montellano PR. Inhibition of the Mycobacterium tuberculosis enoyl acyl carrier protein reductase InhA by arylamides. Bioorg Med Chem. 2007 Nov 1;15(21):6649-58. doi: 10.1016/j.bmc.2007.08.013. Epub 2007 Aug 15. PMID: 17723305; PMCID: PMC2020492.
3. Eberhardt, J., Santos-Martins, D., Tillack, A. F., & Forli, S. (2021). AutoDock Vina 1.2. 0: New docking methods, expanded force field, and python bindings. *Journal of chemical information and modeling*, *61*(8), 3891-3898
4. He, X., Alian, A., Stroud, R., & Ortiz de Montellano, P. R. (2006). Pyrrolidine carboxamides as a novel class of inhibitors of enoyl acyl carrier protein reductase from Mycobacterium tuberculosis. *Journal of medicinal chemistry*, *49*(21), 6308-6323
5. Halgren, T. A. (1999). MMFF VI. MMFF94s option for energy minimization studies. *Journal of computational chemistry*, *20*(7), 720-729.
6. O'Boyle, N. M., Banck, M., James, C. A., Morley, C., Vandermeersch, T., & Hutchison, G. R. (2011). Open Babel: An open chemical toolbox. *Journal of cheminformatics*, *3*(1), 1-14.
7. Van Der Spoel, D., Lindahl, E., Hess, B., Groenhof, G., Mark, A. E., & Berendsen, H. J. (2005). GROMACS: fast, flexible, and free. *Journal of computational chemistry*, *26*(16), 1701-1718.
8. Bugnon M, Goullieux M, Röhrig UF, Perez MAS, Daina A, Michielin O, Zoete V. SwissParam 2023: a modern web-based tool for efficient small molecule parameterization. *J. Chem. Inf. Model.*, **2023**, *63(21)*, 6469-75.
9. Yesselman JD, Price DJ, Knight JL, Brooks CL 3rd. MATCH: An atom-typing toolset for molecular mechanics force fields. *J. Comput. Chem.,* **2011**, *33(2)*, 189-202.
10. Daina, A., Michielin, O., & Zoete, V. (2017). SwissADME: a free web tool to evaluate pharmacokinetics, drug-likeness and medicinal chemistry friendliness of small molecules. *Scientific reports*, *7*(1), 42717
11. Pires, D. E., Blundell, T. L., & Ascher, D. B. (2015). pkCSM: predicting small-molecule pharmacokinetic and toxicity properties using graph-based signatures. *Journal of medicinal chemistry*, *58*(9), 4066-4072
12. Banerjee, P., Eckert, A. O., Schrey, A. K., & Preissner, R. (2018). ProTox-II: a webserver for the prediction of toxicity of chemicals. *Nucleic acids research*, *46*(W1), W257-W263

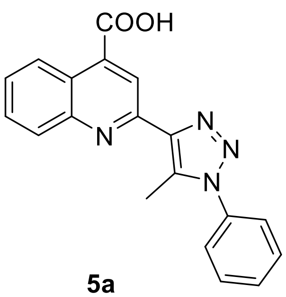
COOH
N
N
N
N

5a

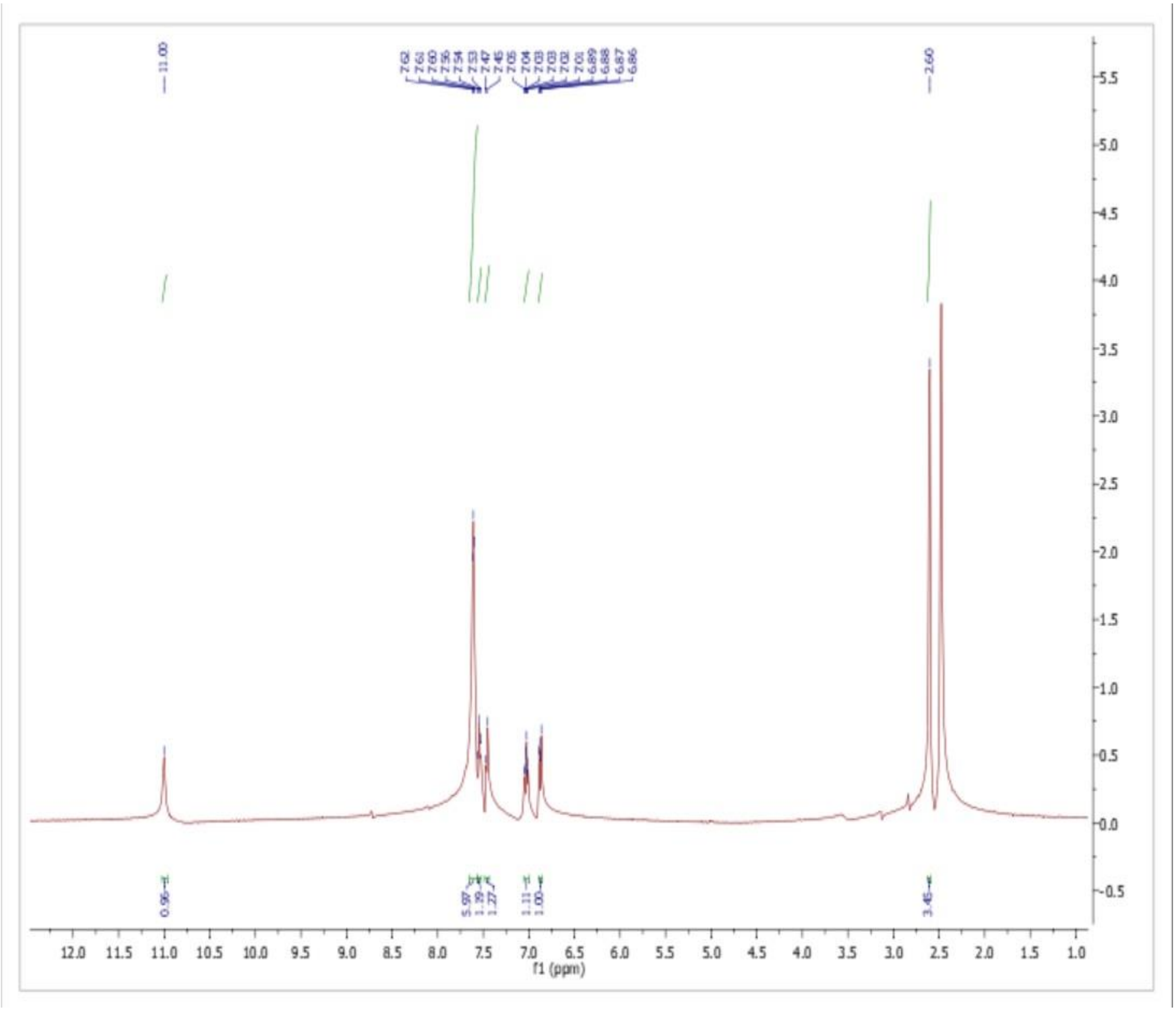
11.00
7.62
7.61
7.60
7.56
7.54
7.53
7.47
7.45
7.05
7.04
7.03
7.03
7.02
7.01
6.89
6.88
6.87
6.86
2.60
0.96
5.97
1.19
1.27
1.11
1.00
3.45
f1 (ppm)

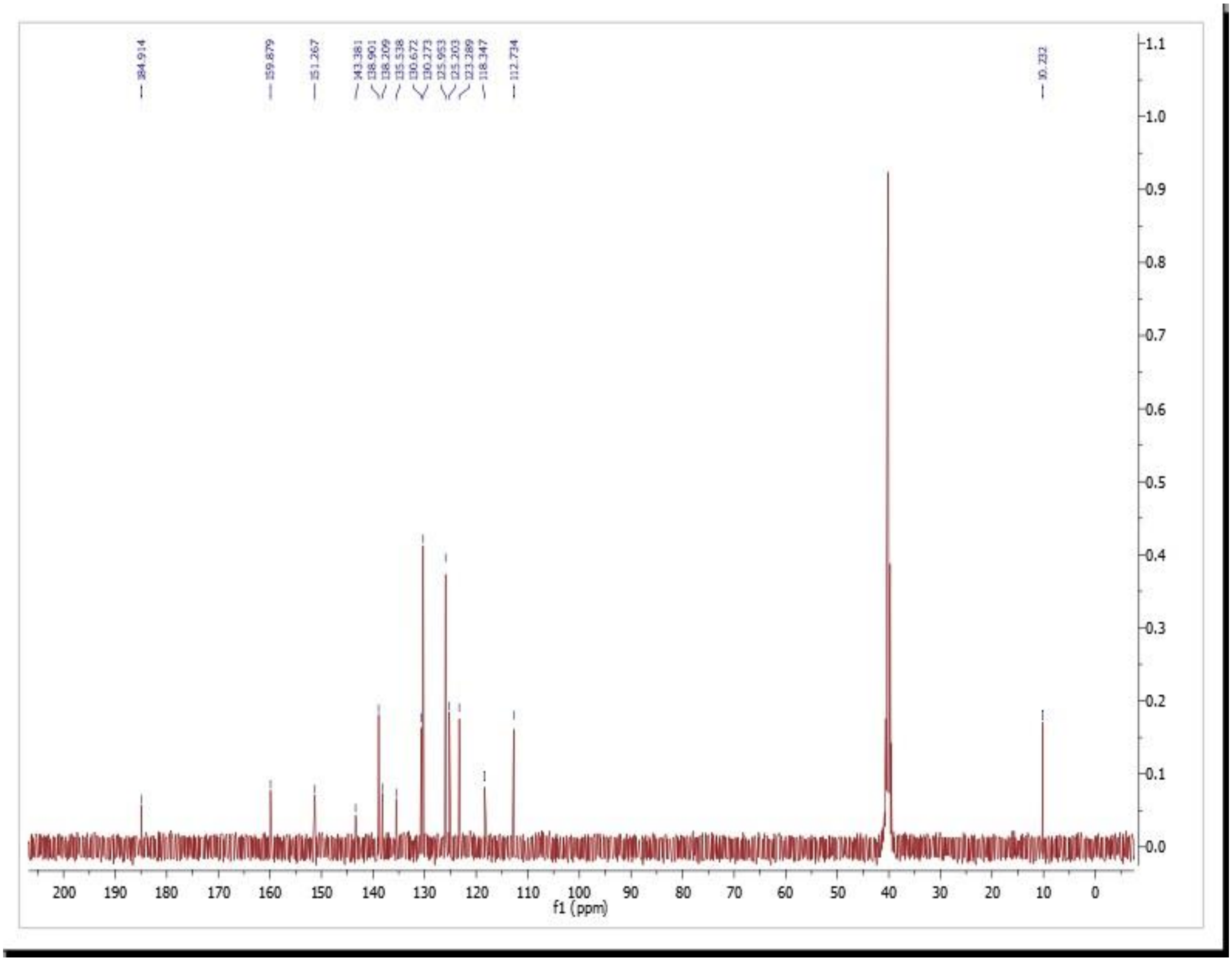
184.914
159.879
151.267
143.381
138.901
138.209
135.538
130.672
130.273
125.953
125.203
123.289
118.347
112.734
10.232
1.1
1.0
0.9
0.8
0.7
0.6
0.5
0.4
0.3
0.2
0.1
0.0
200 190 180 170 160 150 140 130 120 110 100 90 80 70 60 50 40 30 20 10 0
f1 (ppm)

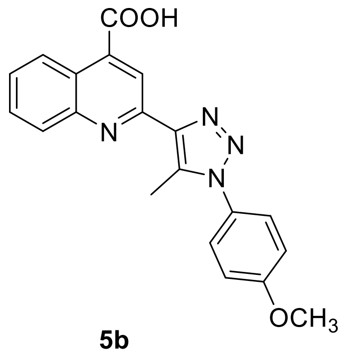
COOH
N
N
N
N
OCH3

5b

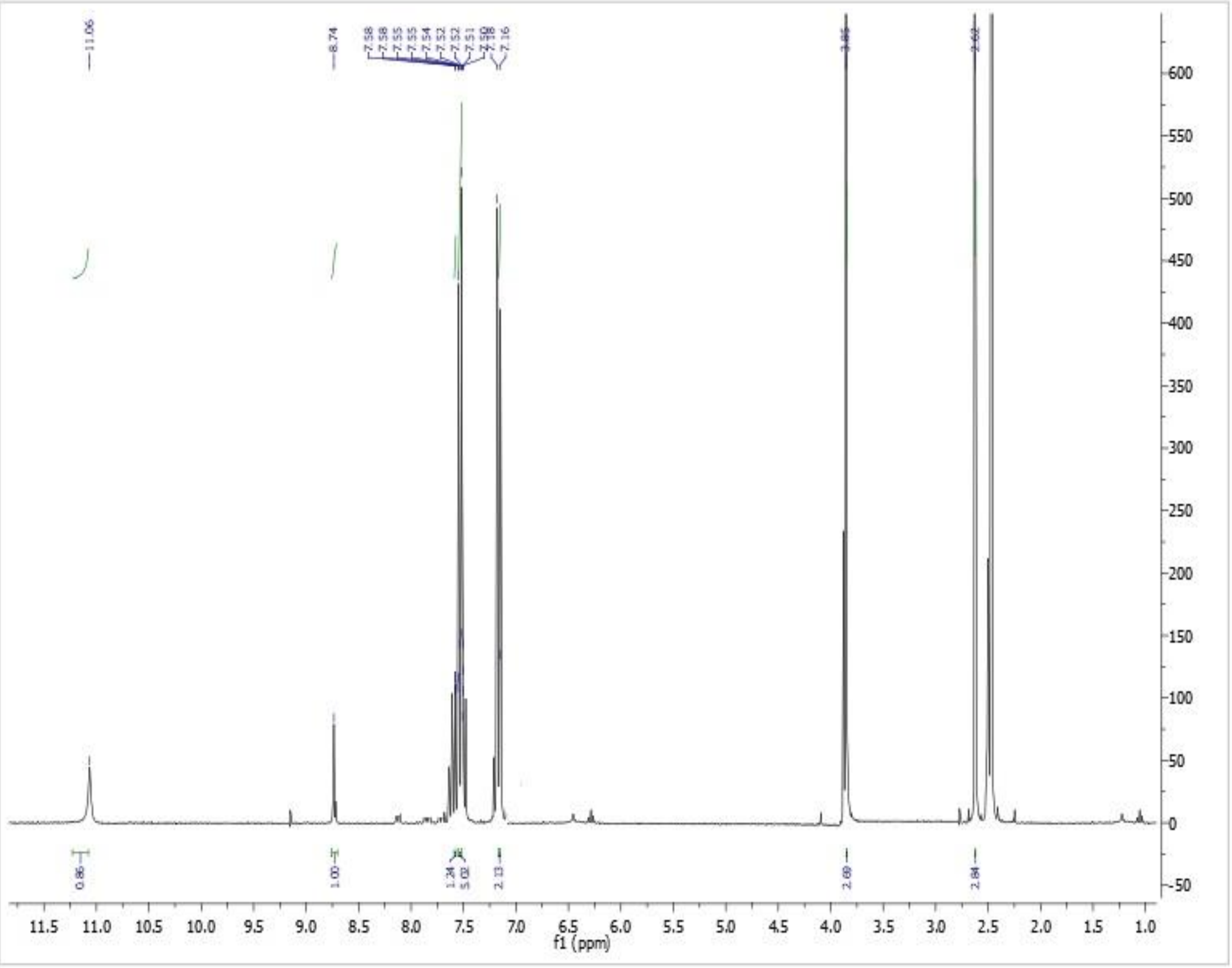
11.06
8.74
7.58
7.58
7.55
7.55
7.54
7.52
7.52
7.51
7.50
7.18
7.16
3.85
2.62
0.86
1.00
1.24
5.02
2.13
2.69
2.84
f1 (ppm)

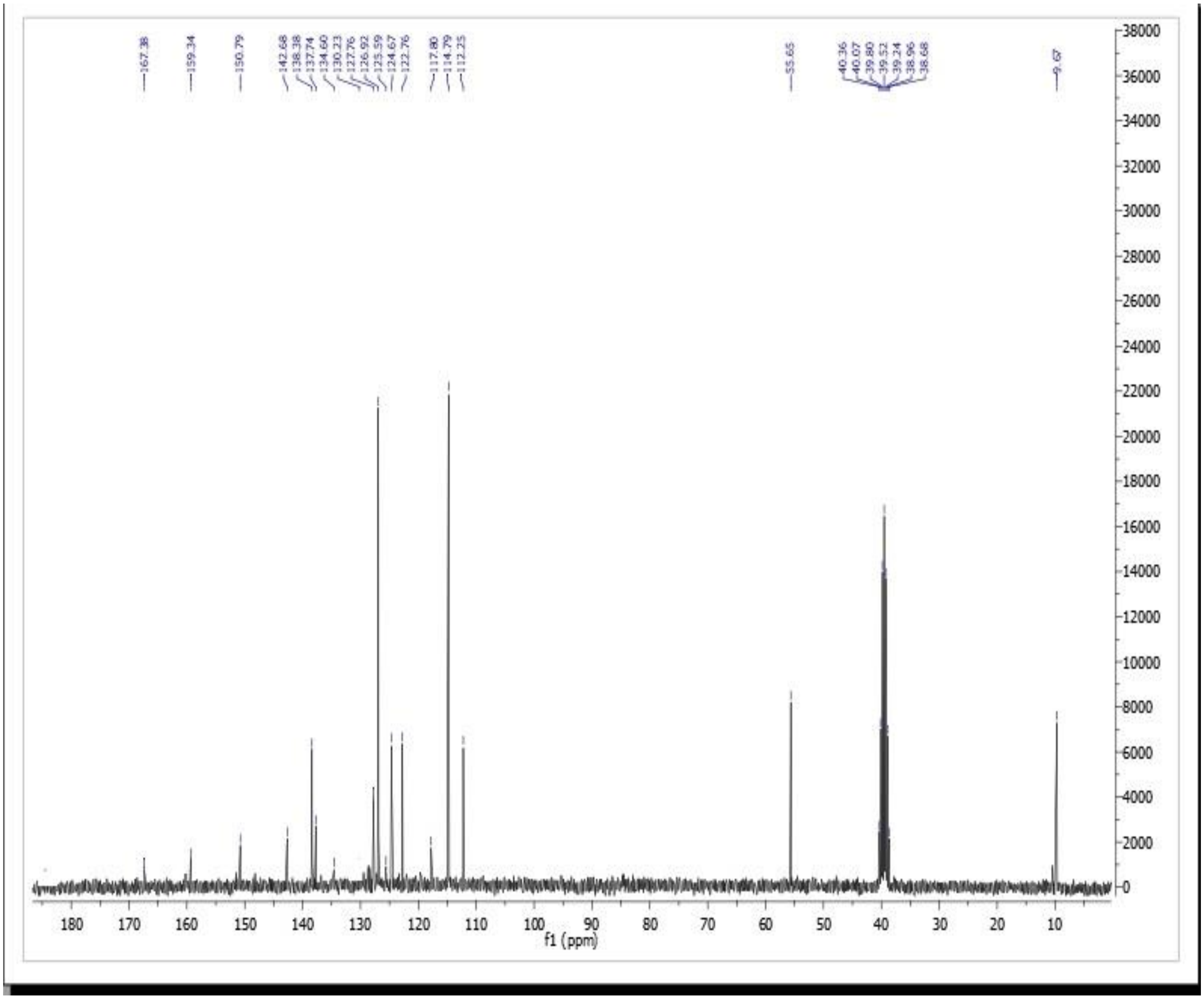

167.38
159.34
150.79
142.68
138.38
137.74
134.60
130.23
127.76
126.92
125.59
124.67
122.76
117.80
114.79
112.25
55.65
40.36
40.07
39.80
39.52
39.24
38.96
38.68
9.67
f1 (ppm)

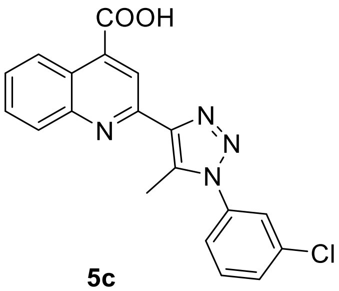

5c

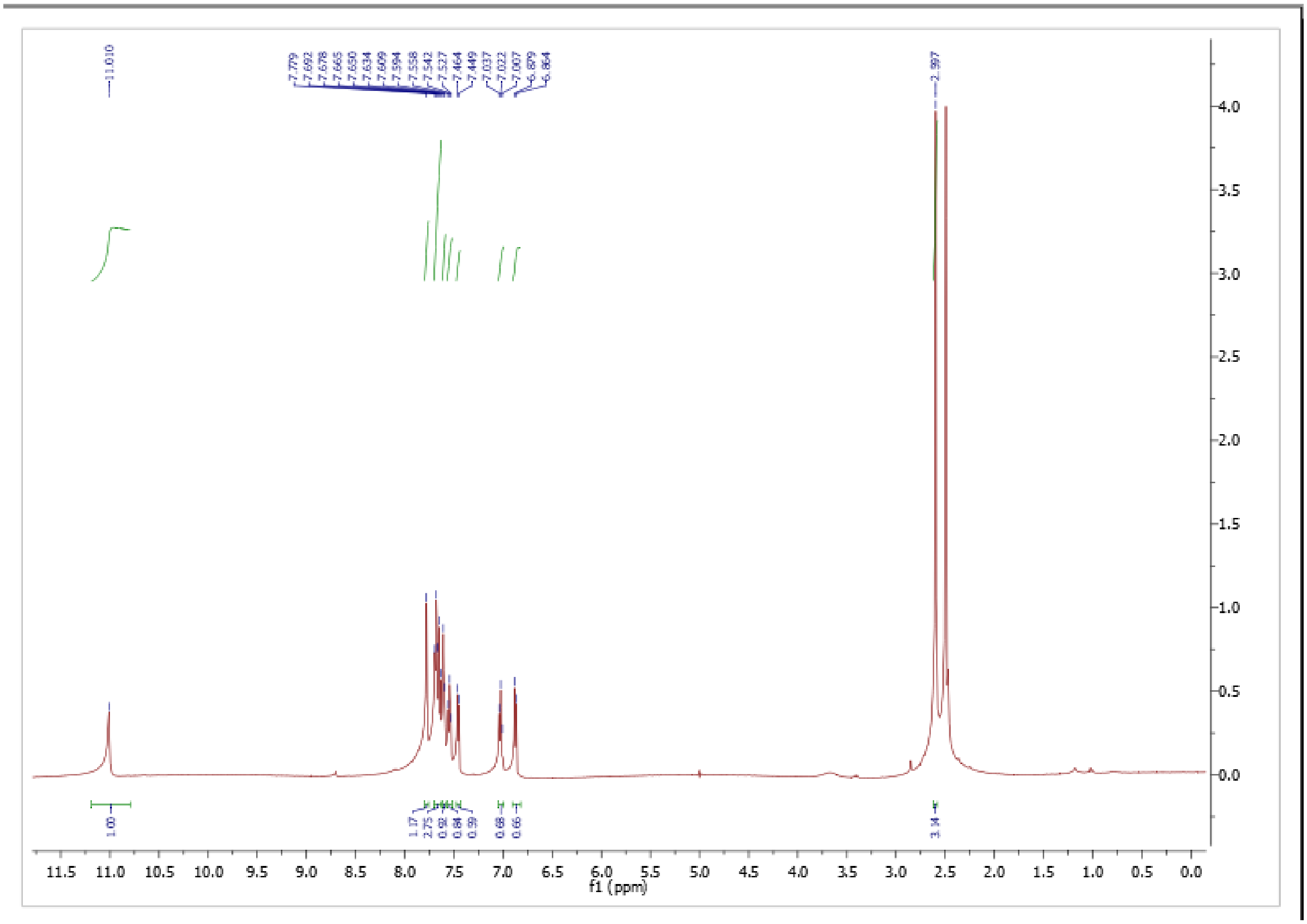

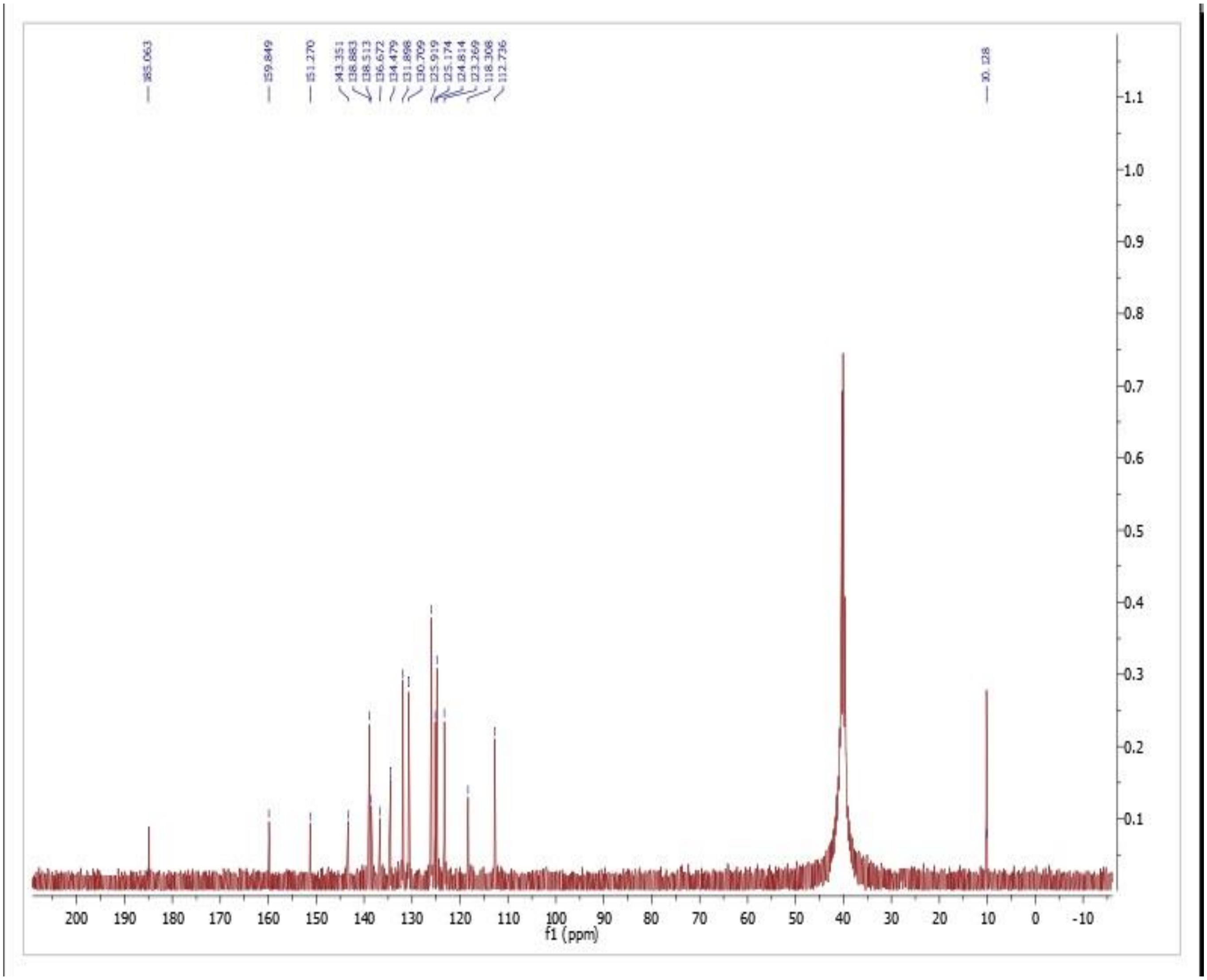
185.063
159.849
151.270
143.351
138.883
138.513
136.672
134.479
131.898
130.709
125.919
125.174
124.814
123.269
118.308
112.736
10.128
f1 (ppm)

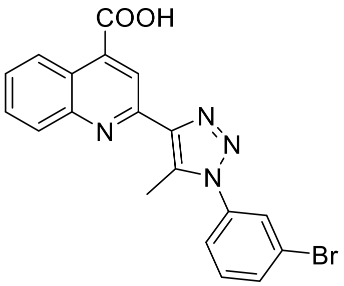
COOH
N
N
N
N
Br

5d

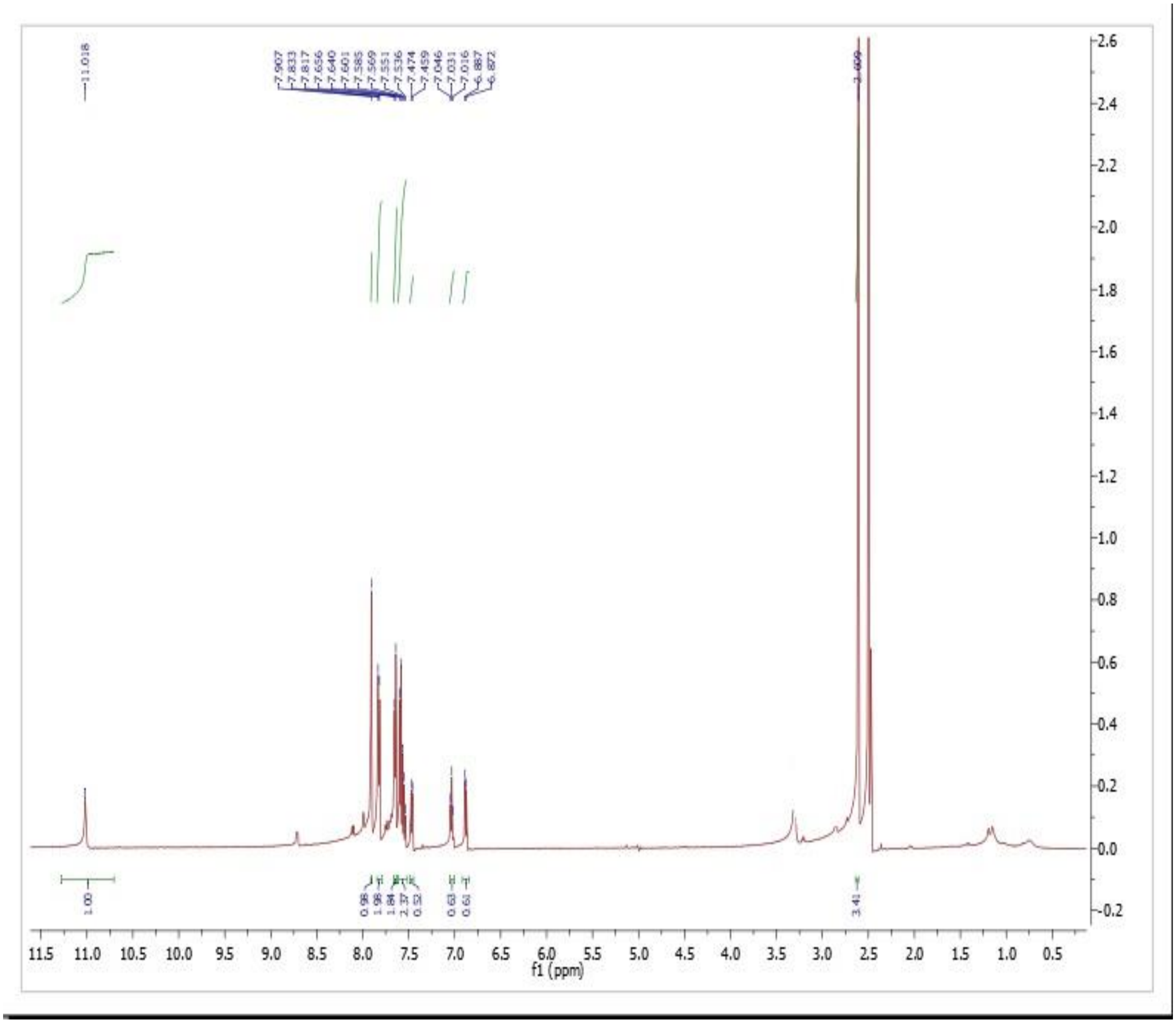
11.018
7.907
7.833
7.817
7.656
7.640
7.601
7.585
7.569
7.551
7.536
7.474
7.459
7.046
7.031
7.016
6.887
6.872
2.609
1.00
0.98
1.98
1.84
2.37
0.52
0.63
0.61
3.41
f1 (ppm)

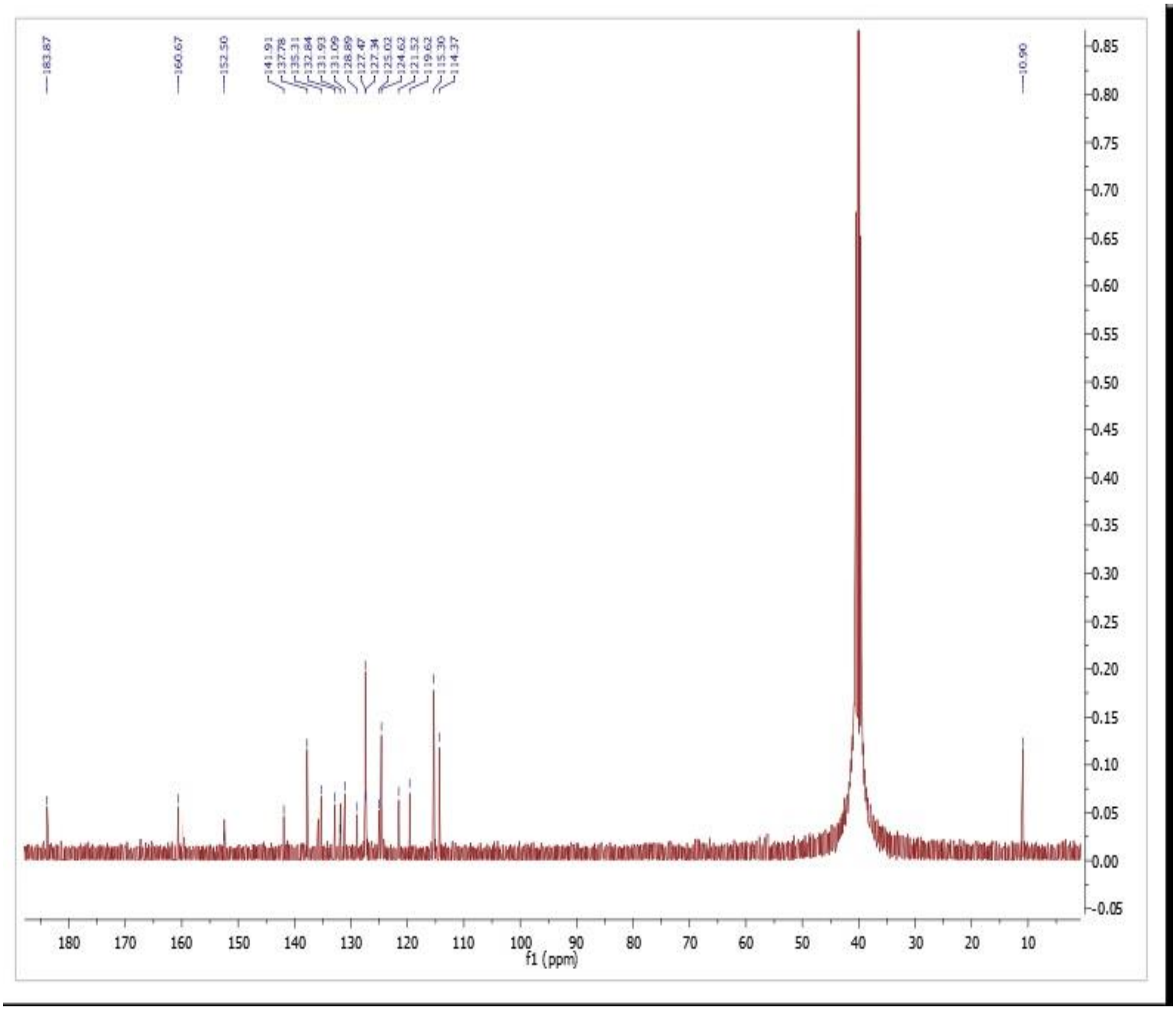
183.87
160.67
152.50
141.91
137.78
135.31
132.84
131.93
131.09
128.89
127.47
127.34
125.02
124.62
121.52
119.62
115.30
114.37
10.90
f1 (ppm)

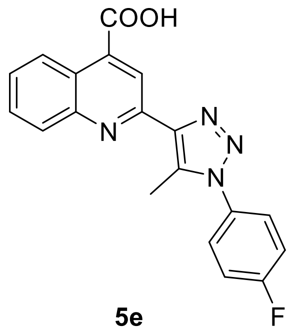
COOH
N
N
N
N
F

5e

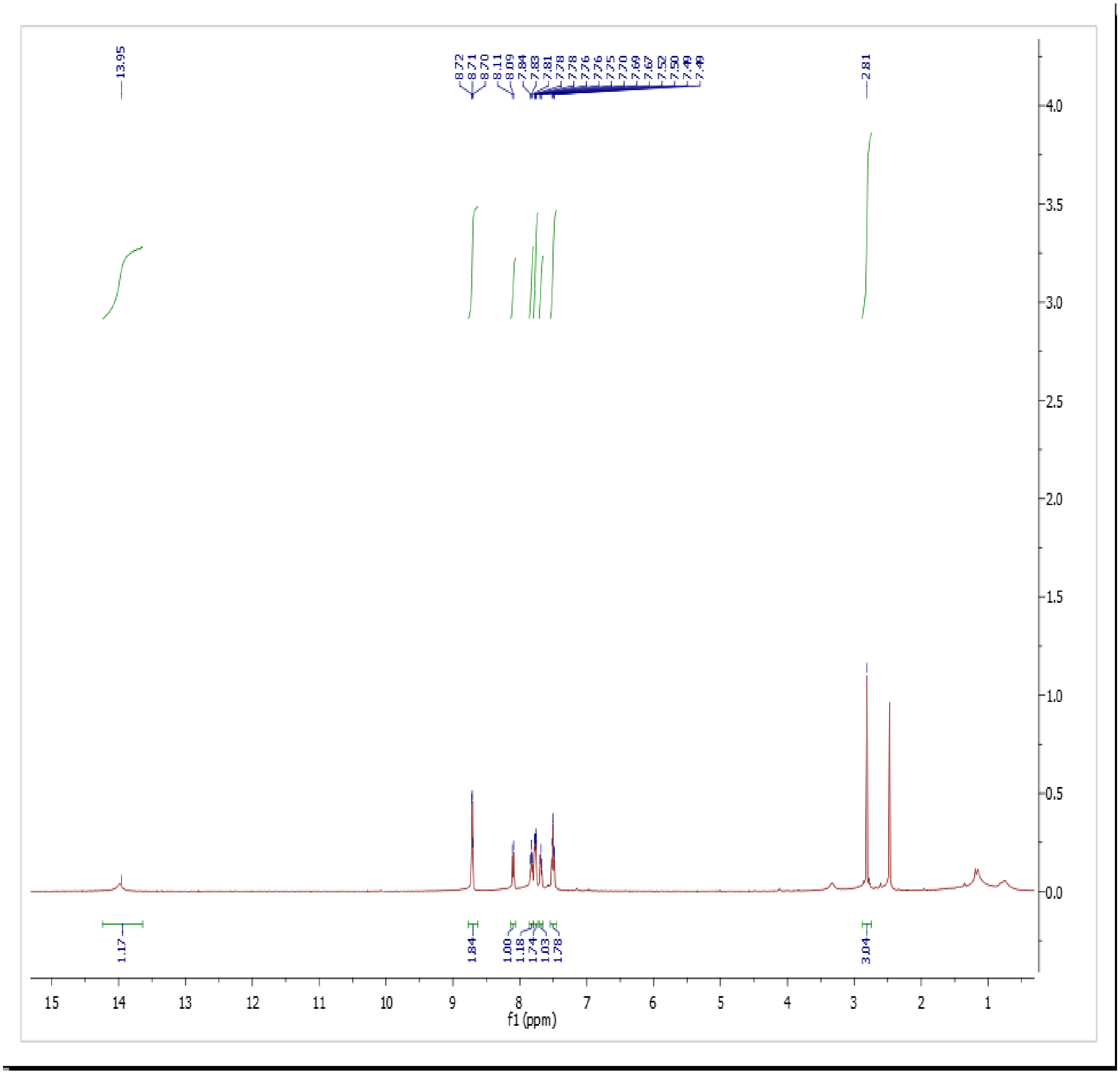
13.95
8.72
8.71
8.70
8.11
8.09
7.84
7.83
7.81
7.78
7.76
7.76
7.75
7.70
7.69
7.67
7.52
7.50
7.49
7.49
2.81
1.17
1.84
1.00
1.18
1.74
1.03
1.78
3.04
f1 (ppm)
15 14 13 12 11 10 9 8 7 6 5 4 3 2 1
4.0 3.5 3.0 2.5 2.0 1.5 1.0 0.5 0.0

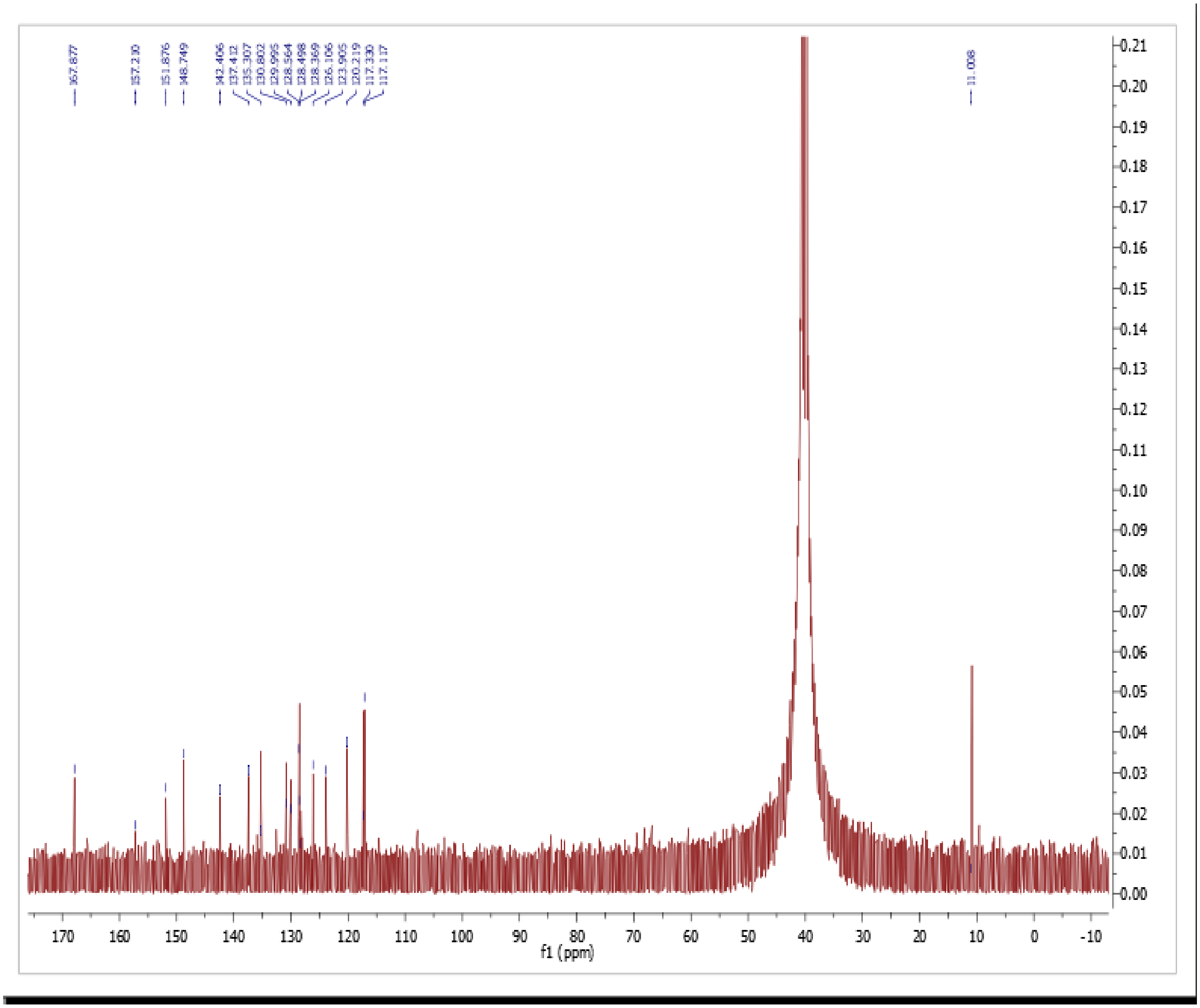
167.877
157.230
151.876
148.749
142.406
137.412
135.307
130.802
129.995
128.564
128.498
128.369
126.106
123.905
120.219
117.330
117.117
11.008
f1 (ppm)

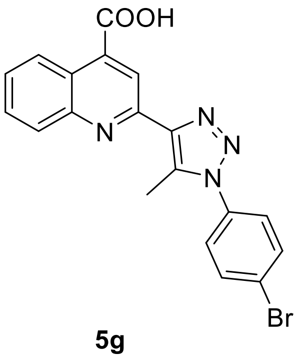

5g

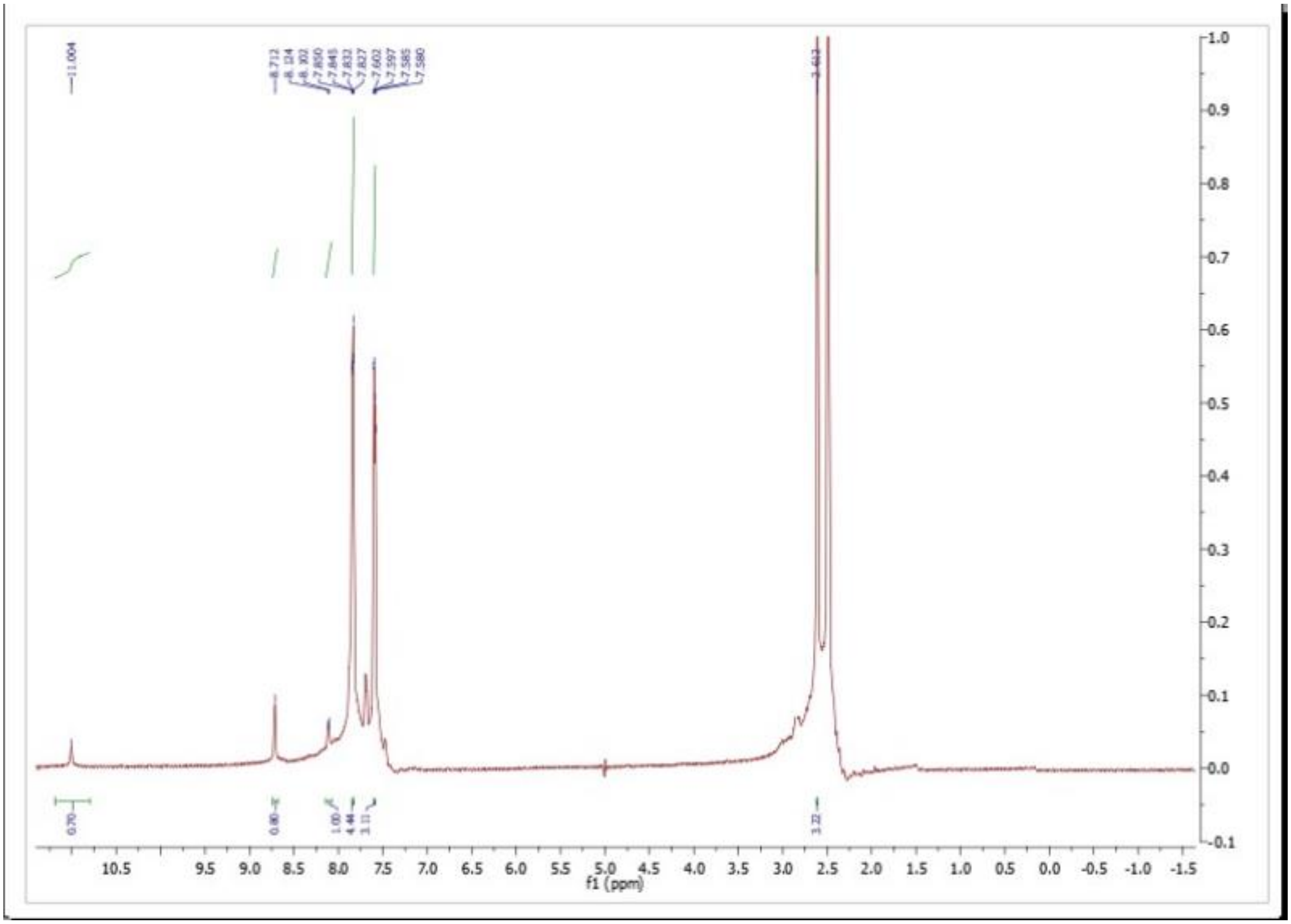

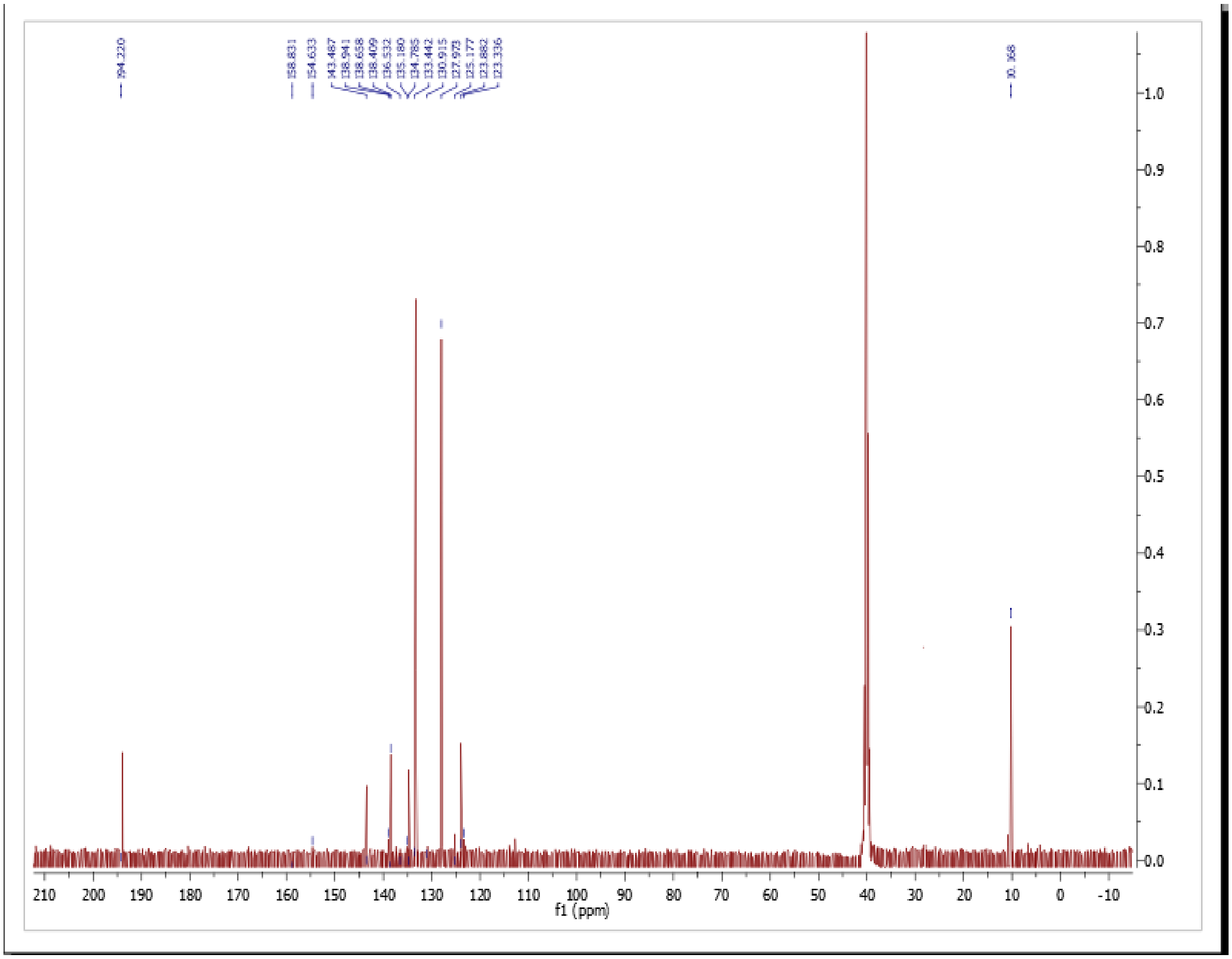
194.220
158.831
154.633
143.487
138.941
138.658
138.409
136.532
135.180
134.785
133.442
130.915
127.973
125.177
123.882
123.336
10.168
1.0
0.9
0.8
0.7
0.6
0.5
0.4
0.3
0.2
0.1
0.0
210 200 190 180 170 160 150 140 130 120 110 100 90 80 70 60 50 40 30 20 10 0 -10
f1 (ppm)

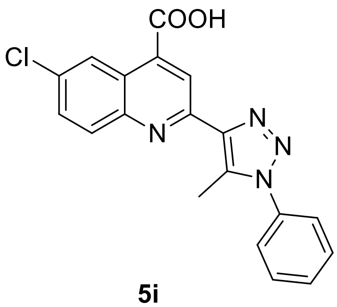

5i

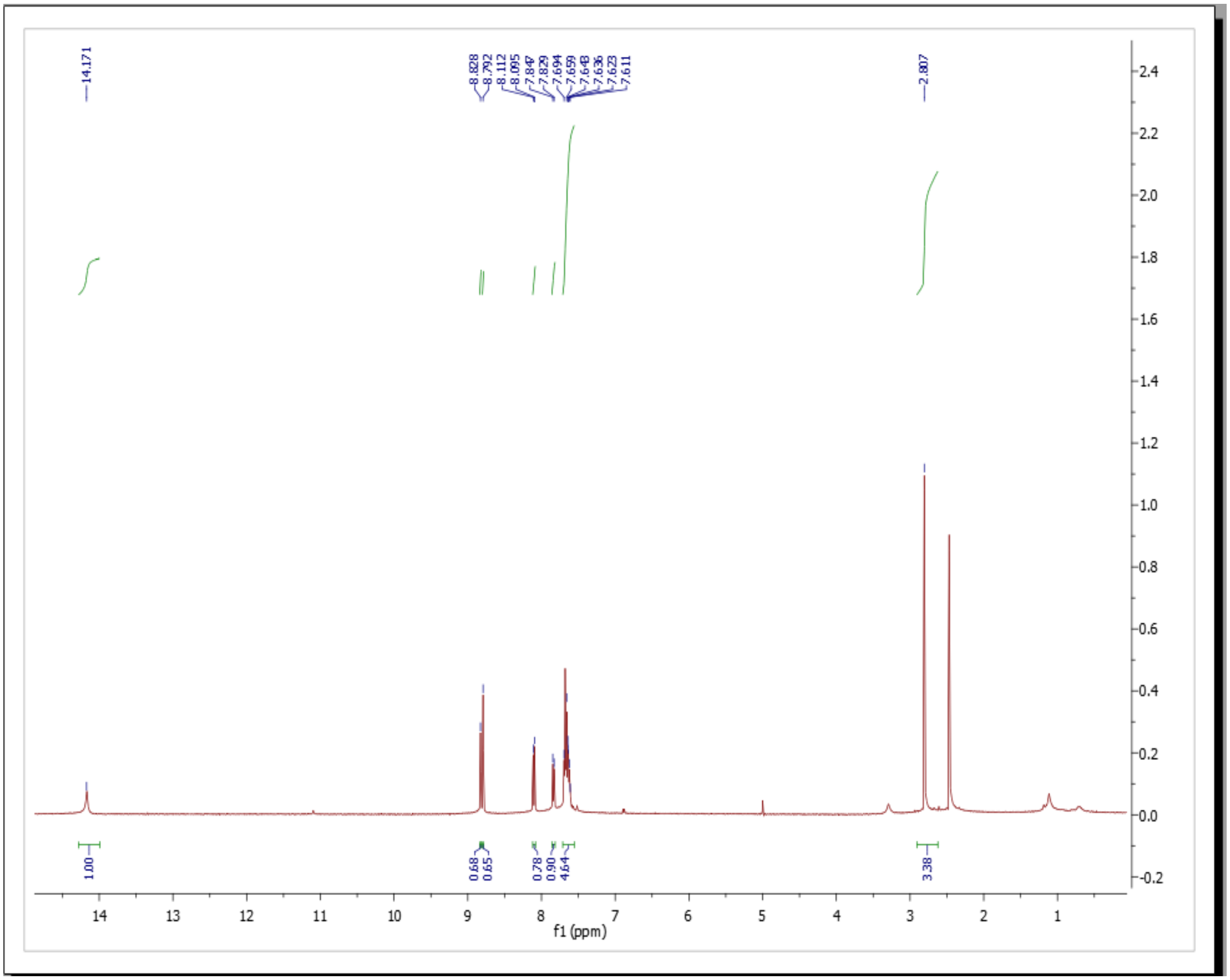
14.171
8.828
8.792
8.112
8.095
7.847
7.829
7.694
7.659
7.643
7.636
7.623
7.611
2.807
1.00
0.68
0.65
0.78
0.90
4.64
3.38
f1 (ppm)

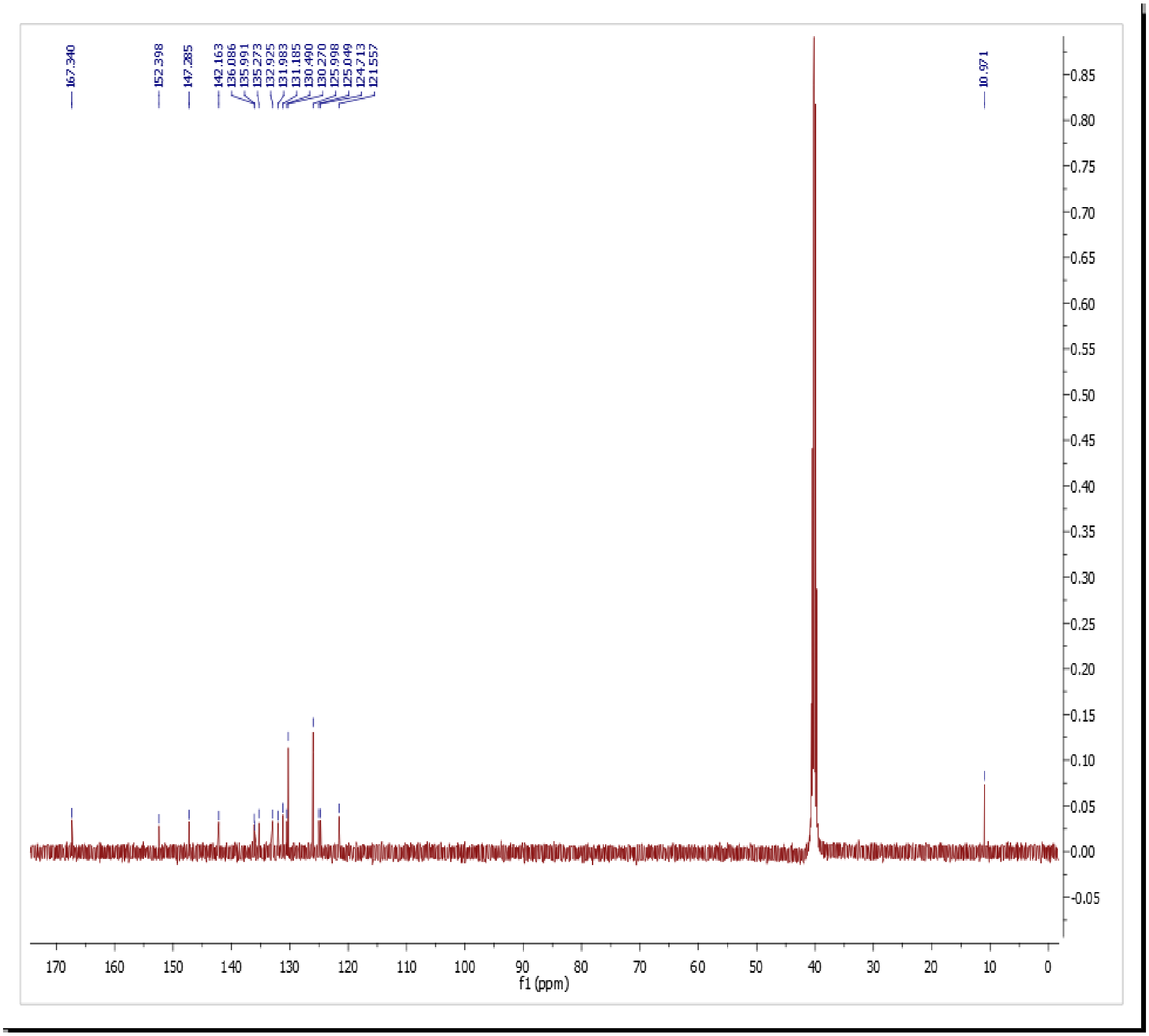

167.340
152.398
147.285
142.163
136.086
135.991
135.273
132.925
131.983
131.185
130.490
130.270
125.998
125.049
124.713
121.557
10.971
0.85
0.80
0.75
0.70
0.65
0.60
0.55
0.50
0.45
0.40
0.35
0.30
0.25
0.20
0.15
0.10
0.05
0.00
-0.05
170
160
150
140
130
120
110
100
90
80
70
60
50
40
30
20
10
0
f1 (ppm)

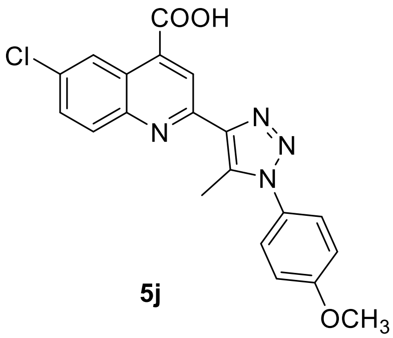
COOH
Cl
N
N
N
N
OCH3

5j

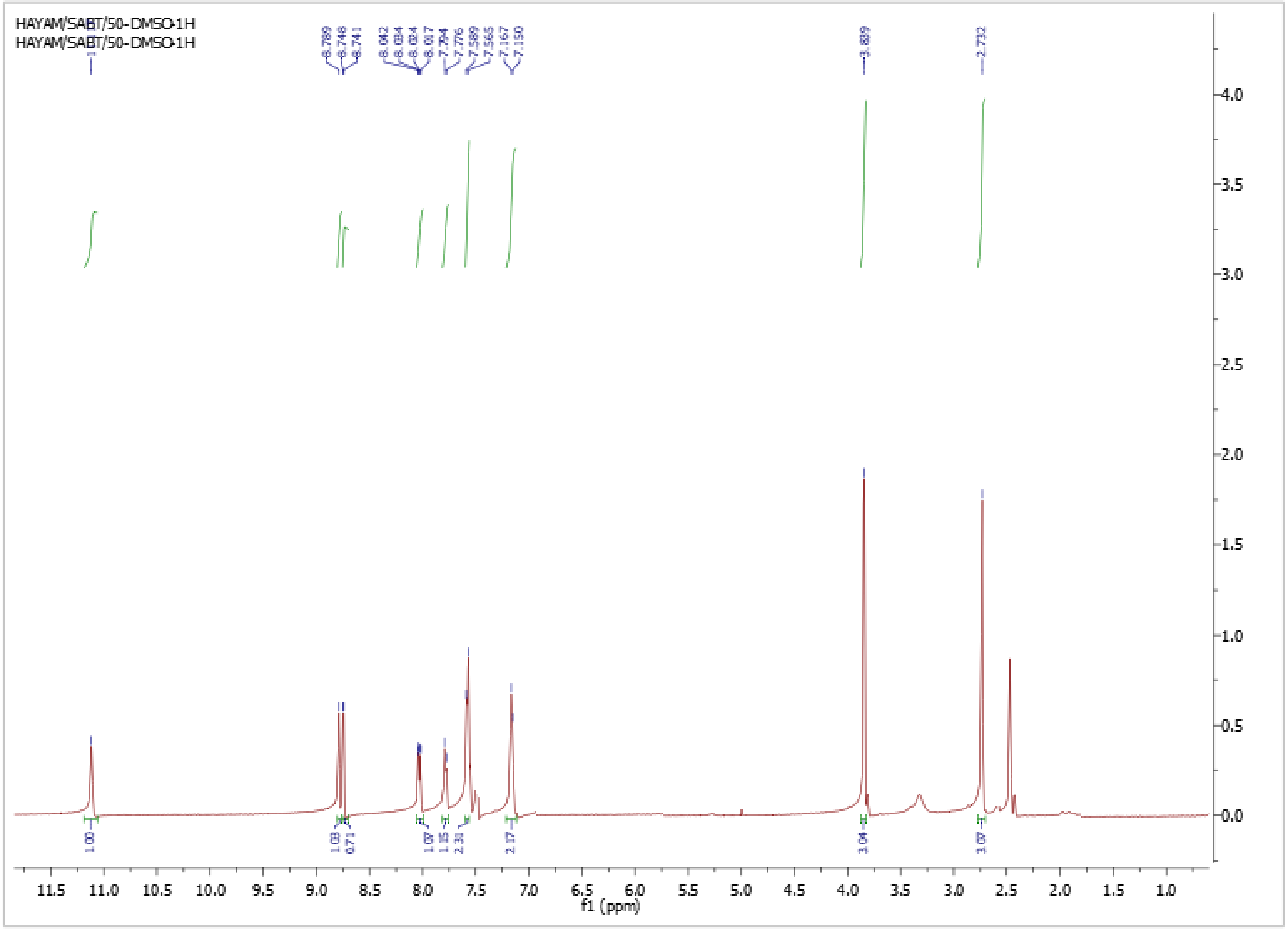
HAYAM/SABT/50-DMSO-1H
8.789
8.748
8.741
8.042
8.034
8.024
8.017
7.794
7.776
7.589
7.565
7.167
7.150
3.839
2.732
1.00
1.03
0.71
1.07
1.15
2.31
2.17
3.04
3.07
f1 (ppm)

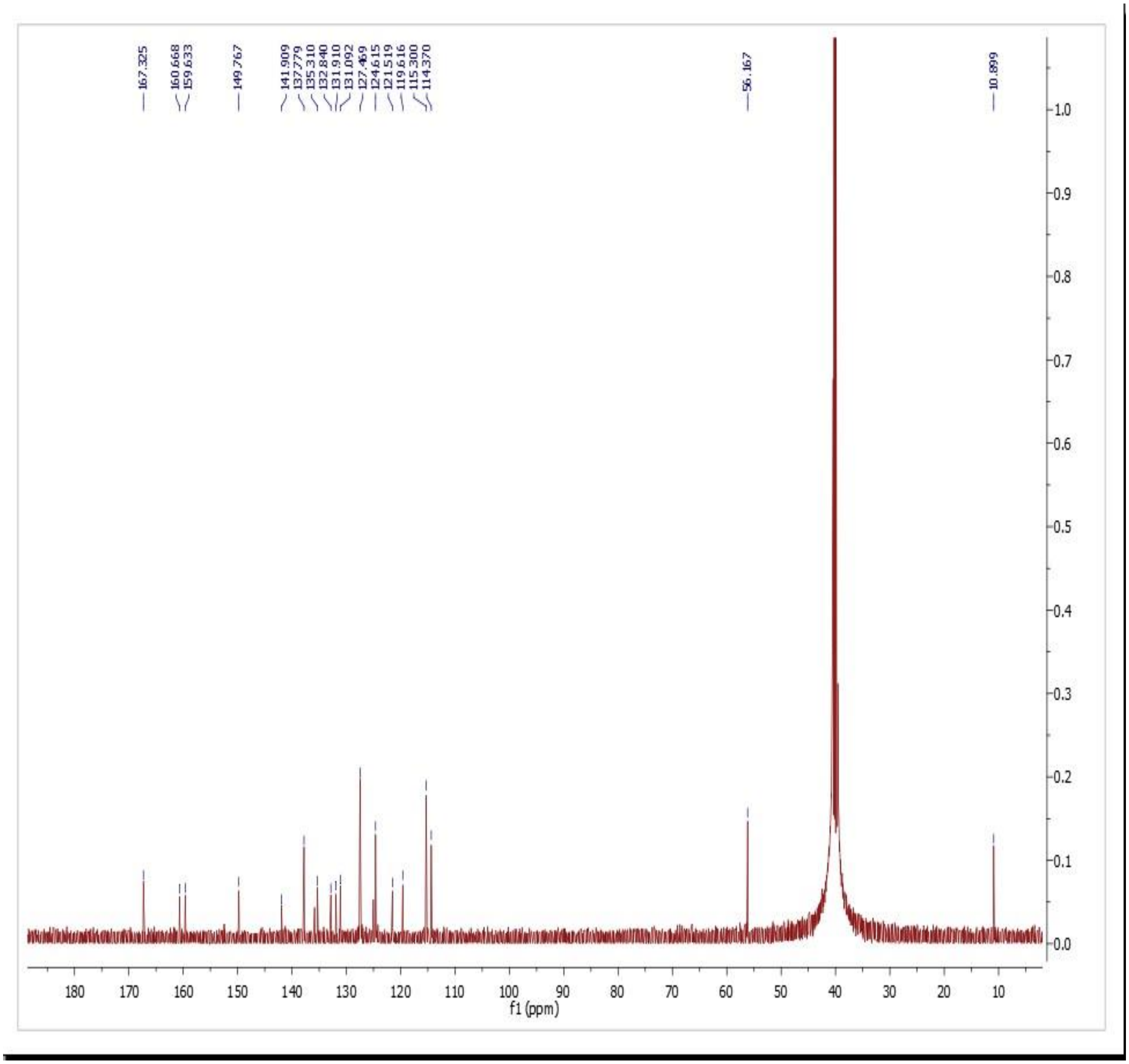

167.325
160.668
159.633
149.767
141.909
137.779
135.310
132.840
131.910
131.092
127.469
124.615
121.519
119.616
115.300
114.370
56.167
10.899
1.0
0.9
0.8
0.7
0.6
0.5
0.4
0.3
0.2
0.1
0.0
180 170 160 150 140 130 120 110 100 90 80 70 60 50 40 30 20 10
f1 (ppm)

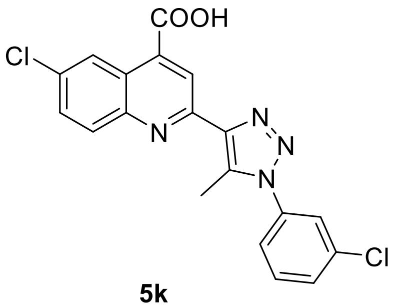
COOH
Cl
N
N
N
N
Cl

5k

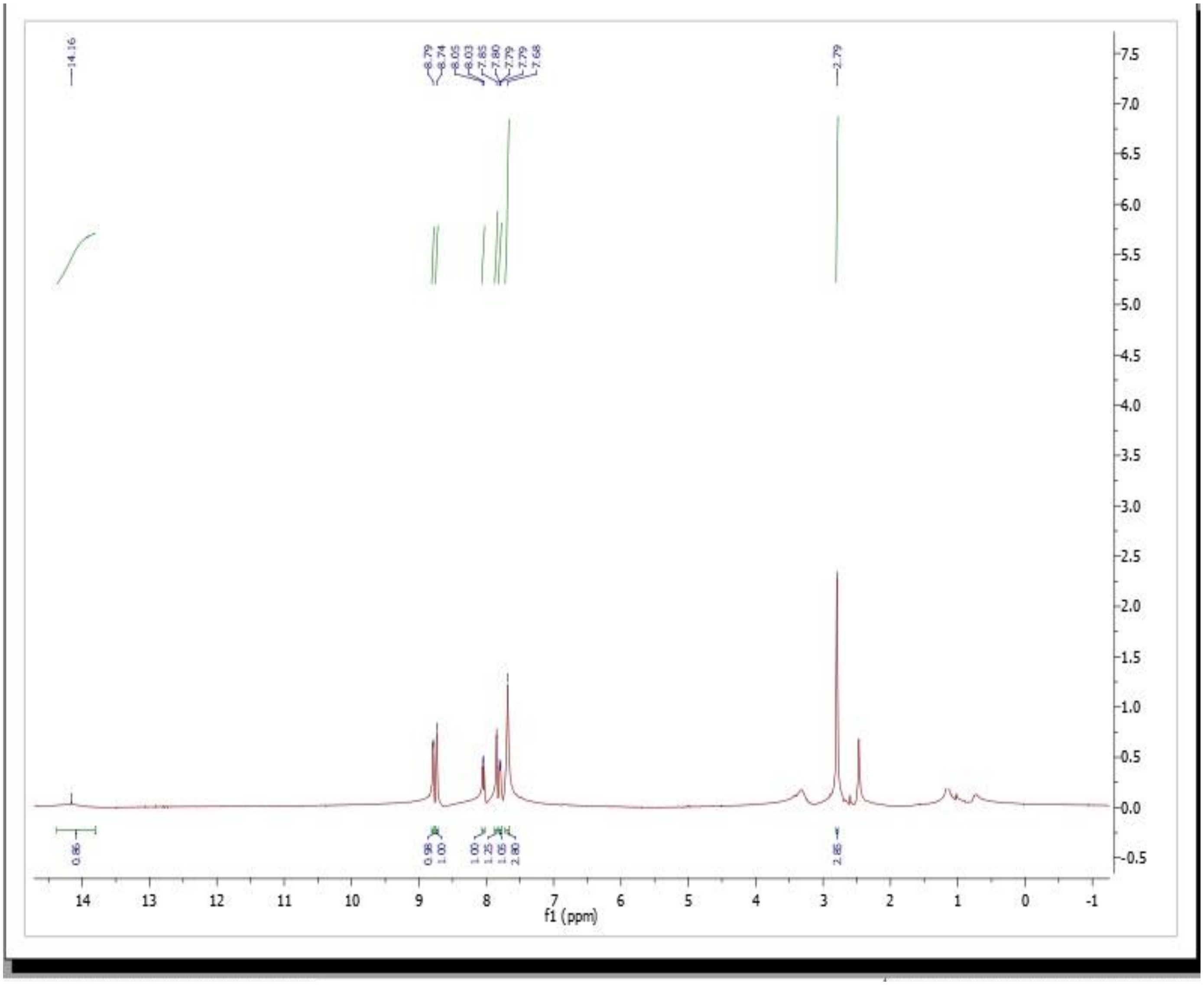
14.16
8.79
8.74
8.05
8.03
7.85
7.80
7.79
7.79
7.68
2.79
f1 (ppm)

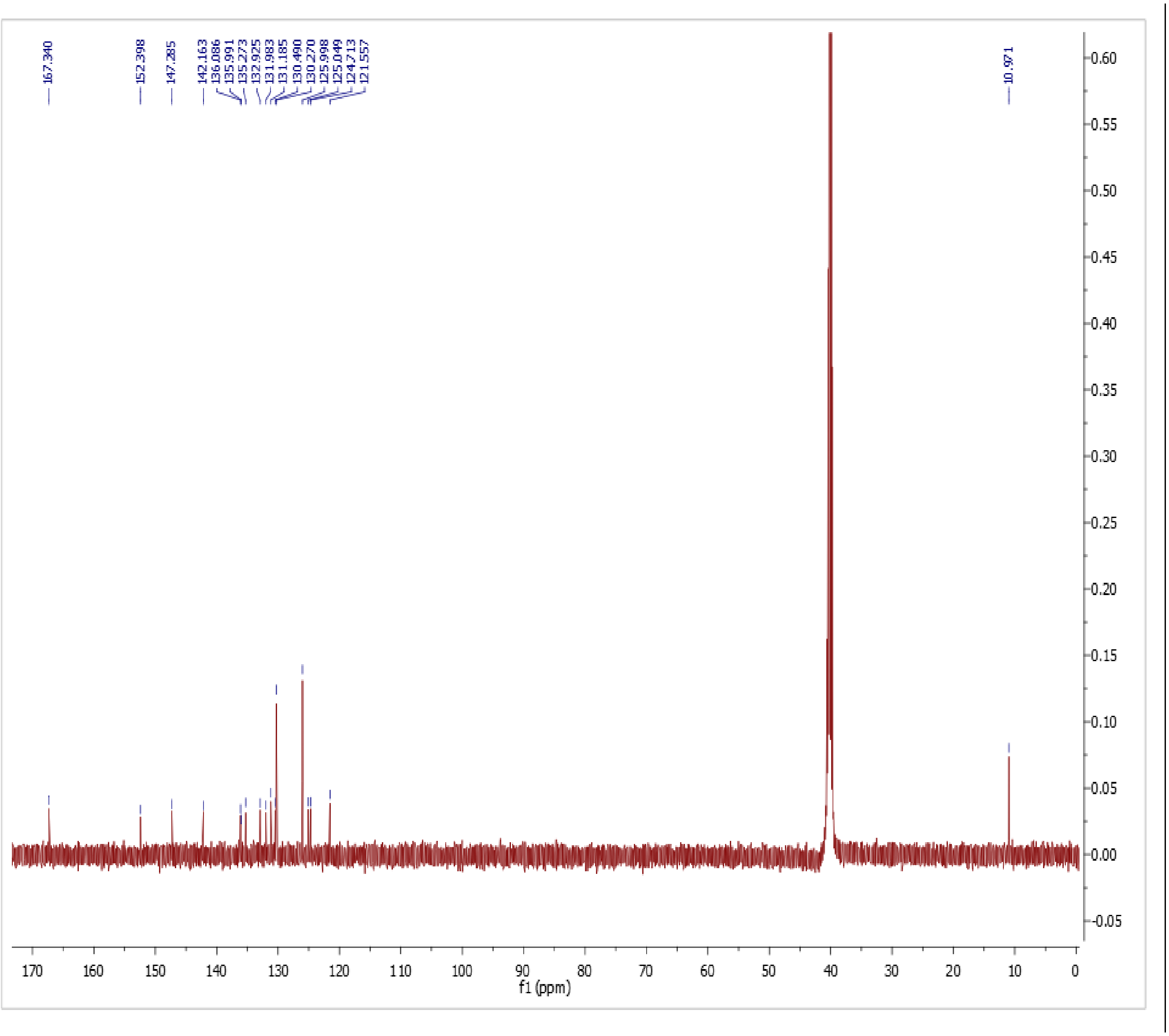

167.340
152.398
147.285
142.163
136.086
135.991
135.273
132.925
131.983
131.185
130.490
130.270
125.998
125.049
124.713
121.557
10.971
f1 (ppm)

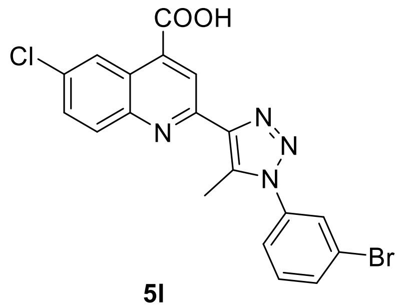

5l

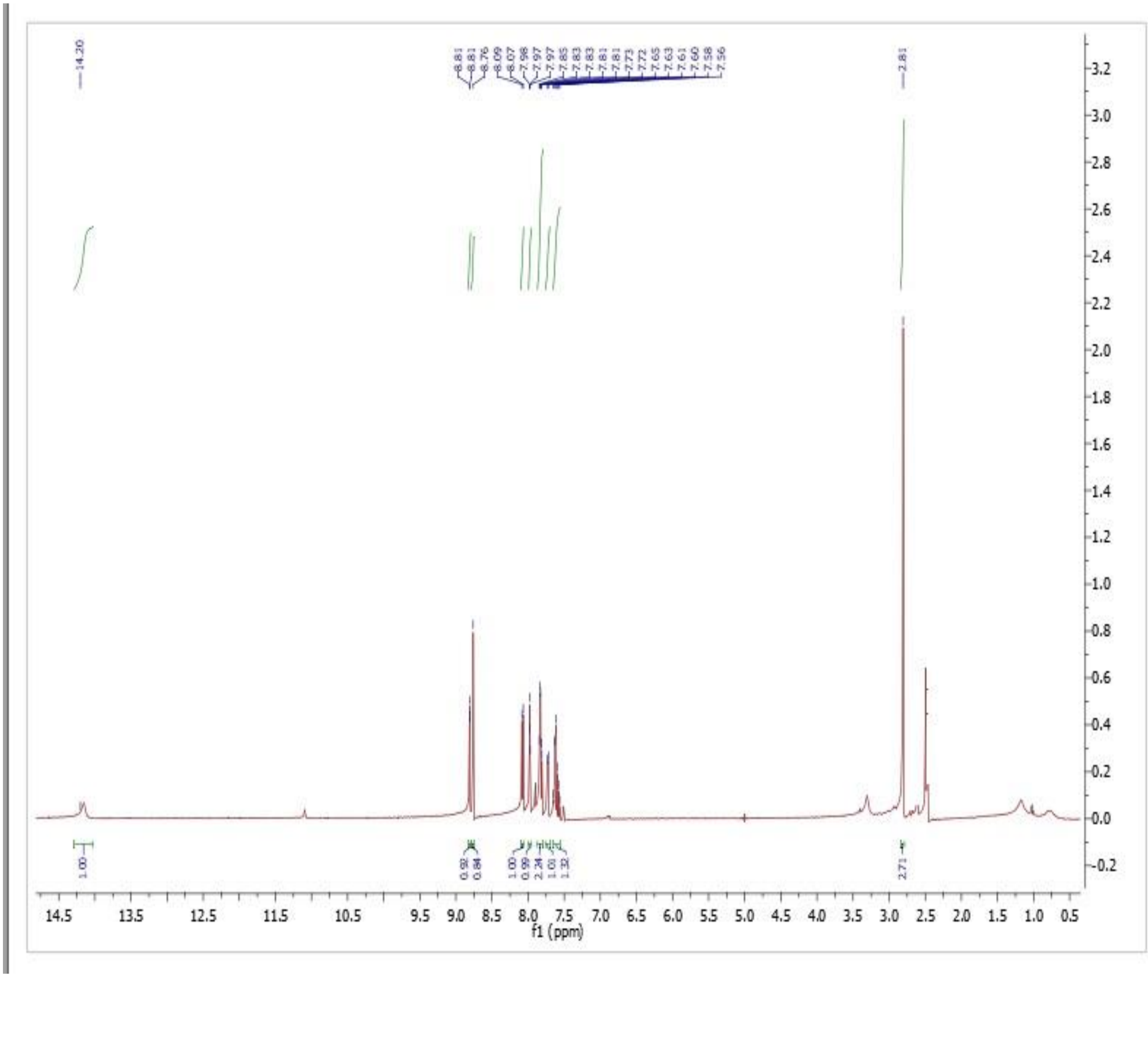

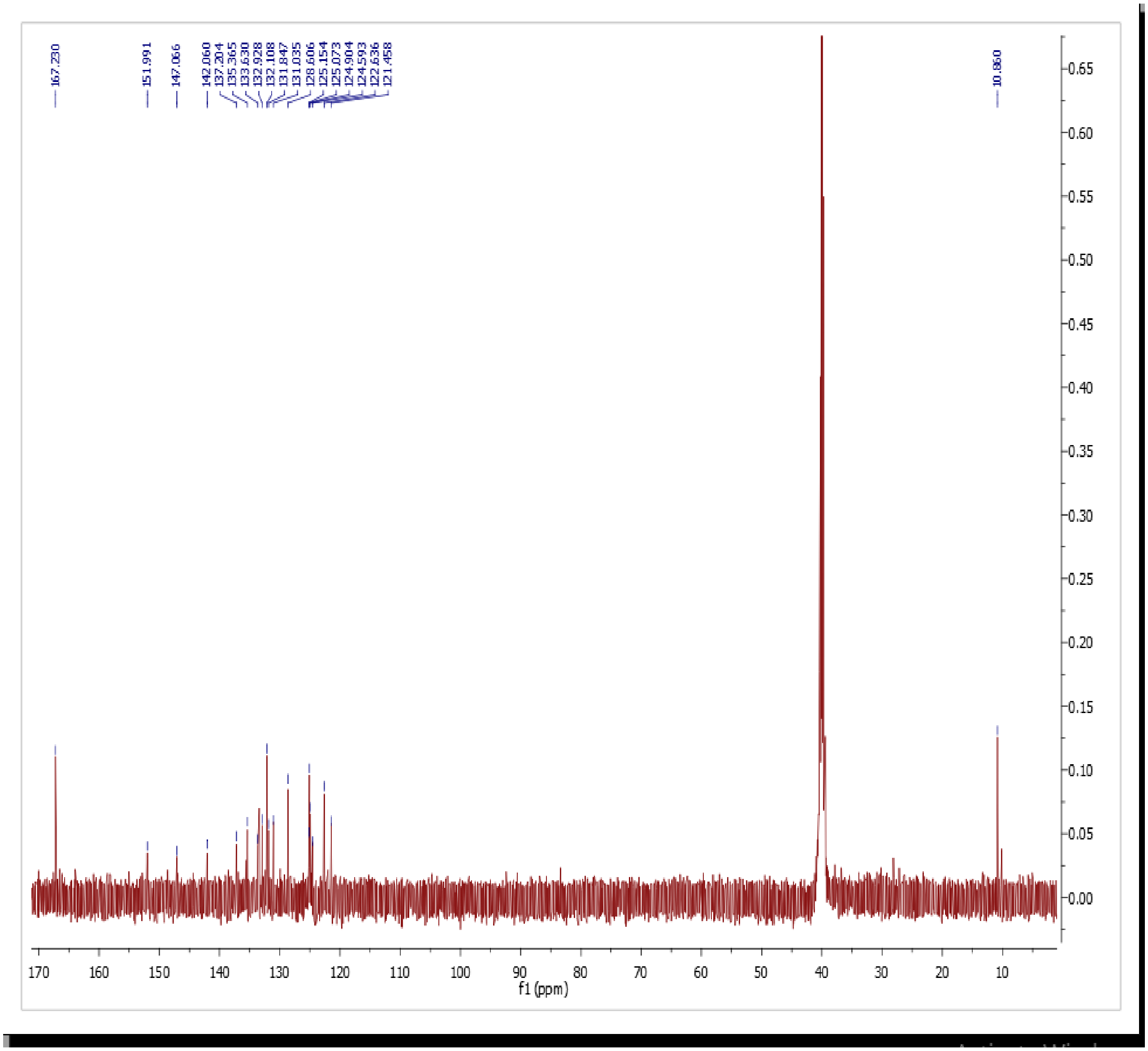
167.230
151.991
147.066
142.060
137.204
135.365
133.630
132.928
132.108
131.847
131.035
128.606
125.154
125.073
124.904
124.593
122.636
121.458
10.860
f1 (ppm)

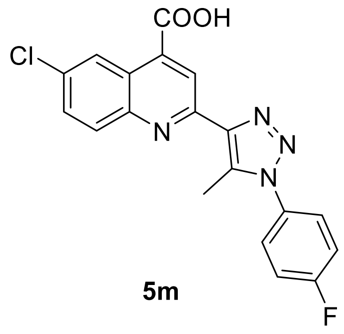

5m

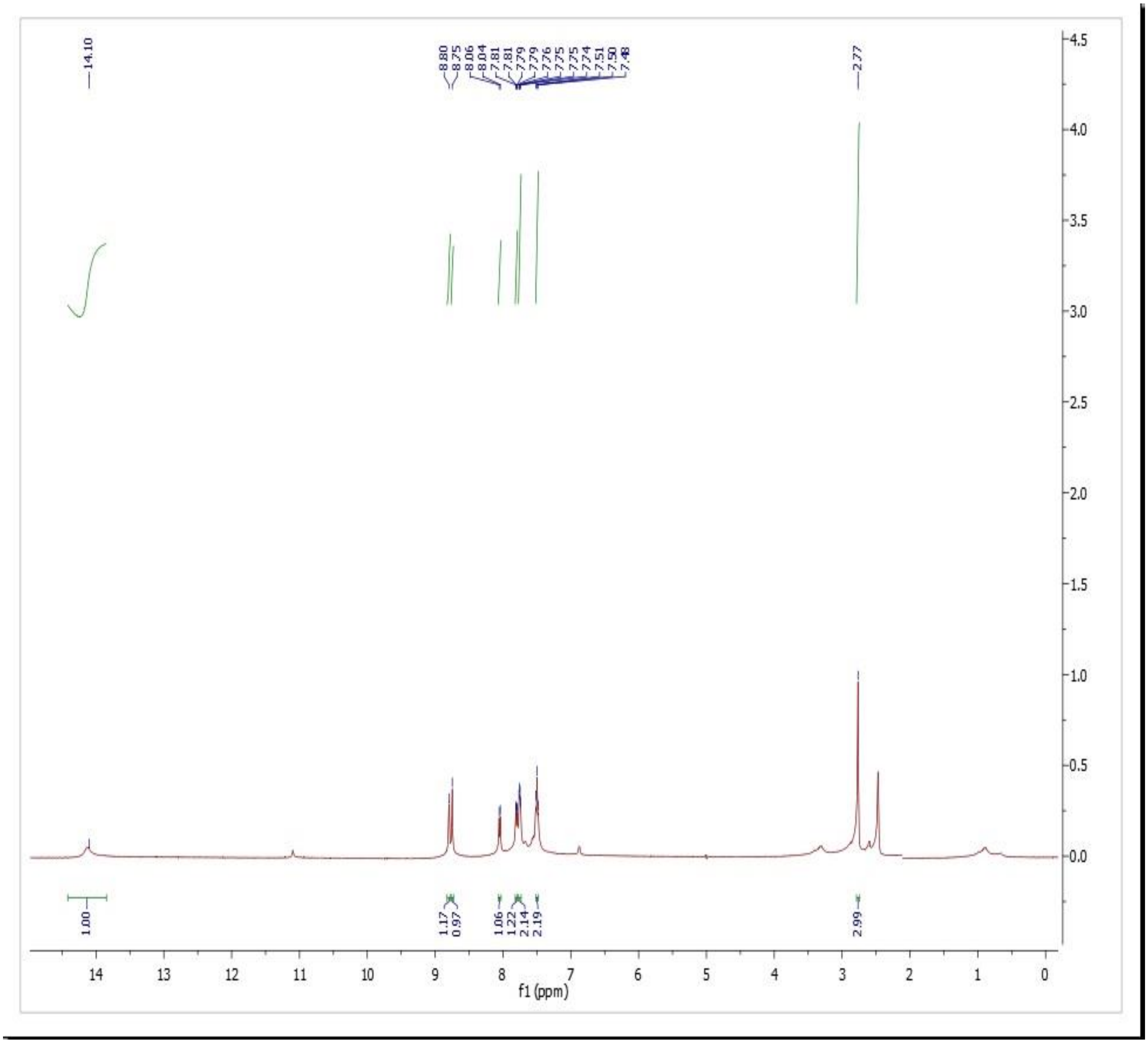

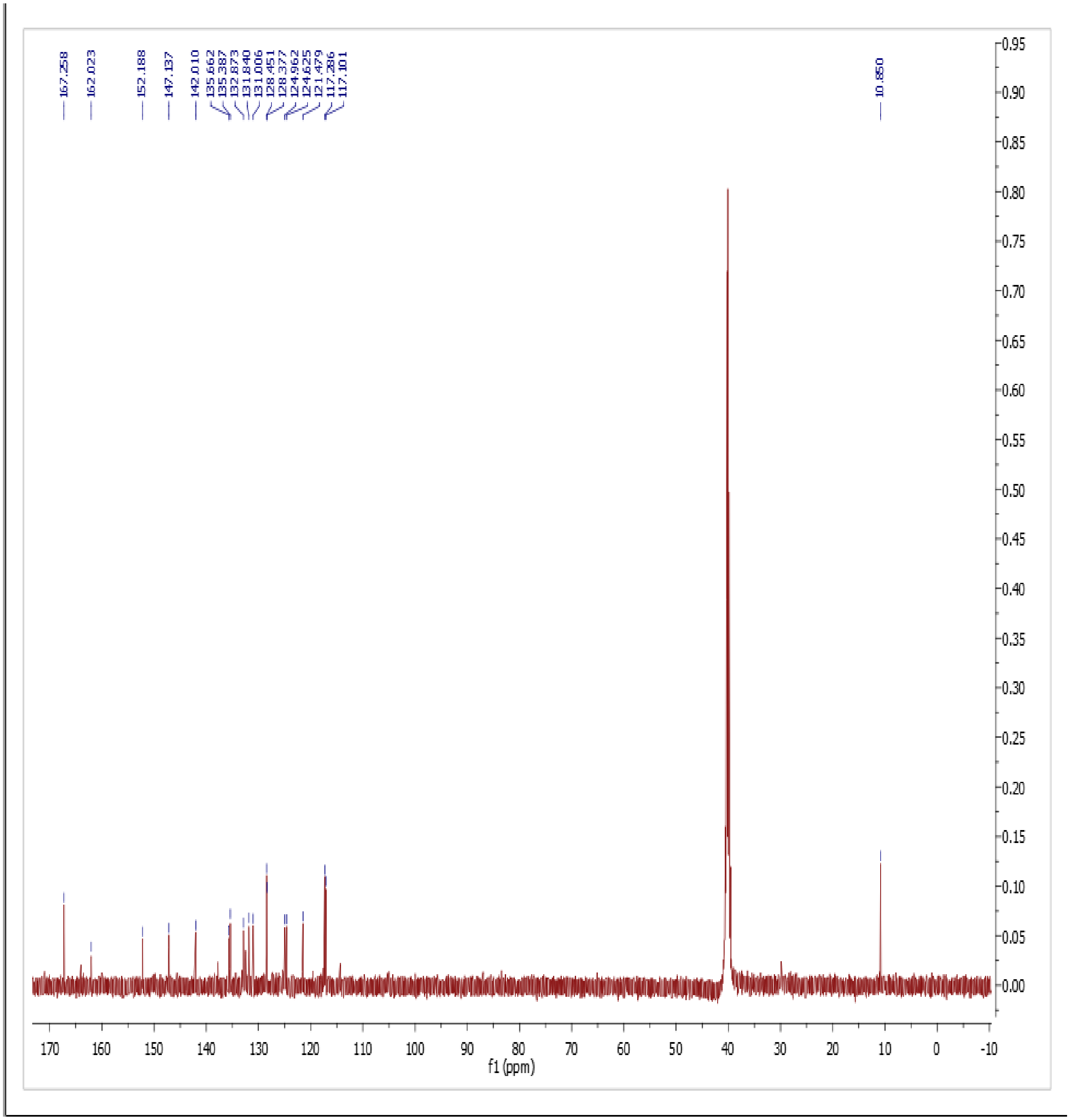
167.258
162.023
152.188
147.137
142.010
135.662
135.387
132.873
131.840
131.006
128.451
128.377
124.962
124.625
121.479
117.286
117.101
10.850
f1 (ppm)

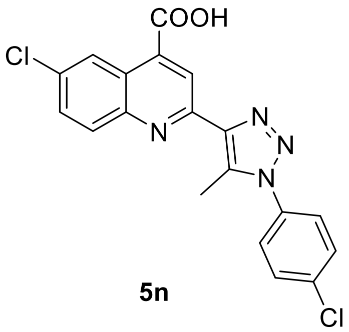

5n

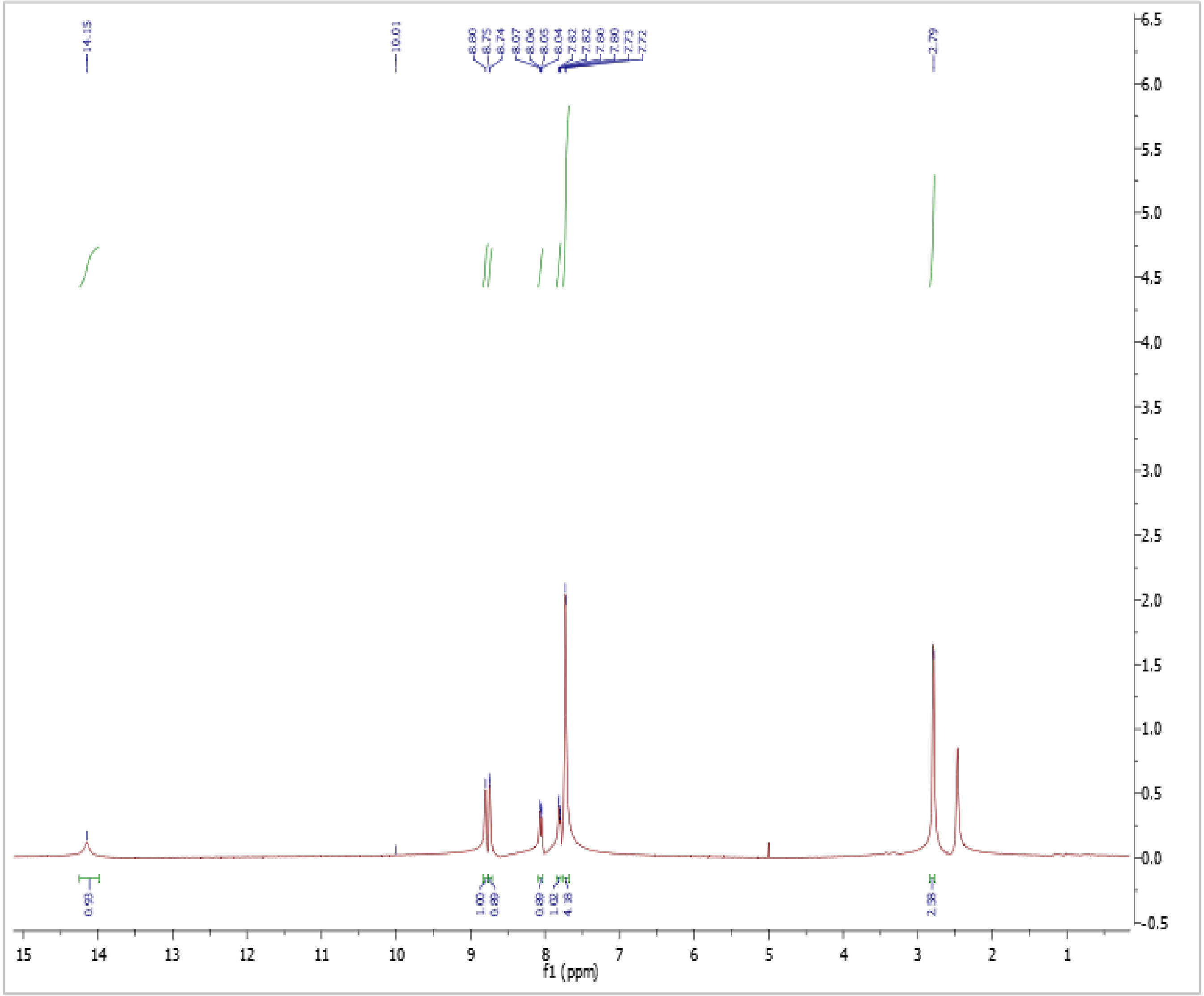

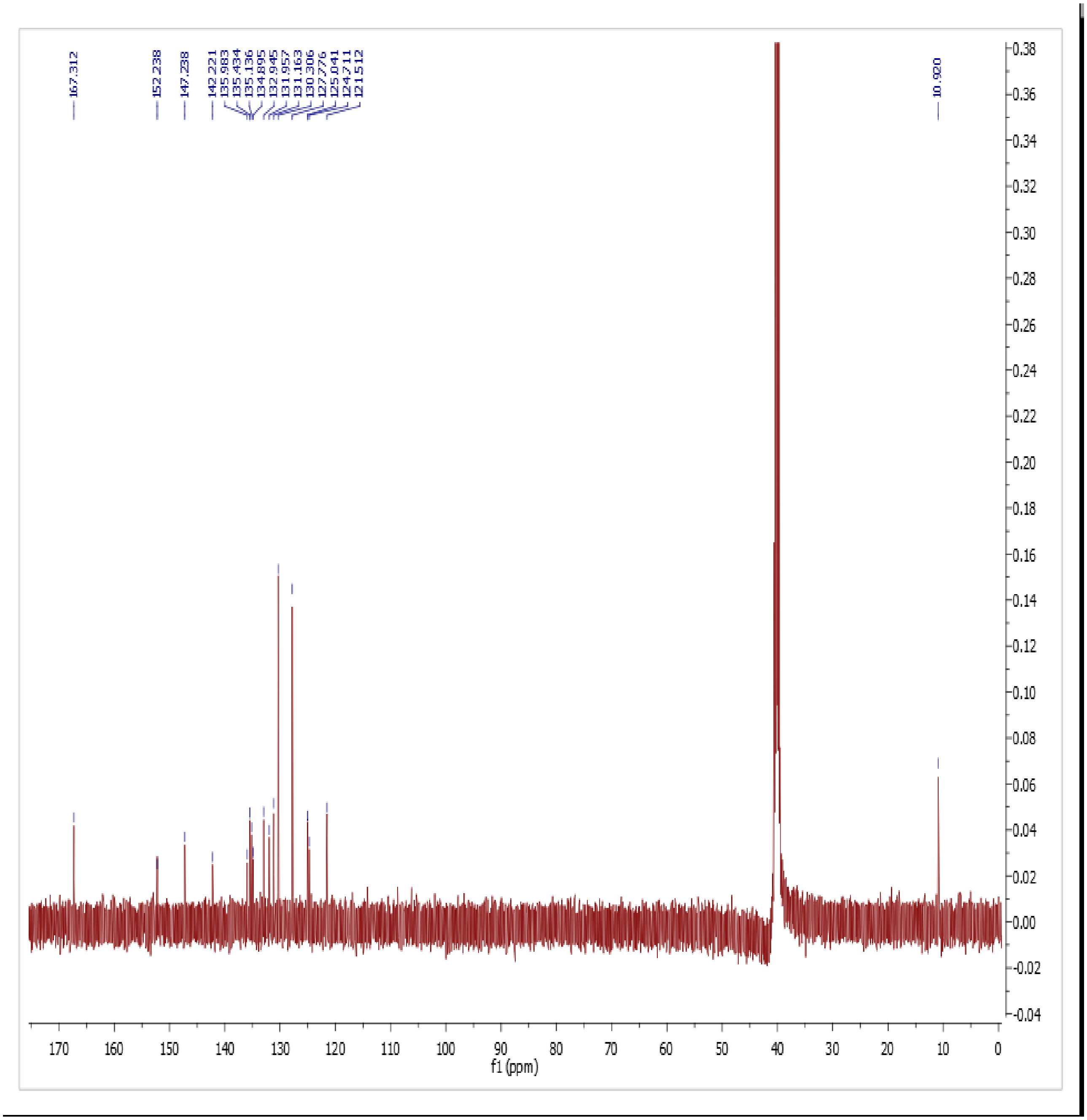

167.312
152.238
147.238
142.221
135.983
135.434
135.136
134.895
132.945
131.957
131.163
130.306
127.776
125.041
124.711
121.512
10.920
f1 (ppm)

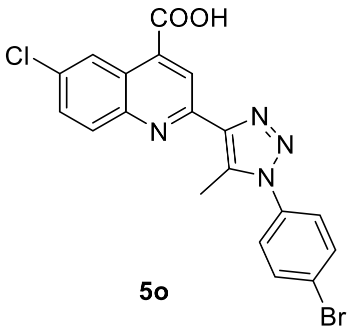

5o

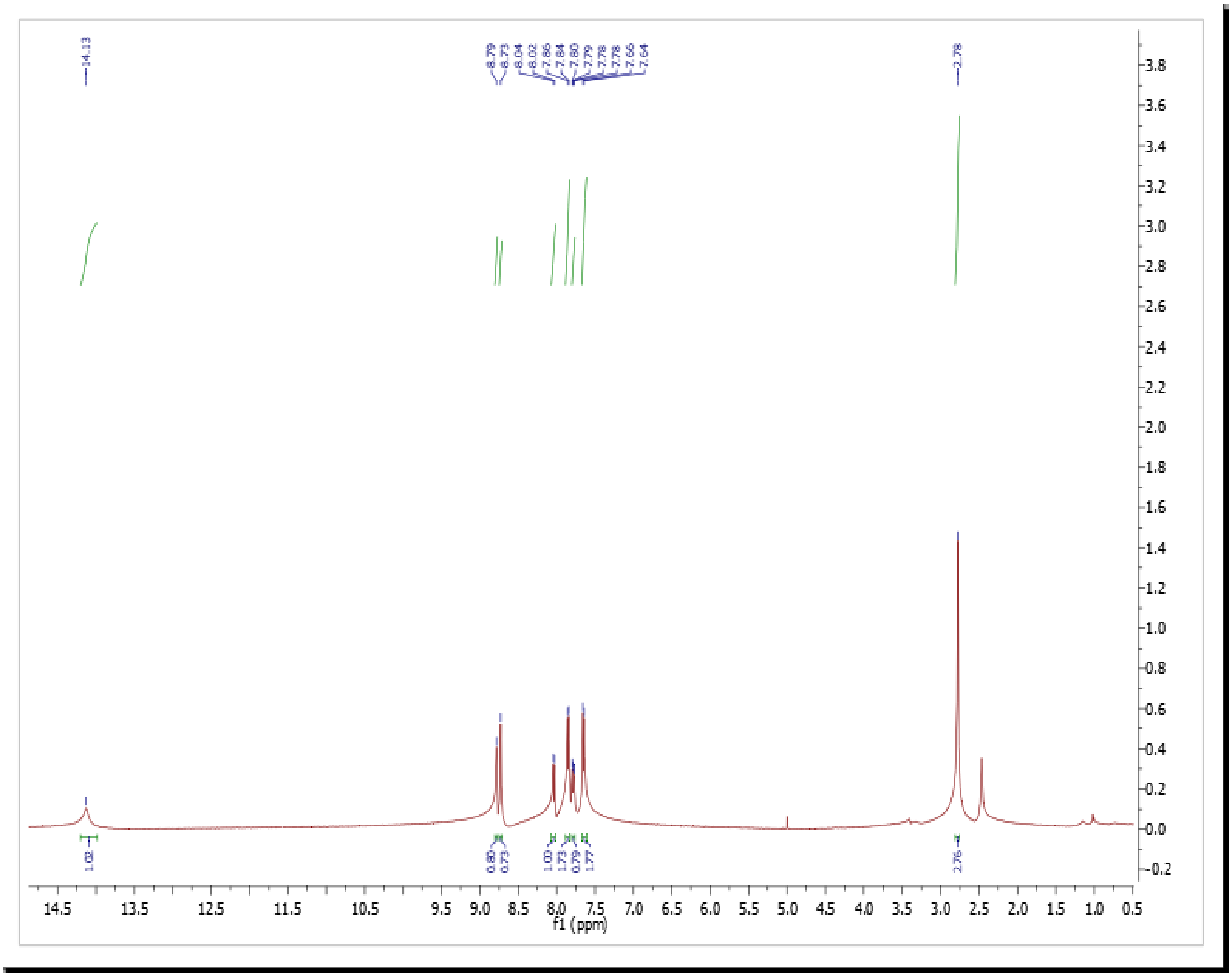

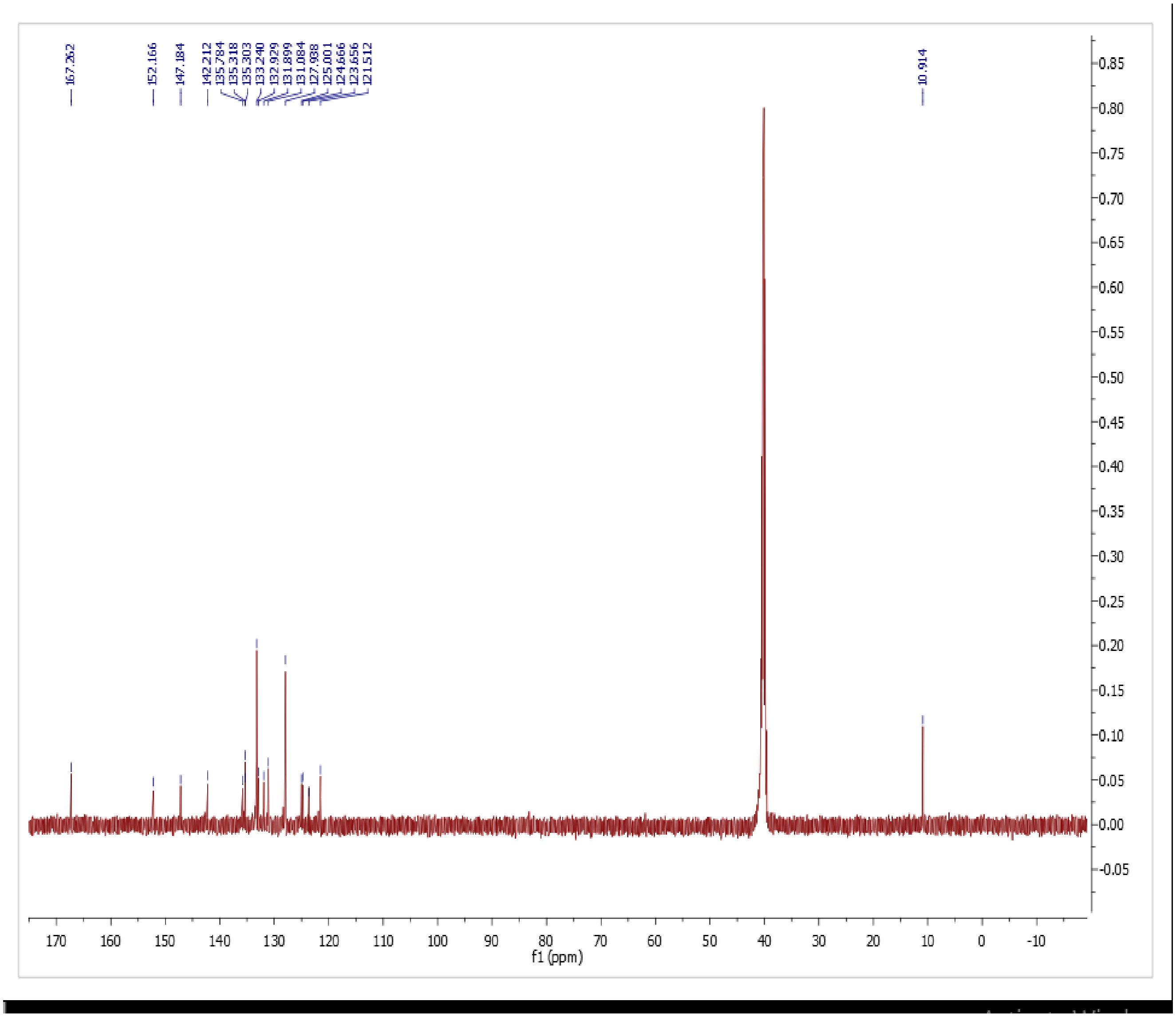
167.262
152.166
147.184
142.212
135.784
135.318
135.303
133.240
132.929
131.899
131.084
127.938
125.001
124.666
123.656
121.512
10.914
f1 (ppm)

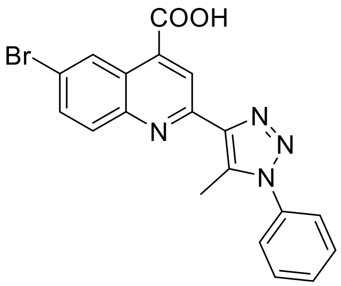
COOH
Br
N
N
N
N

5q

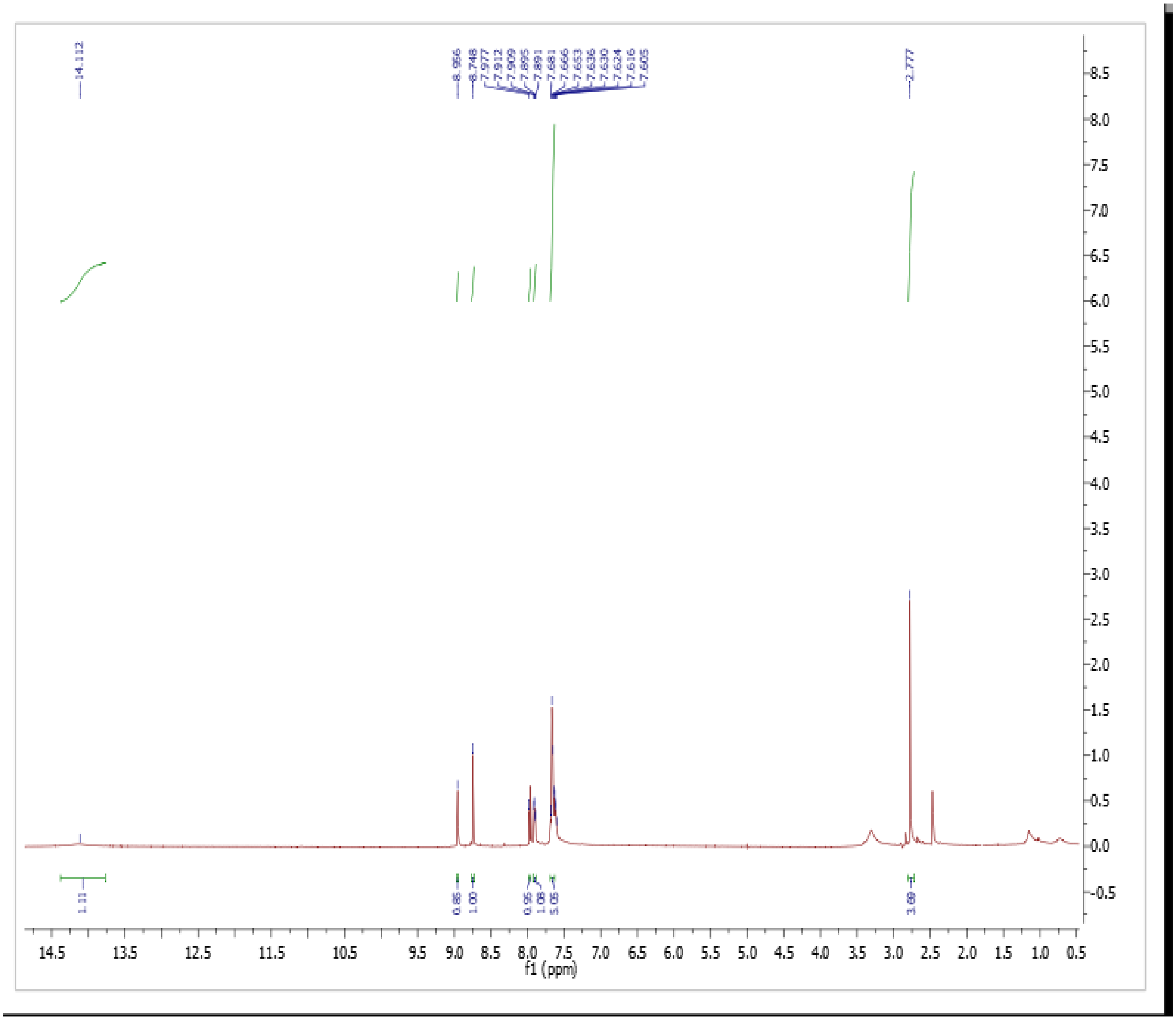
14.112
8.956
8.748
7.977
7.912
7.909
7.895
7.891
7.681
7.666
7.653
7.636
7.630
7.624
7.616
7.605
2.777
1.11
0.85
1.00
0.95
1.08
5.05
3.09
14.5 13.5 12.5 11.5 10.5 9.5 9.0 8.5 8.0 7.5 7.0 6.5 6.0 5.5 5.0 4.5 4.0 3.5 3.0 2.5 2.0 1.5 1.0 0.5
f1 (ppm)
8.5 8.0 7.5 7.0 6.5 6.0 5.5 5.0 4.5 4.0 3.5 3.0 2.5 2.0 1.5 1.0 0.5 0.0 -0.5

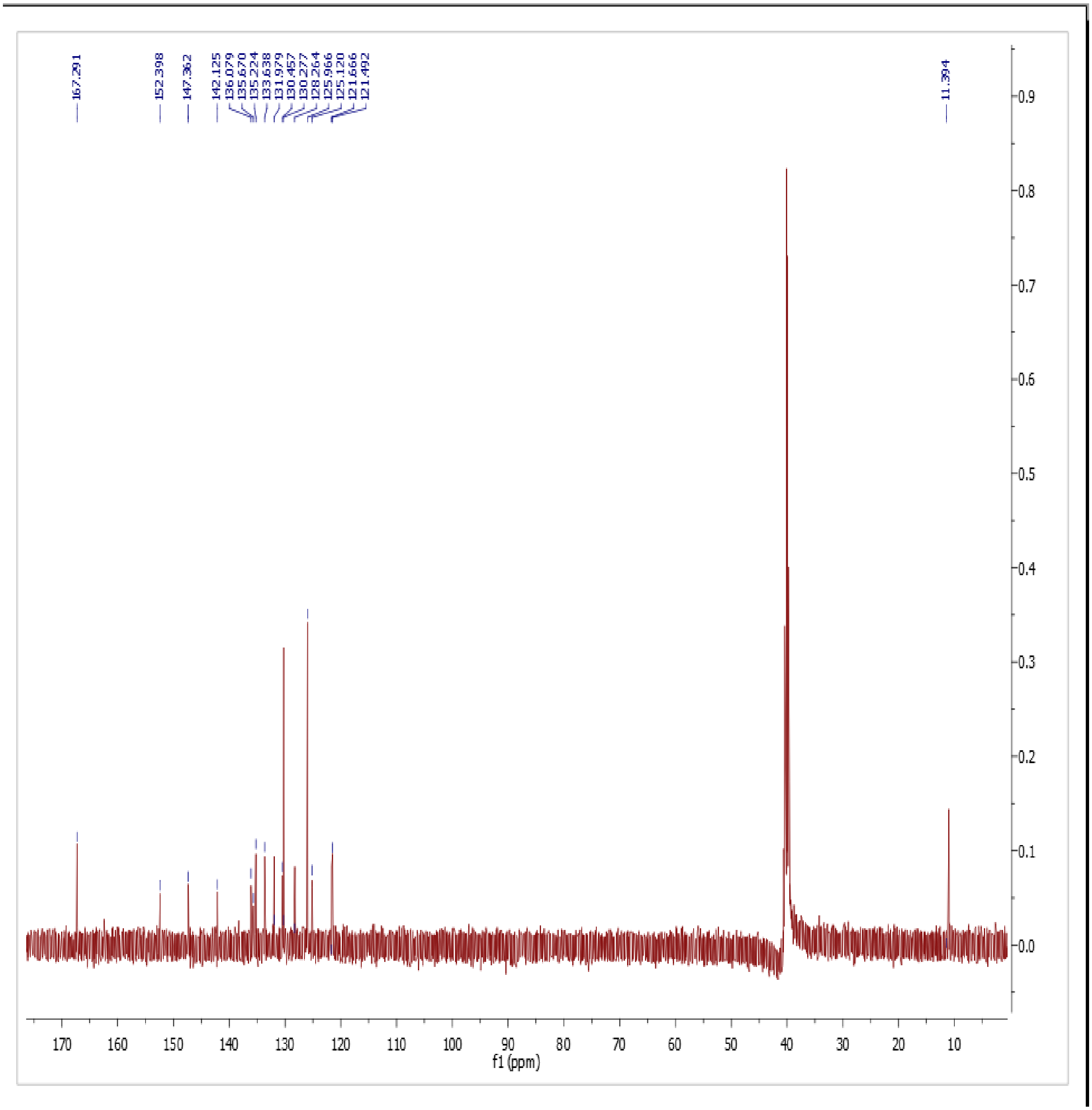
167.291
152.398
147.362
142.125
136.079
135.670
135.224
133.638
131.979
130.457
130.277
128.264
125.966
125.120
121.666
121.492
11.394
f1 (ppm)

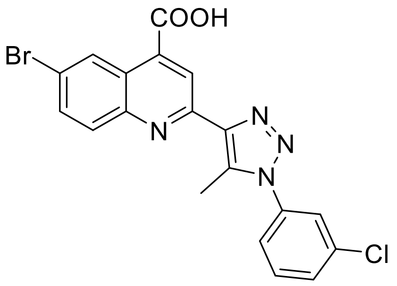
COOH
Br
N
N
N
N
Cl

5s

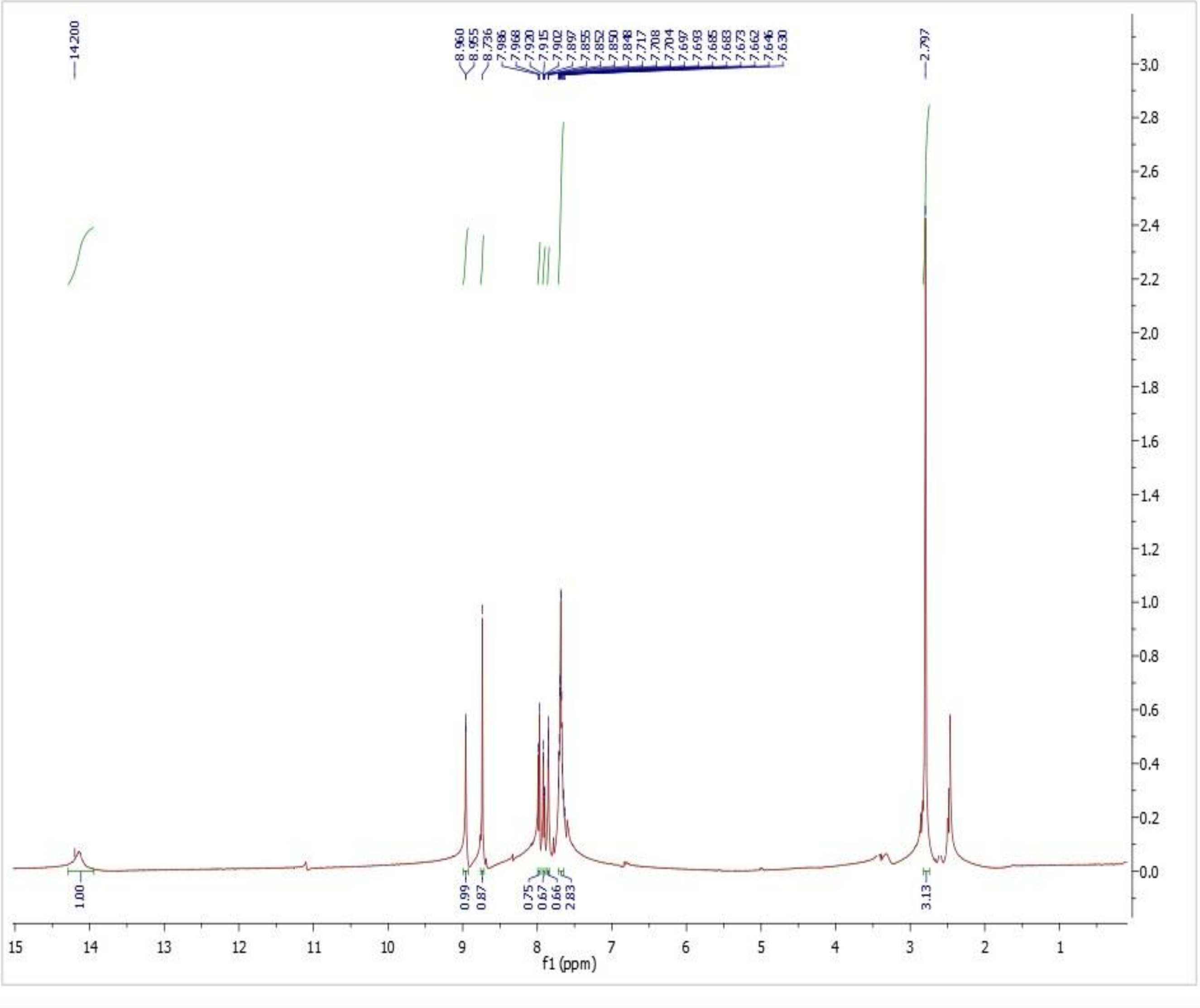
14.200
8.960
8.955
8.736
7.986
7.968
7.920
7.915
7.902
7.897
7.855
7.852
7.850
7.848
7.717
7.708
7.704
7.697
7.693
7.685
7.683
7.673
7.662
7.646
7.630
2.797
1.00
0.99
0.87
0.75
0.67
0.66
2.83
3.13
f1 (ppm)

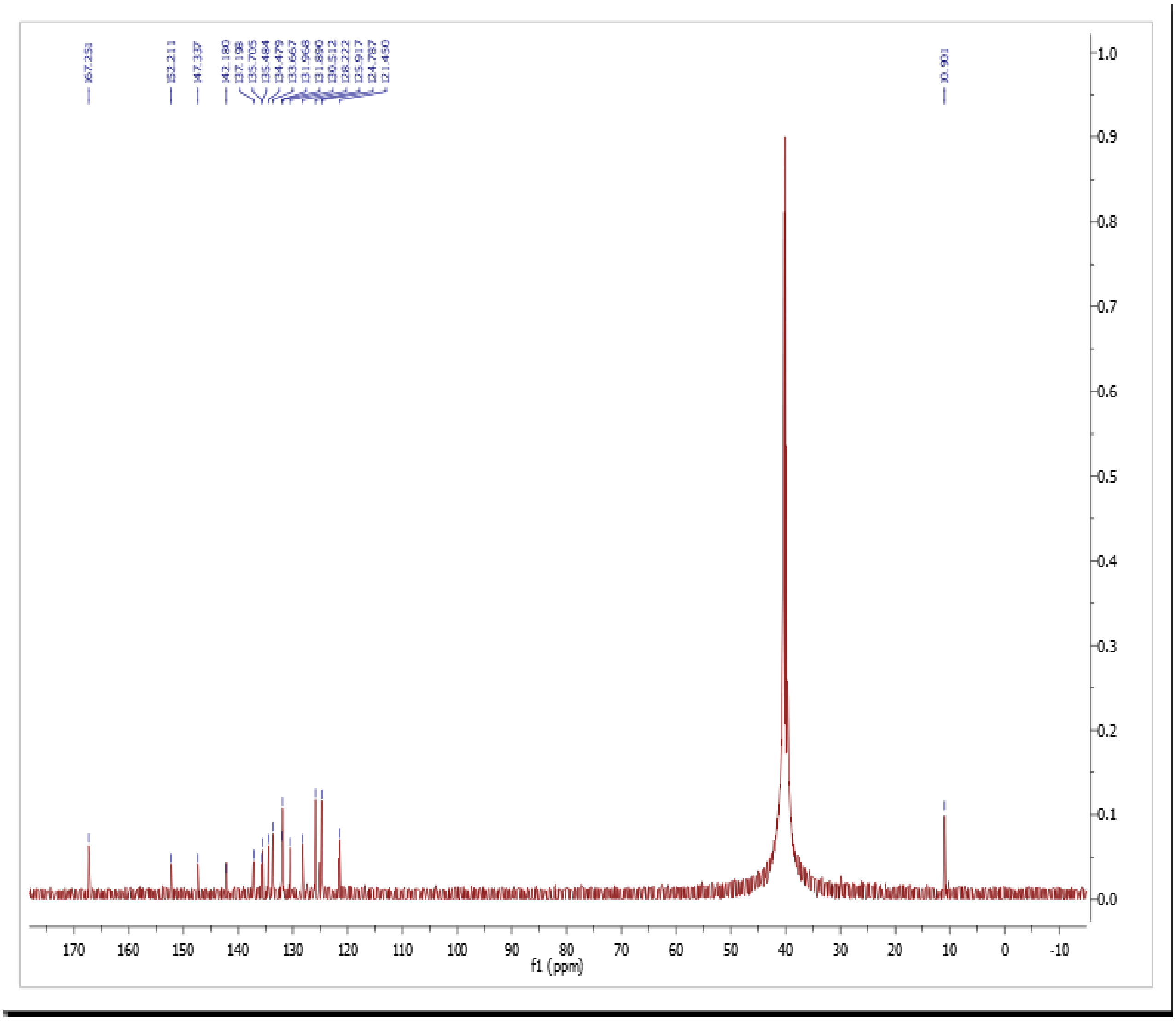

167.251
152.211
147.337
142.180
137.198
135.705
135.484
134.479
133.667
131.968
131.890
130.512
128.222
125.917
124.787
121.450
10.901
1.0
0.9
0.8
0.7
0.6
0.5
0.4
0.3
0.2
0.1
0.0
170 160 150 140 130 120 110 100 90 80 70 60 50 40 30 20 10 0 -10
f1 (ppm)

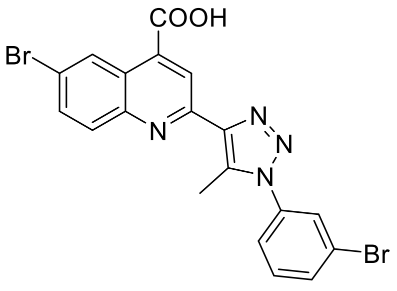

5t

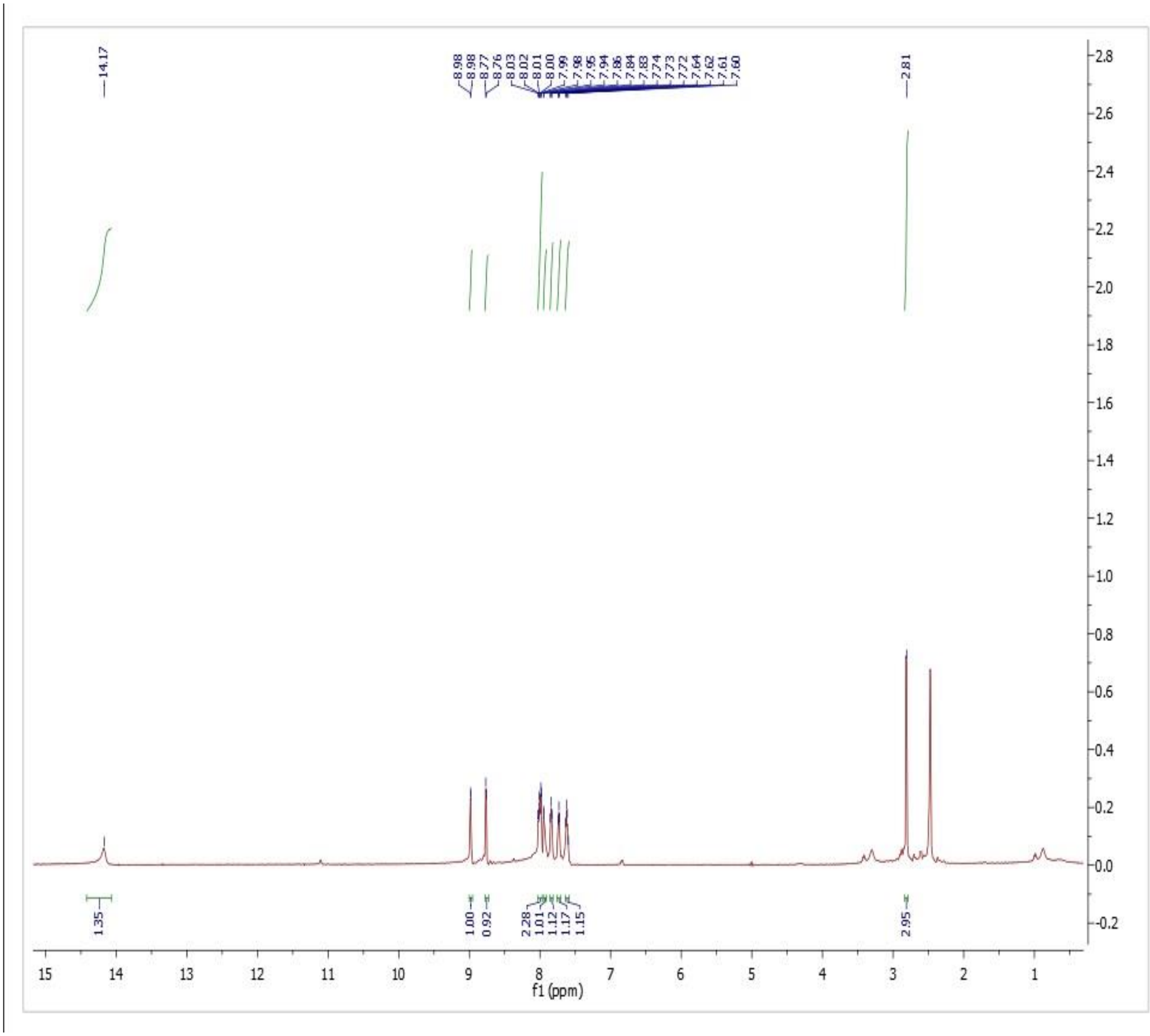

14.17
8.98 8.98 8.77 8.76 8.03 8.02 8.01 8.00 7.99 7.98 7.95 7.94 7.86 7.84 7.83 7.74 7.73 7.72 7.64 7.62 7.61 7.60
2.81
1.35
1.00
0.92
2.28
1.01
1.12
1.17
1.15
2.95
f1 (ppm)

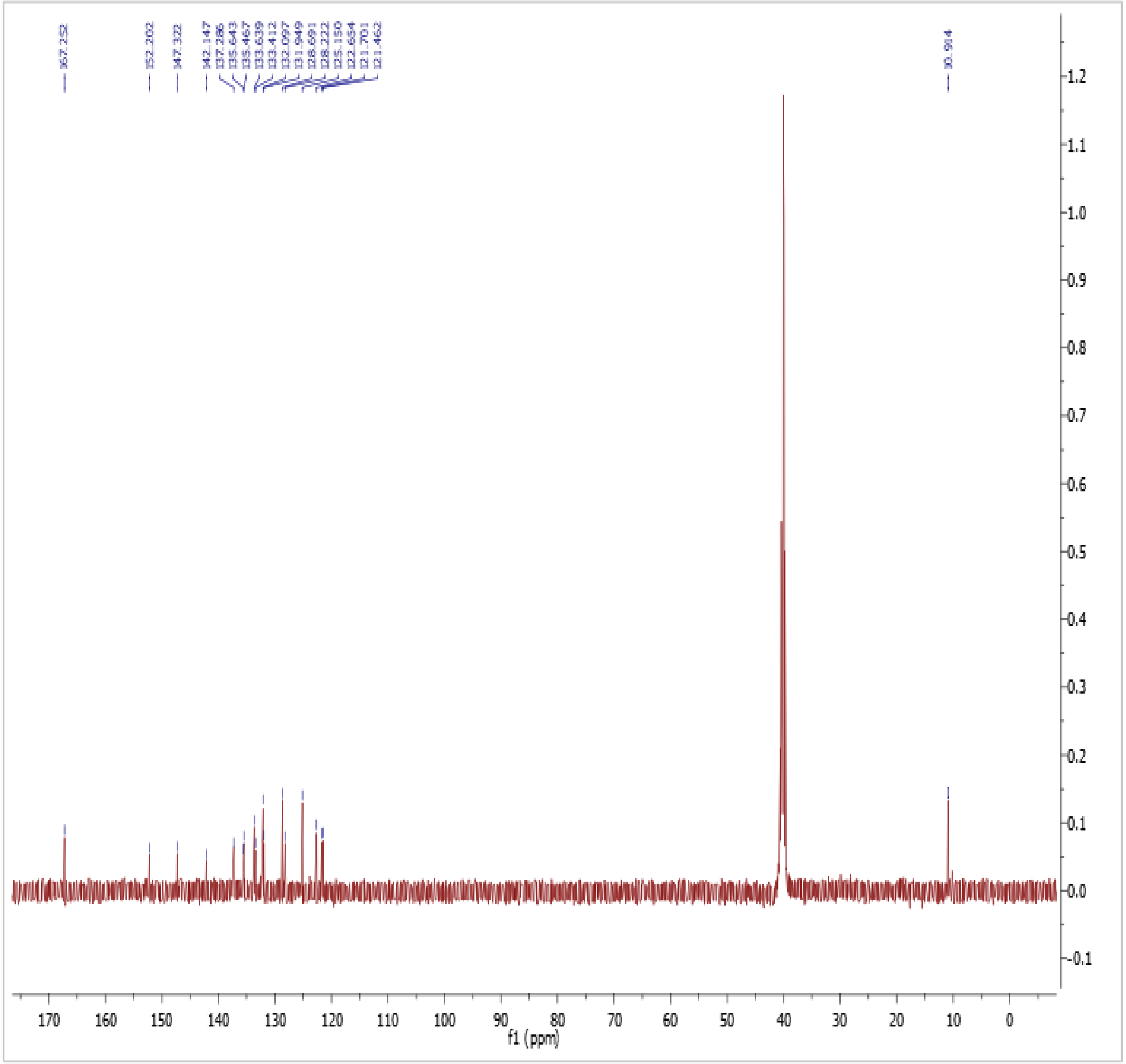

167.252
152.202
147.322
142.147
137.286
135.643
135.467
133.639
133.412
132.097
131.949
128.691
128.222
125.150
122.654
121.701
121.462
10.914
1.2
1.1
1.0
0.9
0.8
0.7
0.6
0.5
0.4
0.3
0.2
0.1
0.0
-0.1
170 160 150 140 130 120 110 100 90 80 70 60 50 40 30 20 10 0
f1 (ppm)

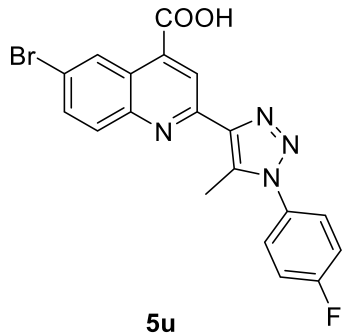
COOH
Br
N
N
N
N
F

5u

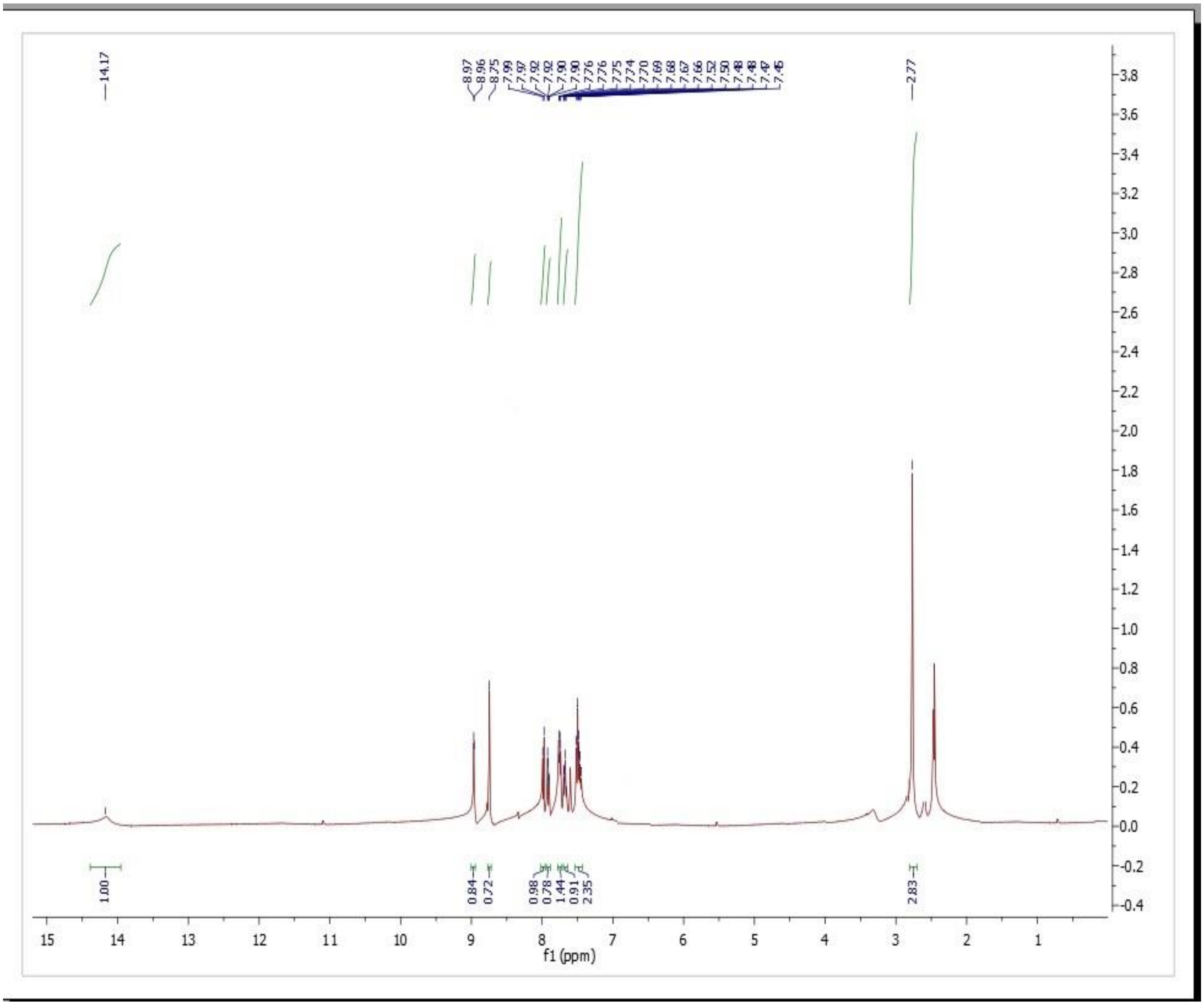
14.17
8.97
8.96
8.75
7.99
7.97
7.92
7.92
7.90
7.90
7.76
7.76
7.75
7.74
7.70
7.69
7.68
7.67
7.66
7.52
7.50
7.48
7.48
7.47
7.45
2.77
1.00
0.84
0.72
0.98
0.78
1.44
0.91
2.35
2.83
f1 (ppm)

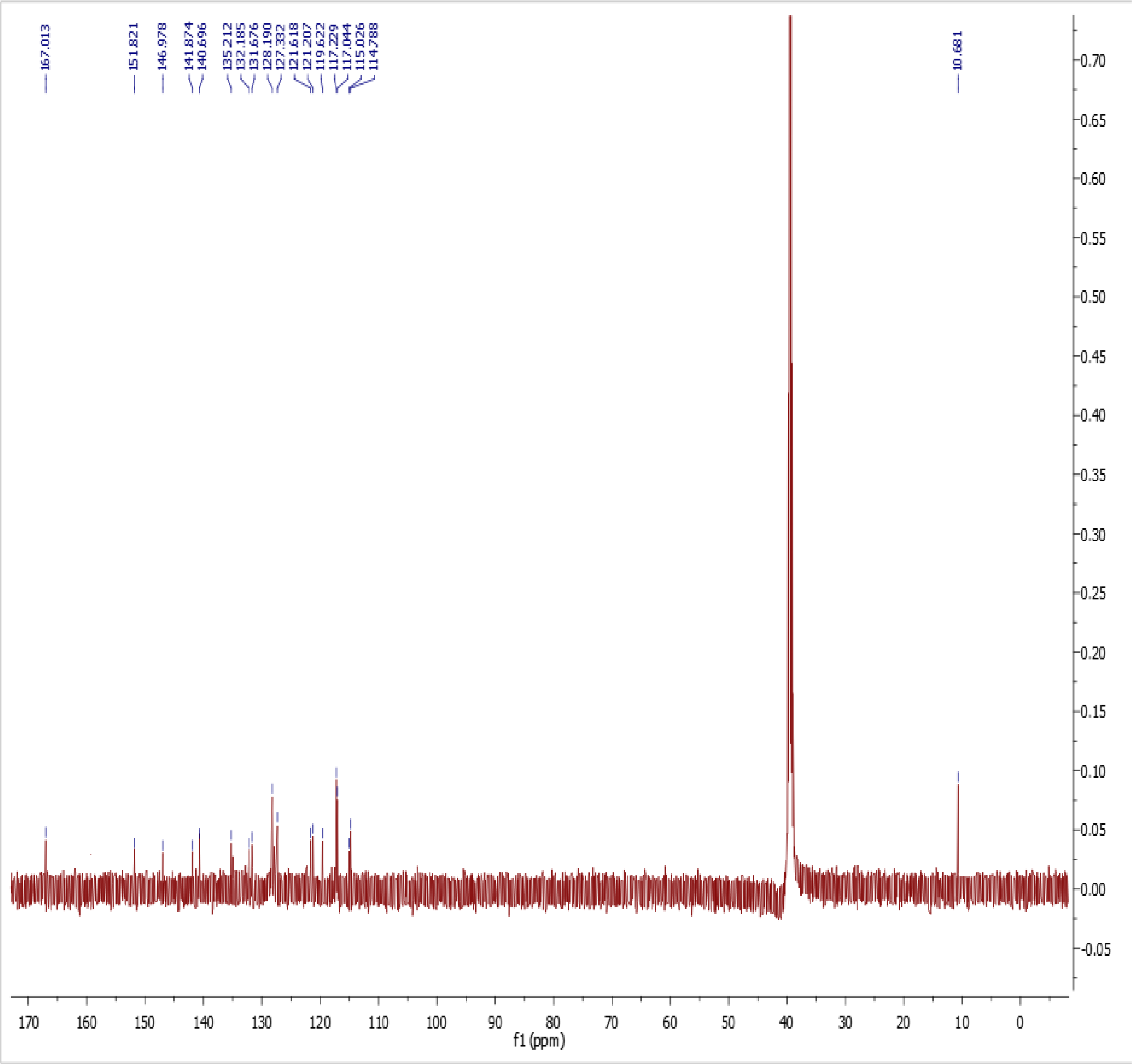
167.013
151.821
146.978
141.874
140.696
135.212
132.185
131.676
128.190
127.332
121.618
121.207
119.622
117.229
117.044
115.026
114.788
10.681
f1 (ppm)

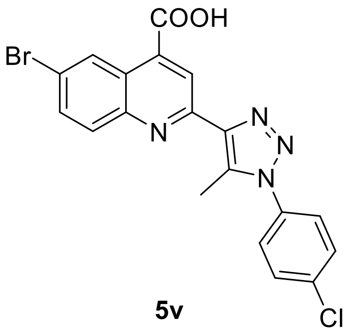

5v

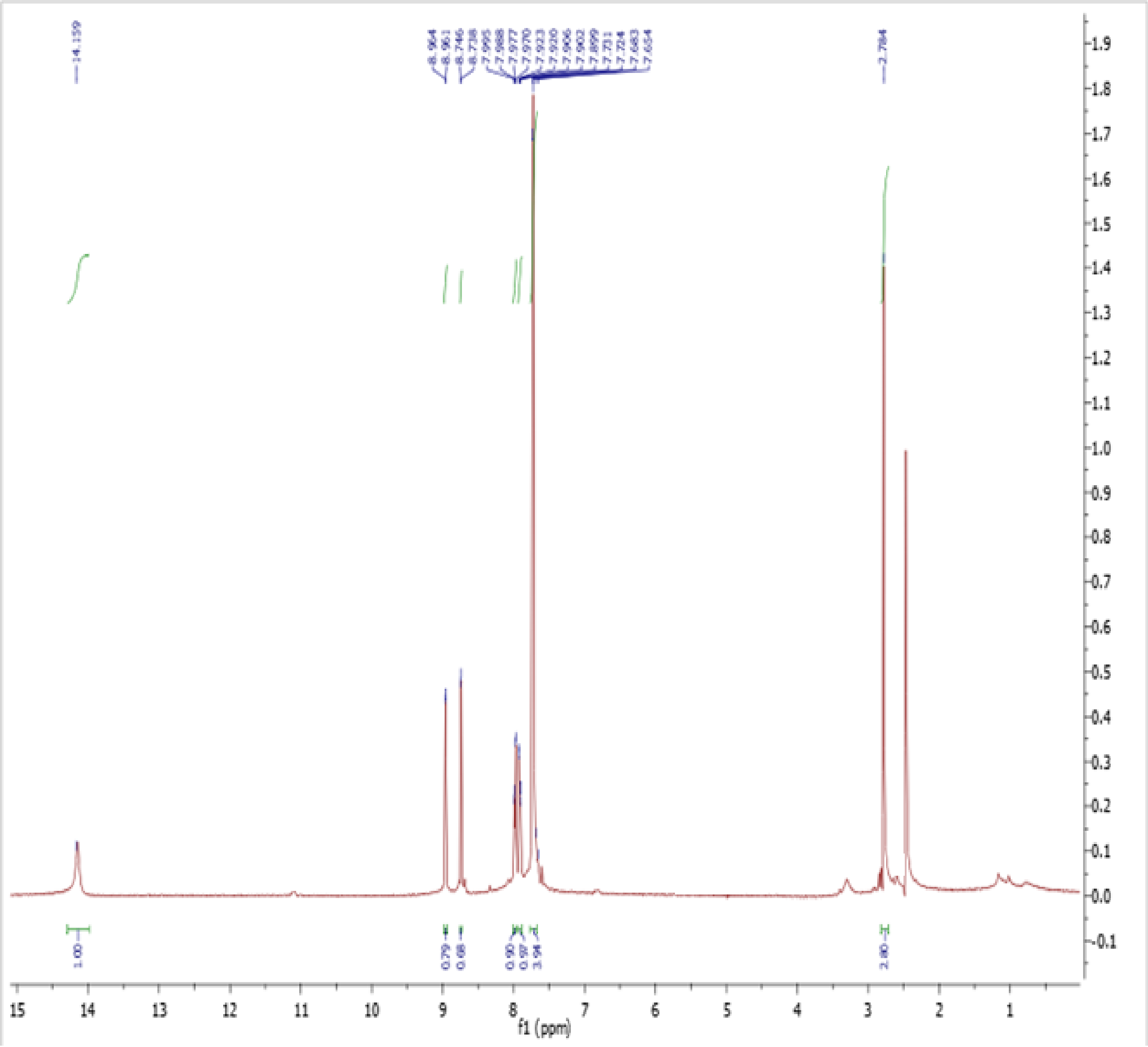

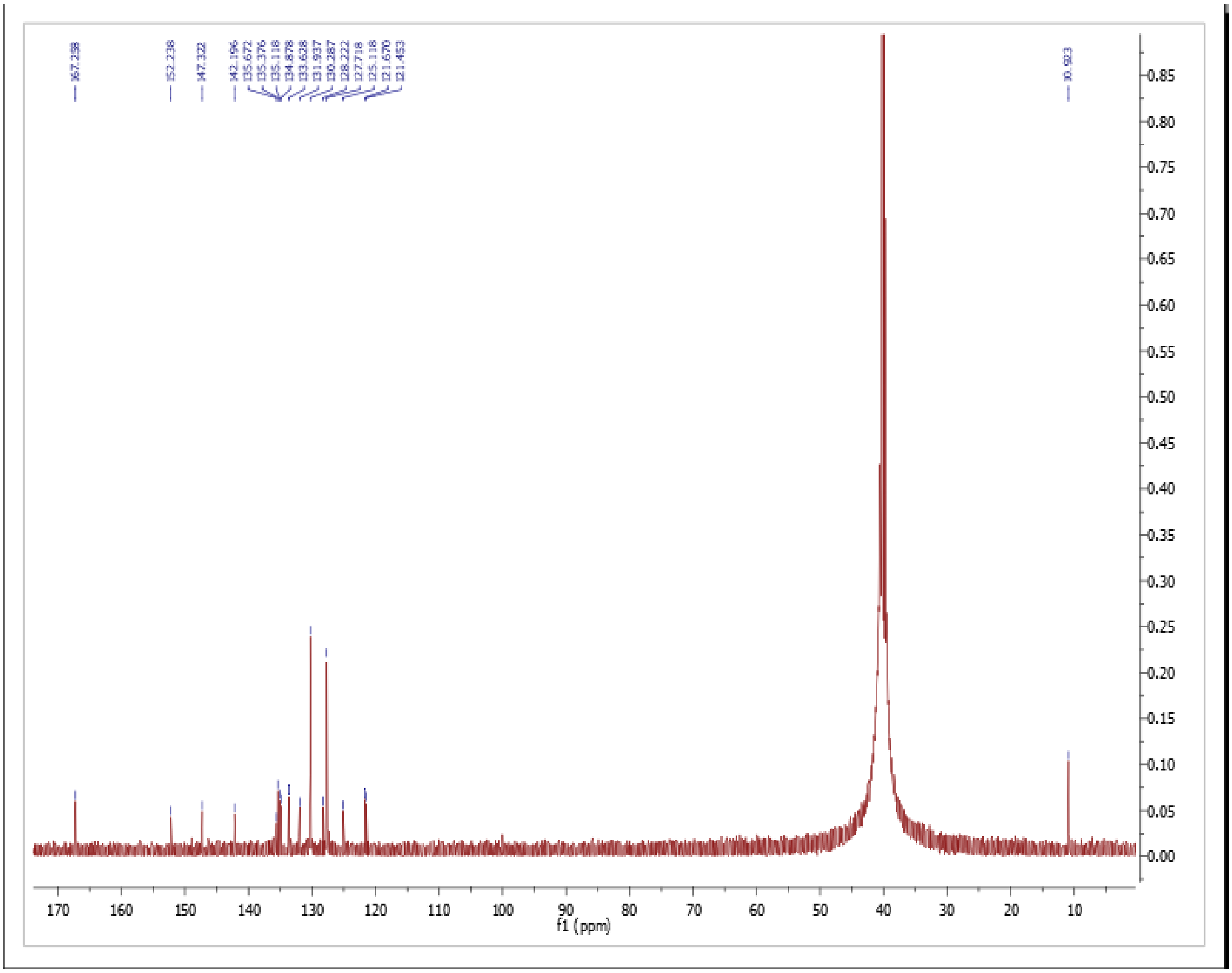
167.258
152.238
147.322
142.196
135.672
135.376
135.118
134.878
133.628
131.937
130.287
128.222
127.718
125.118
121.670
121.453
10.923
0.85
0.80
0.75
0.70
0.65
0.60
0.55
0.50
0.45
0.40
0.35
0.30
0.25
0.20
0.15
0.10
0.05
0.00
170
160
150
140
130
120
110
100
90
80
70
60
50
40
30
20
10
f1 (ppm)

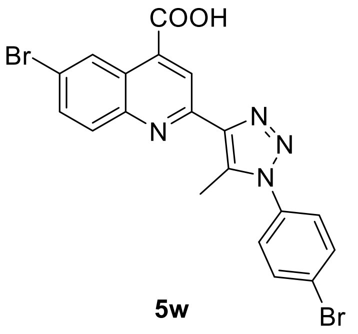

5w

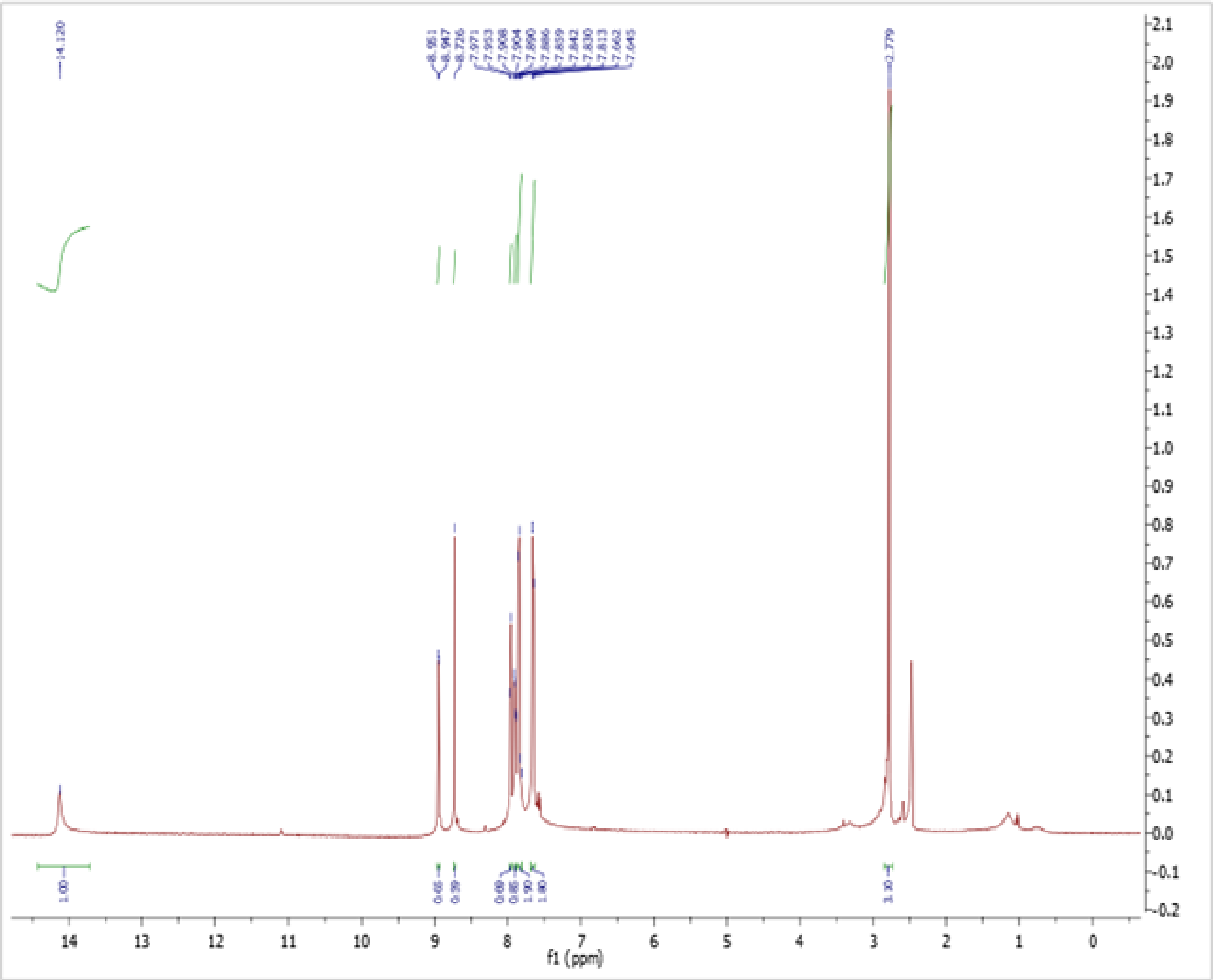

14.120
8.961
8.947
8.726
7.971
7.953
7.908
7.904
7.890
7.886
7.859
7.842
7.830
7.813
7.662
7.645
2.779
1.00
0.66
0.59
0.69
0.86
1.90
1.80
3.30
f1 (ppm)

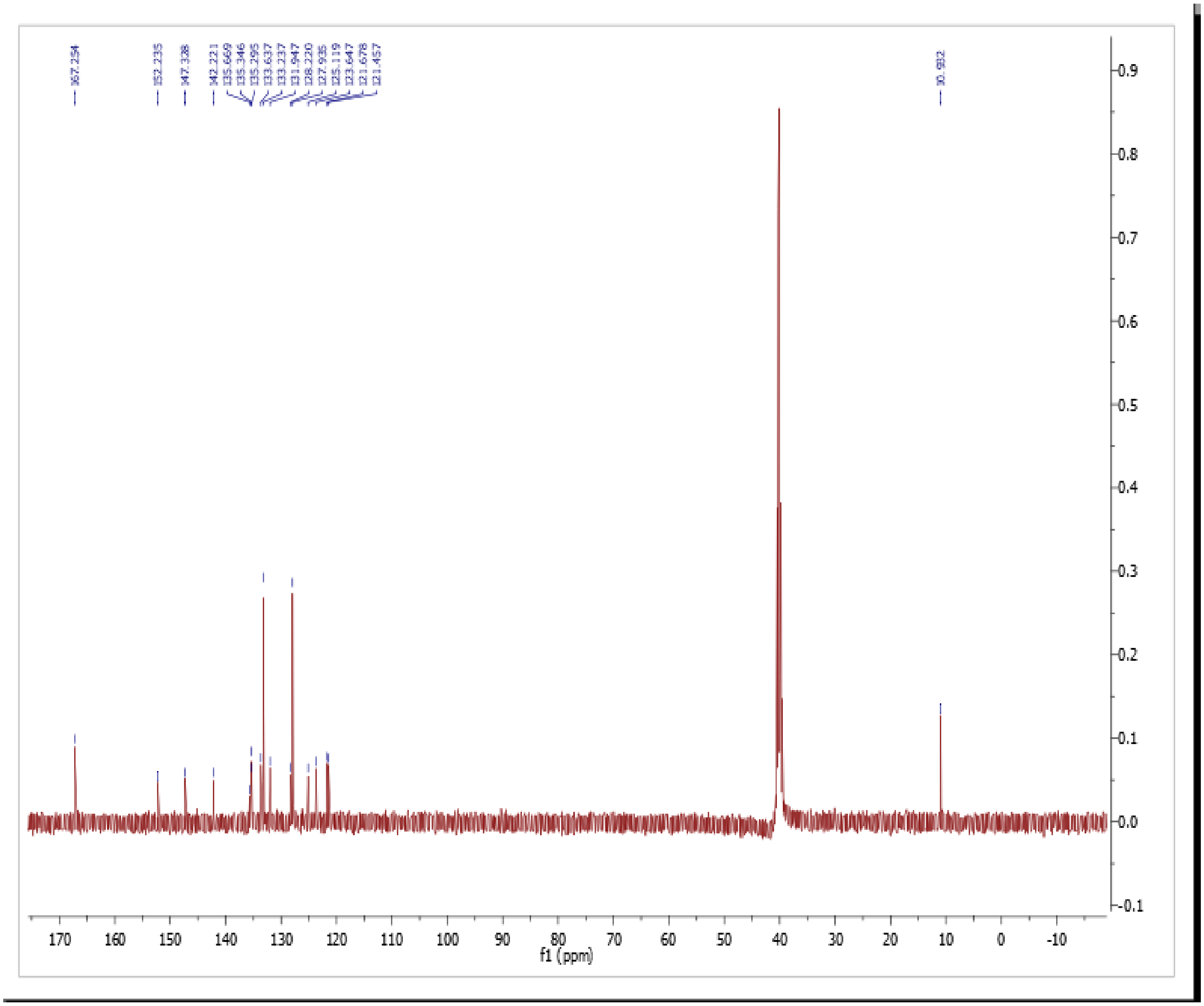

167.254
152.235
147.338
142.221
135.669
135.346
135.295
133.637
133.237
131.947
128.220
127.935
125.119
123.647
121.678
121.457
10.932
f1 (ppm)

Data File C:\CHEM32\1\DATA\5G-002.D
Sample Name: 5g

===

Acq. Operator : SYSTEM
Sample Operator : SYSTEM
Acq. Instrument : HPLC Location : Vial 3
Injection Date : 5/16/2024 12:58:20 AM
Inj Volume : 5.000 µl
Acq. Method : C:\CHEM32\1\METHODS\PURE.M
Last changed : 5/16/2024 1:05:12 AM by SYSTEM
(modified after loading)
Analysis Method : C:\CHEM32\1\METHODS\PURE.M
Last changed : 5/16/2024 1:30:04 AM by SYSTEM
(modified after loading)
Sample Info : 65 ACN:35 phosphate buffer pH 6.5, Flow 1.50 mL/min, 250 nm, 5 ul injection

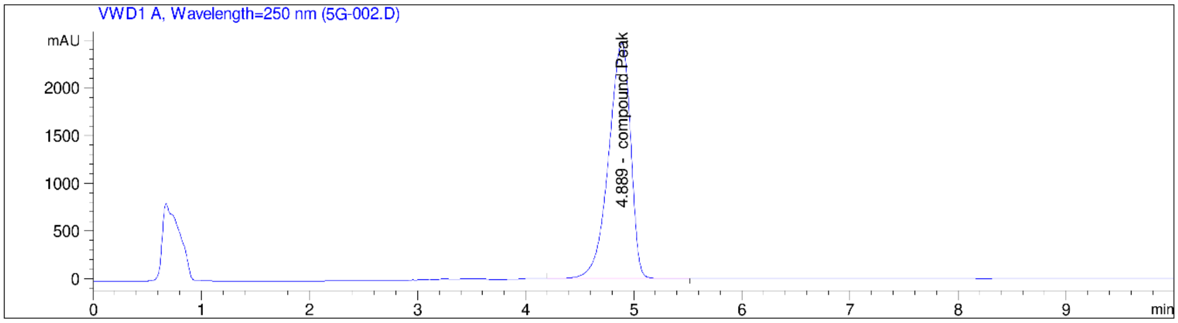

===

Area Percent Report

===

Sorted By : Signal
Calib. Data Modified : 5/16/2024 1:30:04 AM
Multiplier : 1.0000
Dilution : 1.0000
Sample Amount: : 5.00000e-1 [mg/ml] (not used in calc.)
Do not use Multiplier & Dilution Factor with ISTDs

Signal 1: VWD1 A, Wavelength=250 nm

Peak #	RetTime [min]	Type	Width [min]	Area [mAU*s]	Area %	Name
1	4.889	VB	0.2216	3.47754e4	99.1621	compound Peak

Totals : 3.47754e4 99.1621

Data File C:\CHEM32\1\DATA\5I-004.D
Sample Name: 5i

===

Acq. Operator : SYSTEM
Sample Operator : SYSTEM
Acq. Instrument : HPLC   Location : Vial 5
Injection Date : 5/16/2024 1:23:00 AM
Inj Volume : 5.000 µl
Acq. Method : C:\CHEM32\1\METHODS\PURE.M
Last changed : 5/16/2024 1:22:20 AM by SYSTEM
(modified after loading)
Analysis Method : C:\CHEM32\1\METHODS\PURE.M
Last changed : 5/16/2024 1:36:13 AM by SYSTEM
(modified after loading)
Sample Info : 65 ACN:35 phosphate buffer pH 6.5, Flow 1.50 mL/min, 250 nm, 5 ul injection

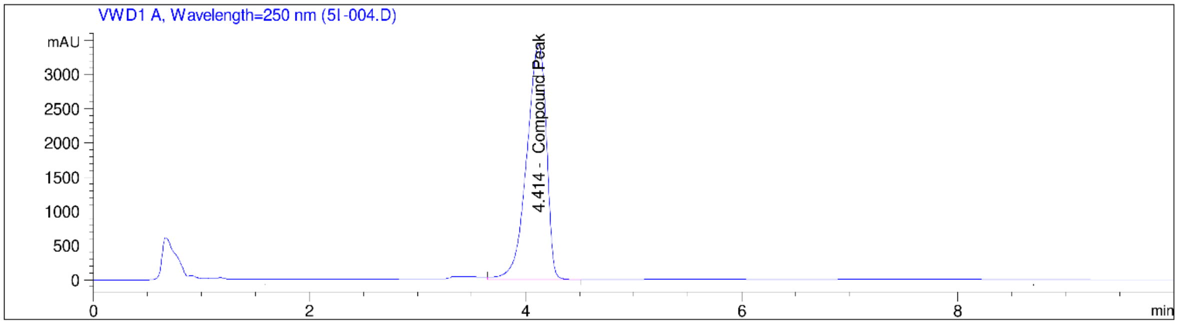

===

Area Percent Report

===

Sorted By : Signal
Calib. Data Modified : 5/16/2024 1:36:13 AM
Multiplier : 1.0000
Dilution : 1.0000
Sample Amount: : 5.00000e-1 [mg/ml] (not used in calc.)
Do not use Multiplier & Dilution Factor with ISTDs

Signal 1: VWD1 A, Wavelength=250 nm

Peak #	RetTime [min]	Type	Width [min]	Area [mAU*s]	Area %	Name
1	4.414	VB	0.1978	4.34462e4	98.5867	Compound Peak

Totals : 4.34462e4 98.5867

Data File C:\CHEM32\1\DATA\5N-005.D
Sample Name: 5n

===

Acq. Operator : SYSTEM
Sample Operator : SYSTEM
Acq. Instrument : HPLC Location : Vial 7
Injection Date : 5/16/2024 1:39:26 AM
Inj Volume : 5.000 µl
Acq. Method : C:\CHEM32\1\METHODS\PURE.M
Last changed : 5/16/2024 1:47:30 AM by SYSTEM
(modified after loading)
Analysis Method : C:\CHEM32\1\METHODS\PURE.M
Last changed : 5/16/2024 1:55:26 AM by SYSTEM
(modified after loading)
Sample Info : 65 ACN:35 phosphate buffer pH 6.5, Flow 1.50 mL/min, 250 nm, 5 ul injection

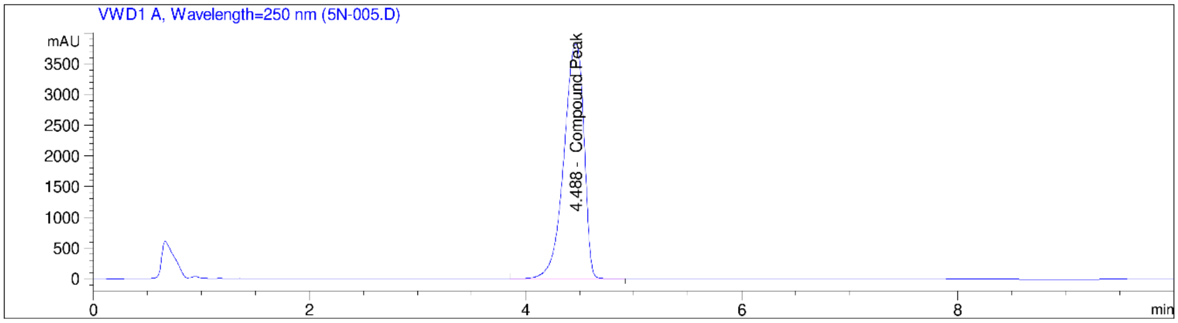

===

Area Percent Report

===

Sorted By : Signal
Calib. Data Modified : 5/16/2024 1:55:26 AM
Multiplier : 1.0000
Dilution : 1.0000
Sample Amount: : 5.00000e-1 [mg/ml] (not used in calc.)
Do not use Multiplier & Dilution Factor with ISTDs

Signal 1: VWD1 A, Wavelength=250 nm

Peak #	RetTime [min]	Type	Width [min]	Area [mAU*s]	Area %	Name
1	4.488	VB	0.2041	4.91655e4	98.6968	Compound Peak

Totals : 4.91655e4 98.6968
